# Causal Association Between Birth Weight and Adult Diseases: Evidence From a Mendelian Randomization Analysis

**DOI:** 10.3389/fgene.2019.00618

**Published:** 2019-07-10

**Authors:** Ping Zeng, Xiang Zhou

**Affiliations:** ^1^Department of Epidemiology and Biostatistics, Xuzhou Medical University, Xuzhou, Jiangsu, China; ^2^Department of Biostatistics, University of Michigan, Ann Arbor, MI, United States; ^3^Center for Statistical Genetics, University of Michigan, Ann Arbor, MI, United States

**Keywords:** birth weight, adult diseases, Mendelian randomization, causal association, genome wide association study, type 2 diabetes, coronary artery disease, myocardial infarction

## Abstract

**Purpose:** Birth weight has a profound long-term impact on individual’s predisposition to various diseases at adulthood—a hypothesis commonly referred to as the fetal origins of adult diseases. However, it is not fully clear to what extent the fetal origins of adult diseases hypothesis holds and it is also not completely known what types of adult diseases are causally affected by birth weight.

**Materials and methods:** Mendelian randomization using multiple genetic instruments associated with birth weight was performed to explore the causal relationship between birth weight and adult diseases. The causal relationship between birth weight and 21 adult diseases as well as 38 other complex traits was examined based on data collected from 37 large-scale genome-wide association studies with up to 340,000 individuals of European ancestry. Causal effects of birth weight were estimated using inverse-variance weighted methods. The identified causal relationships between birth weight and adult diseases were further validated through extensive sensitivity analyses, bias calculation, and simulations.

**Results:** Among the 21 adult diseases, three were identified to be inversely causally affected by birth weight after the Bonferroni correction. The measurement unit of birth weight was defined as its standard deviation (i.e., 488 g), and one unit lower birth weight was causally related to an increased risk of coronary artery disease (CAD), myocardial infarction (MI), type 2 diabetes (T2D), and BMI-adjusted T2D, with the estimated odds ratios of 1.34 [95% confidence interval (CI) 1.17–1.53], 1.30 (95% CI 1.13–1.51), 1.41 (95% CI 1.15–1.73), and 1.54 (95% CI 1.25–1.89), respectively. All these identified causal associations were robust across various sensitivity analyses that guard against various confounding due to pleiotropy or maternal effects as well as reverse causation. In addition, analysis on 38 additional complex traits did not identify candidate traits that may mediate the causal association between birth weight and CAD/MI/T2D.

**Conclusions:** The results suggest that lower birth weight is causally associated with an increased risk of CAD, MI, and T2D in later life, supporting the fetal origins of adult diseases hypothesis.

## Introduction

Birth weight is a widely used surrogate measurement of intrauterine exposure (Peck et al., 2003) and early life development (Scharf et al., 2016), and is an indicator of intergenerational influences (Hackman et al., 1983). It has long been hypothesized that birth weight has a profound long-term impact on an individual’s predisposition to various diseases at adulthood—a hypothesis commonly referred to as the fetal origins of adult diseases (Barker, 1990; Lucas et al., 1999; Barker et al., 2002), which complements the selection *in utero* hypothesis (Bruckner and Catalano, 2018). Indeed, early registry and other observational studies have provided strong empirical evidence supporting an inverse association between birth weight and the risks of several adult diseases (Barker, 1990; Lucas et al., 1999; Barker et al., 2002; Barker, 2004). Exemplary birth weight negatively associated diseases include lung disease (Barker et al., 1991), coronary artery disease (CAD) and stroke (Lawlor et al., 2005), blood pressure (Curhan et al., 1996; Law and Shiell, 1996), type 2 diabetes (T2D) (Harder et al., 2007), and asthma (Mu et al., 2014). In addition, recent studies have also revealed positive associations between birth weight and various types of cancers. Exemplary birth weight-associated cancers include renal cell cancer (Bergstrom et al., 2001), colorectal cancer (Sandhu et al., 2002), primary central nervous system tumor (Georgakis et al., 2017), prostate cancer (Zhou et al., 2016), bone tumor (Chen et al., 2015), and breast cancer (Xu et al., 2009). However, it remains unclear whether the identified associations between birth weight and the aforementioned adult diseases represent truly causal relationship, or are merely spurious associations caused by common confounding factors that occur during prenatal life (Barker, 1990; Leon, 1998; Law, 2002; Ruiz-Narvaez et al., 2014; Kahn et al., 2017) or confounding due to pleiotropy and shared genetic components (Lawlor et al., 2017). Common confounding factors, such as family socioeconomic position, parental education levels, or maternal lifestyles, can be associated with both birth weight and adult diseases and thus cause spurious association between the latter two. These confounding factors are often difficult to fully control for in observational studies (Lucas et al., 1999). As a consequence, some identified associations between birth weight and adult diseases in early studies have not been validated in recent studies. For example, the inverse association between birth weight and adult blood pressure identified in early studies are later found to be a consequence of failure to adjust for adult weight or other factors (Leon, 1998; Lucas et al., 1999). As another example, potentially due to different confounding/mediation effects, different studies show conflicting results with regard to the association between birth weight and T2D: T2D risk is positively associated with birth weight in some studies but negatively associated with birth weight in others (Johnsson et al., 2015; Beaumont et al., 2017). Even in a previous twins-pair study that may well control for possible confounding due to socioeconomic, environmental, and genetic factors, it is not fully understood whether birth weight is causally associated with CAD or stroke in adulthood (Oberg et al., 2011). Therefore, it is not completely clear to what extent the fetal origins of adult diseases hypothesis holds and it is also not completely clear what types of adult diseases are causally affected by birth weight (Law, 2002; Kahn et al., 2017).

**Figure 1:**
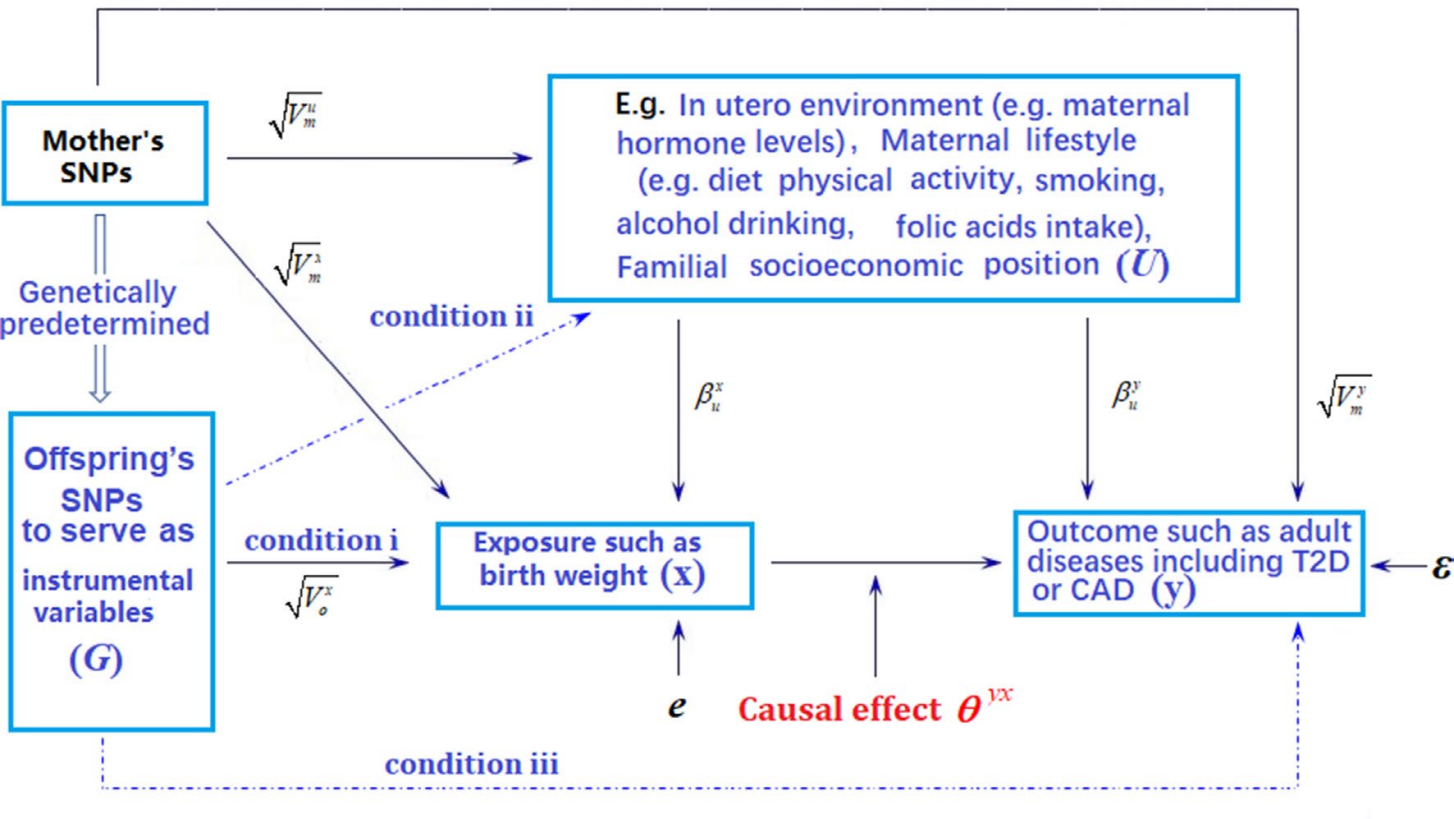
Graphical illustration of Mendelian randomization (MR) analysis. Arrows or dot lines represent the presence or absence of associations, respectively. The MR analysis estimates the causal effect of birth weight to adult disease risk in the presence of various measured and unmeasured confounding factors by carefully selecting single nucleotide polymorphisms (SNPs) that are associated with birth weight to serve as instrumental variables. Valid MR requires these selected SNPs to satisfy three conditions: selected SNPs are strongly associated with birth weight (condition i); selected SNPs are not associated with any known or unknown confounders that are associated with both birth weight and disease (condition ii); selected SNPs are independent of adult disease conditional on birth weight (condition iii). Note that the effects of instrumental variables (G) on the exposure of interest (**x**) may be indirect and mediated through mediator variables. Exemplary traits include BMI (body mass index), T2D (type 2 diabetes), and CAD (coronary artery disease). The notations in the figure are defined further in **Text S4**.

Understanding the long-term causal impact of birth weight on individual’s predisposition to various disease risks is important from a public health perspective, as a better understanding can pave ways for using early nutritional intervention that can potentially increase birth weight to reduce disease burden in later life (Ramakrishnan, 2004). Exemplary early nutritional intervention includes iron supplement (Cogswell et al., 2003). However, determining the causal impact of birth weight on various adult diseases through traditional randomized intervention studies is challenging, as such studies necessarily require long-term follow-ups and are time-consuming, expensive, and often times unethical (Lucas, 1998; Eriksson et al., 2001; Lawlor et al., 2017). Therefore, it is desirable to determine the causal relationship between birth weight and various adult diseases through observational studies (Sheehan et al., 2008). A powerful statistical tool to determine causal relationship and estimate causal effects in observational studies is Mendelian randomization (MR). MR adapts the commonly used instrumental variable analysis method developed in the field of causal inference to settings where genetic variants are served as instrumental variables (Angrist et al., 1996; Greenland, 2000). In particular, MR employs genetic variants as proxy indicators (i.e., instrumental variables) for the exposure of interest (i.e., birth weight) and uses these genetic variants to assess the causal effect of the exposure on the outcome variable of interest (i.e., adult diseases) (**Figure 1**) (Sheehan et al., 2008). Because genetic variants are measured with high accuracy and capture long-term effect of the exposure, MR analysis results are often not susceptible to bias caused by measurement errors that are commonly encountered in randomized intervention studies (Haycock et al., 2016). In addition, because the two alleles of a genetic variant are randomly segregated during gamete formation and conception under the Mendel’s law and because such segregation is independent of many known or unknown confounders, MR analysis results are also less susceptible to reverse causation and confounding factors compared with other study designs (Davey Smith and Ebrahim, 2003). As a result, MR has become a popular and cost-effective analysis tool for causal inference in observational studies, avoiding the need to record and control for all possible confounding factors present in the study.

Indeed, MR studies have been recently carried out to investigate the causal effect of birth weight on either CAD or T2D (e.g., Au Yeung et al., 2016; Wang et al., 2016), each with a relatively small sample size and subsequently a small set of valid instrumental variables. Unfortunately, for CAD, the causality result of birth weight does not hold in follow-up sensitivity analyses and is not robust with respect to the choice of statistical methods (Au Yeung et al., 2016; Wang et al., 2016). For T2D, sensitivity analyses were not carried out in the study (Scott et al., 2017), and it was thus unclear, for example, whether instrumental variable outliers selected in the study may impact the estimation of the causal effect of birth weight. Similar MR analyses on CAD, T2D, and breast cancer also were conducted in more recently published studies (Kar et al., 2018; Zanetti et al., 2018). Here, we perform a large-scale MR study to comprehensively investigate the causal effects of birth weight on a total of 21 diseases and 38 complex traits in adulthood. Our results are validated with a wide range of sensitivity analyses and simulations to ensure result robustness.

## Materials and Methods

We present a brief overview of the analysis procedure with technical details provided in **Text S1**–**Text S4**.

### Data Sources

We first obtained summary statistics in terms of marginal effect size estimate of single nucleotide polymorphism (SNP) and its standard error on birth weight from the Early Growth Genetics (EGG) consortium study (Horikoshi et al., 2016). The EGG consortium study is the largest genome-wide association study (GWAS) to date on birth weight (a continuous trait) and contains association results for 16,245,523 genotyped and imputed SNPs based on up to 153,781 individuals collected from 35 studies (**Table S1**). Next, to examine the causal effect of birth weight on adult diseases, we collected summary statistics from corresponding GWASs for 21 diseases (**Text S1**). These diseases include advanced age-related macular degeneration (AMD) (Fritsche et al., 2016), Alzheimer’s disease (Lambert et al., 2013), Parkinson’s disease (Pankratz et al., 2012), chronic kidney disease (CKD) (Köttgen et al., 2010), celiac disease (Dubois et al., 2010), inflammatory bowel disease (IBD) (Liu et al., 2015), Crohn’s disease (CD) (Liu et al., 2015), ulcerative colitis (UC) (Liu et al., 2015), primary biliary cirrhosis (PBC) (Cordell et al., 2015), primary sclerosing cholangitis (PSC) (Ji et al., 2017), systemic lupus erythematosus (SLE) (Bentham et al., 2015), CAD (Nikpay et al., 2015), myocardial infarction (MI) (Nikpay et al., 2015), T2D (Scott et al., 2017), rheumatoid arthritis (RA) (The Wellcome Trust Case Control Consortium, 2007), type 1 diabetes (T1D) (The Wellcome Trust Case Control Consortium, 2007), hypertension (The Wellcome Trust Case Control Consortium, 2007), ankylosing spondylitis (AS) (Burton et al., 2007), ischemic stroke (IS) (Malik et al., 2018), and multiple sclerosis (MS) (Burton et al., 2007). In addition to these diseases, we also explored 57 adult diseases from the UK Biobank (Sudlow et al., 2015) (**Text S1**). However, because the UK Biobank data set contains only a small number of cases for most of these diseases, we will only briefly mention our results on UK Biobank. Finally, to identify complex traits that may mediate the causal effect of birth weight on any identified adult disease, we obtained a GWAS summary statistics for 38 complex traits in adulthood (**Text S2**). These traits include educational attainment (i.e., EduYears and College) (Rietveld et al., 2013), smoking behaviors (The Tobacco and Genetics Consortium, 2010), early growth traits (Cousminer et al., 2013), blood lipid traits (Teslovich et al., 2010), glycemic and harmonic traits (Dupuis et al., 2010), and blood pressures (Richey Sharrett).

### Selecting Instruments for Mendelian Randomization Analyses

We first selected 47 independent index SNPs (**Table 1**) to serve as valid instrumental variables for birth weight based on the EGG consortium study (Horikoshi et al., 2016) using plink (version v1.90b3.38) (Purcell et al., 2007) following previous work (Noyce et al., 2017) (**Text S3**). Next, for each disease in turn, we relied on the corresponding disease GWAS and extracted summary statistics on the disease for the 47 index SNPs of birth weight. For these index SNPs that do not have summary statistics in the corresponding disease, we either replaced them with proxy SNPs that are in high linkage disequilibrium (LD) with the index SNPs or imputed the summary statistics (Pasaniuc et al., 2014) for the index SNPs—both approaches yield similar results (**Text S3**). While our main MR analyses were performed using the above 47 SNPs as instrumental variables, to examine the robustness of the results, we also performed an alternative MR analysis using a slightly different set of 48 SNPs. These 48 SNPs are presented in the original GWAS of birth weight (Horikoshi et al., 2016) and that are also independent SNPs showing strong association with birth weight (*p* < 5.00E−8) (**Table S2**). For each disease, we also examined another alternative set of birth weight instruments by following (Østergaard et al., 2015) to exclude potentially pleiotropic SNPs, which show relatively strong associations with the given disease/trait (*p* < 1.06E−3, which is 0.05/47) (**Text S3**) (Lawlor et al., 2008; Sheehan et al., 2008). In this alternative analysis, the number of instruments excluded varies for different diseases and ranges from 1 (e.g., for AMD) to 18 (e.g., for height) (**Table S3**). We further performed another alternative analysis by removing instruments with possible pleiotropic effects with any disease/outcome (*p* < 5.00E−8) recorded in the Phenoscanner (Staley et al., 2016) and NHGRI-EBI Catalog (MacArthur et al., 2017) databases. In this analysis, only a final set of 21 instruments were kept for birth weight. Additionally, seven independently associated SNPs yielded from Horikoshi et al. (2013) (**Table S4**) are also employed to be instruments for further validation.

**Table 1:** Summary information for the 47 autosomal SNPs that are used as instrumental variables in the MR analysis.

Chr	SNP	Position	Gene	Allele	MAF	BETA	SE	*p*	*N*	PVE	*F*
7	rs138715366	44,246,271	*YKT6-GCK*	T/C	0.01	−0.2412	0.0229	7.20E−26	132,343	8.38E−04	110.99
17	rs144843919	29,037,339	*SUZ12P1-CRLF3*	A/G	0.04	−0.0660	0.0116	1.40E−08	121,357	2.67E−04	32.41
3	rs900399	156,798,732	*CCNL1-LEKR1*	G/A	0.39	−0.0523	0.0039	2.20E−41	143,663	1.25E−03	179.80
22	rs41311445	42,070,374	*SREBF2*	C/A	0.10	−0.0445	0.0066	1.60E−11	135,729	3.35E−04	45.48
6	rs35261542	20,675,792	*CDKAL1*	A/C	0.27	−0.0444	0.0041	4.40E−27	143,667	8.16E−04	117.33
2	rs7575873	23,962,647	*ATAD2B*	G/A	0.12	−0.0384	0.0057	1.20E−11	139,425	3.25E−04	45.33
6	rs10872678	152,039,964	*ESR1*	C/T	0.28	−0.0375	0.0041	6.90E−20	143,672	5.82E−04	83.66
21	rs2229742	16,339,172	*NRIP1*	C/G	0.13	−0.0360	0.0060	2.20E−09	143,672	2.51E−04	36.07
4	rs4144829	17,903,654	*LCORL*	T/C	0.73	−0.0341	0.0042	5.30E−16	139,426	4.73E−04	65.98
8	rs13266210	41,533,514	*ANK1-NKX6-3*	G/A	0.21	−0.0308	0.0045	1.30E−11	139,429	3.36E−04	46.86
6	rs1187118	34,169,020	*HMGA1*	T/A	0.83	−0.0299	0.0051	3.60E−09	137,043	2.51E−04	34.41
10	rs2497304	94,492,716	*HHEX-IDE*	T/C	0.48	−0.0282	0.0037	2.60E−14	143,673	4.04E−04	58.07
9	rs7854962	96,900,505	*PTCH1*	G/C	0.22	−0.0279	0.0046	1.90E−09	139,424	2.64E−04	36.82
5	rs854037	57,091,783	*5q11.2*	G/A	0.19	−0.0268	0.0048	2.20E−08	139,429	2.24E−04	31.24
7	rs11765649	23,479,013	*IGF2BP3*	C/T	0.25	−0.0267	0.0043	5.80E−10	139,428	2.76E−04	38.49
20	rs28530618	31,275,581	*C20orl203*	G/A	0.51	−0.0261	0.0038	7.70E−12	138,162	3.41E−04	47.13
15	rs7402982	99,193,269	*IGF1R*	G/A	0.57	−0.0232	0.0039	2.30E−09	139,423	2.54E−04	35.42
8	rs12543725	142,247,979	*SLC45A4*	A/G	0.41	−0.0231	0.0038	1.20E−09	139,431	2.65E−04	36.96
7	rs798498	2,795,882	*GNA12*	G/T	0.31	−0.0229	0.0040	1.30E−08	139,427	2.35E−04	32.77
3	rs2168443	46,947,087	*PTH1R*	A/T	0.62	−0.0228	0.0039	3.50E−09	139,426	2.45E−04	34.17
15	rs12906125	91,427,612	*FES*	A/G	0.32	−0.0228	0.0040	1.70E−08	141,281	2.30E−04	32.50
22	rs134594	29,468,456	*KREMEN1*	T/C	0.65	−0.0227	0.0040	1.00E−08	137,340	2.34E−04	32.14
13	rs7998537	40,662,742	*LINC00332*	A/G	0.32	−0.0222	0.0040	3.90E−08	139,429	2.21E−04	30.82
3	rs10935733	148,622,968	*CPA3*	C/T	0.59	−0.0221	0.0039	9.20E−09	139,426	2.30E−04	32.07
12	rs2306547	26,877,885	*ITPR2*	T/C	0.46	−0.0211	0.0037	1.80E−08	139,432	2.33E−04	32.49
9	rs1411424	113,892,963	*LPAR1*	A/G	0.52	0.0212	0.0038	2.20E−08	139,428	2.23E−04	31.10
6	rs9368777	33,788,637	*HMGA1*	C/G	0.58	0.0215	0.0038	2.20E−08	135,709	2.36E−04	32.03
17	rs72833480	45,964,861	*SP6-SP2*	A/G	0.29	0.0226	0.0041	4.60E−08	139,426	2.18E−04	30.40
20	rs6040076	10,658,882	*JAG1*	C/G	0.49	0.0231	0.0039	2.00E−09	139,424	2.52E−04	35.14
16	rs28415607	19,993,015	*GPR139*	C/T	0.25	0.0233	0.0043	5.00E−08	143,660	2.04E−04	29.31
20	rs6016377	39,172,728	*MAFB*	T/C	0.43	0.0239	0.0039	9.50E−10	139,425	2.69E−04	37.51
5	rs2946179	157,886,627	*EBF1*	C/T	0.73	0.0240	0.0042	1.30E−08	143,666	2.27E−04	32.62
4	rs2131354	145,599,908	*HHIP*	A/G	0.53	0.0259	0.0037	4.10E−12	139,431	3.51E−04	48.96
1	rs3753639	154,986,091	*ZBTB7B*	C/T	0.24	0.0306	0.0045	7.30E−12	138,162	3.35E−04	46.30
17	rs113086489	7,171,356	*CLDN7*	T/C	0.56	0.0307	0.0038	9.10E−16	139,426	4.68E−04	65.28
1	rs72480273	161,644,871	*FCGR2B*	C/A	0.17	0.0313	0.0051	8.00E−10	138,380	2.72E−04	37.65
1	rs2473248	22,536,643	*WNT4-ZBTB40*	C/T	0.87	0.0325	0.0057	1.00E−08	139,428	2.33E−04	32.49
13	rs1819436	78,580,283	*RNF219-AS1*	C/T	0.87	0.0329	0.0057	6.30E−09	138,979	2.40E−04	33.36
9	rs10818797	126,020,405	*STRBP*	C/T	0.14	0.0345	0.0054	1.20E−10	139,427	2.93E−04	40.86
10	rs740746	115,792,787	*ADRB1*	A/G	0.73	0.0364	0.0042	3.80E−18	143,672	5.23E−04	75.18
10	rs79237883	104,940,946	*NT5C2*	C/T	0.08	0.0371	0.0067	3.50E−08	143,666	2.13E−04	30.61
12	rs7964361	102,994,878	*IGF1*	A/G	0.09	0.0391	0.0067	4.70E−09	139,428	2.44E−04	34.03
3	rs11719201	123,068,744	*ADCY5*	T/C	0.23	0.0463	0.0044	2.40E−26	143,670	7.70E−04	110.71
2	rs17034876	46,484,310	*EPAS1*	T/C	0.70	0.0471	0.0042	2.60E−29	134,460	9.34E−04	125.70
11	rs72851023	2,130,620	*INS-IGF2*	T/C	0.07	0.0476	0.0075	2.90E−10	135,776	2.97E−04	40.34
7	rs111778406	72,957,570	*MLXIPL*	G/A	0.07	0.0492	0.0075	5.80E−11	140,932	3.05E−04	43.00
9	rs3780573	98,239,503	*PTCH1*	A/G	0.10	0.0555	0.0064	7.00E−18	134,750	5.58E−04	75.23

These SNPs are associated with birth weight at the genome-wide significance level (p < 5.00E−08) in a meta-analysis with up to 143,677 individuals of European ancestry. SNPs are ordered based on their effect size estimates. All the genes (fourth column) were reported to be associated with birth weight in previous GWASs (Horikoshi et al., 2013; Horikoshi et al., 2016). Chr, chromosome; SNP, single-nucleotide polymorphism id; Position, genome position in base pair; Allele, effect allele and alternative allele; MAF, minor allele frequency; BETA, SNP effect size; SE, standard error; PVE, proportion of variance in birth weight explained by the SNP; p, N, and F represent p value, sample size, and F statistic, respectively.

### Mendelian Randomization, Sensitivity Analyses, and Multivariable Analyses

With the above data, we performed MR analyses. Details of MR are described in **Text S3**. Briefly, for each SNP in turn, we first estimated the proportion of phenotypic variance explained (PVE) by the SNP using summary statistics (Shim et al., 2015) and computed *F* statistic to ensure strong instrument (Cragg and Donald, 1993; Burgess et al., 2017a). We then performed MR analyses using the random-effects version of the inverse-variance weighted (IVW) methods (Burgess et al., 2017a) to estimate and test the causal effects of birth weight on each of the 21 adult diseases. During the process, we employed Q and I^2^ statistics to measure causal effect size heterogeneity across instruments (Thompson and Sharp, 1999) and performed power calculation using analytic forms (Brion et al., 2013; Freeman et al., 2013; Burgess, 2014).

For each disease identified to be causally affected by birth weight in the MR analyses, we performed a series of sensitivity analyses to ensure results robustness. Specifically, we performed a median-based MR analysis to guard against outlying instruments (Bowden et al., 2016a). We conducted a leave-one-out (LOO) cross-validation analysis (Noyce et al., 2017) and Mendelian Randomization Pleiotropy RESidual Sum and Outlier (MR-PRESSO) analysis (Verbanck et al., 2018) to directly examine potential instrument outliers. We carried out MR-Egger regression to examine the assumption of directional pleiotropic effects (Bowden et al., 2016b; Burgess and Thompson, 2017). To examine the potential influence of maternal genetic effects (Horikoshi et al., 2016), we performed an additional sensitivity analysis by excluding SNPs that affect birth weight through maternal effects (Lawlor et al., 2017; Beaumont et al., 2018; Zanetti et al., 2018). We attempted to directly control for maternal effects (**Table S5**) in the analysis of birth weight effect on diseases using a genetic-score based approach. We also carried out bias calculations and used simulations to examine the impact of maternal effects on causal effect estimations (Vanderweele and Arah, 2011; Lawlor et al., 2017) (**Text S4**). We performed reverse causal inference to examine the possible reverse causality from diseases to birth weight. We also applied a recently developed analysis method iMAP (Zeng et al., 2018) to jointly model all genome-wide SNPs to provide supportive evidence on the directionality of the causal relationship between birth weight and these identified diseases.

Finally, we investigated whether any of the 38 complex traits may mediate the causal effect of birth weight on the identified adult diseases. To do so, we first performed MR analysis to examine whether birth weight causally affect any of the 38 complex traits. In particular, for each of the 38 traits in turn, we extracted summary statistics from the corresponding GWAS for the 47 instrumental variables of birth weight. We replaced missing SNPs with proxy ones when necessary and applied the IVW methods following the same procedure as described above. Next, we performed a multivariable MR analysis (Do et al., 2013; Burgess and Thompson, 2015; Burgess et al., 2017b) for each pair of identified trait and disease to investigate whether any of these complex traits may mediate the causal effect of birth weight on the identified disease. The multivariable MR analysis allows us to estimate and test both the direct effect of birth weight on the disease and the indirect effect of birth weight on the disease through the complex trait (Burgess et al., 2017b).

Note that, for some sensitivity analyses above (e.g., MR-EGGER and IMAP), we mostly followed the practical recommendations of other Mendelian randomization and performed them only when a significant causal association between birth weight and an adult disease (e.g., CAD) was detected.

## Results

### Mendelian Randomization Identifies Three Adult Diseases That Are Causally Affected by Birth Weight

We first selected a set of 47 SNPs from a large-scale GWAS for birth weight based on 143,677 individuals to serve as instrumental variables for birth weight (**Table 1** and **Figure S1**). We examined the strength of these instruments using *F* statistic based on the EGG GWAS discovery sample of birth weight following (Noyce et al., 2017) (**Text S3**). For the 47 instrumental variables, their *F* statistics individually range from 29.36 to 179.83 (**Table 1**) with an overall *F* statistic of 49.22 for all 47 instruments. These values are all above the usual threshold of 10, suggesting that the selected genetic variants have sufficiently strong effect sizes to be used as instrumental variables and that weak instrument bias is unlikely to occur in our analysis.

**Figure 2:**
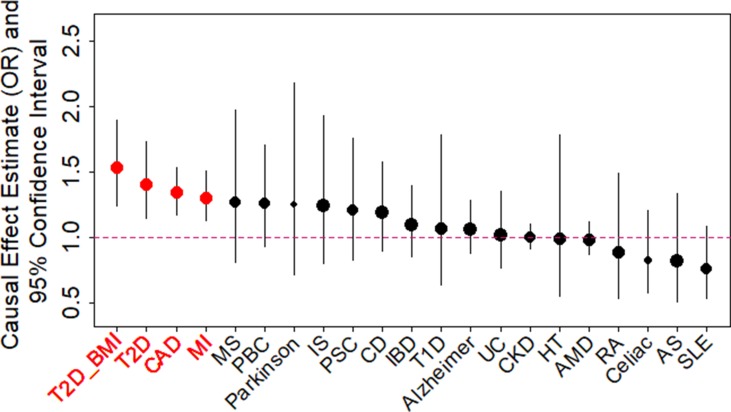
Causal effect estimates and 95% confidence intervals for lower birth weight on 21 diseases using the random-effects inverse variance weighted (IVW) method. Diseases are ordered based on their causal effect estimates. Estimations are carried out using both index SNPs and proxy SNPs. The dot size is proportional to the number of instrumental variables used for the given disease while dot color represents significance (*p* < 0.05 are highlighted in red). Disease names (x-axis) are further highlighted in red if the causal effects are significant after Bonferroni correction (*p* < 0.05/21).

We examined the causal relationship between birth weight and 21 adult diseases through MR analysis using the selected instruments. Across 21 diseases, we displayed the causal effects of *lower* birth weight instead of birth weight in all figures and tables throughout the text by supplying a negative sign on the estimated birth weight effect (**Figure 2** and **Table S6**). We found that lower birth weight is causally associated with increased risks for three diseases after Bonferroni correction (i.e., *p*-value threshold of 0.05/21 = 2.38E−3). These three diseases include CAD (Nikpay et al., 2015), MI (Nikpay et al., 2015), and T2D (both in terms of the original T2D status and in terms of T2D_BMI, which represents the T2D status after adjusting for BMI) (Scott et al., 2017). Because we identified effect size heterogeneity across the 47 instruments (**Table S6**; *p* values based on Q statistic are 7.41E−1, 1.42E−2, 1.40E−4, and 1.92E−2, and the I^2^ statistics are 0%, 33.1%, 48.9%, and 31.8% for CAD, MI, T2D, and T2D_BMI, respectively), we choose to mainly present our results from the random-effects IVW analysis, which properly accounts for causal effect heterogeneity.

For each of the three diseases, we compute the odds ratio (OR) of the disease for one unit decrease of birth weight, where the unit is defined as the standard deviation of birth weight, estimated to be 488 g across 35 studies in the original meta-analysis (**Table S1**) (Horikoshi et al., 2016). We found that a unit lower birth weight is causally associated with an increased CAD risk with an estimated OR of 1.34 [95% confidence interval (CI) 1.17–1.53, *p* = 1.54E−5]; a unit lower birth weight is causally associated with an increased MI risk with an estimated OR of 1.30 (95% CI 1.13–1.51, *p* = 3.31E−4); a unit lower birth weight is also causally associated with an increased T2D risk, with an estimated OR of 1.41 (95% CI 1.15–1.73, *p* = 1.11E−3) for the original T2D, and with an estimated OR of 1.54 (95% CI 1.25–1.89, *p* = 6.07E−5) for the BMI-adjusted T2D (i.e., T2D_BMI). These causal associations are also supported by the results generated using the smaller set of instruments from Horikoshi et al. (2013) (**Table S6**).

Consistent with the fetal origins of adult diseases hypothesis, the causal effects of lower birth weight on most of the diseases investigated are estimated to be positive [14 out of 21 (66.7%), **Table S6**], though most of these estimates are not statistically significantly different from one. In addition, the estimated causal effects of lower birth weight on four diseases (MS, Parkinson’s disease, PBC, and PSC) in addition to the three diseases mentioned in the above paragraph are above OR of 1.2, though these estimates came with large standard errors. Power calculation results based on parameters estimated in the MR analysis also suggest that the nonsignificant results for the remaining diseases may be due to a lack of statistical power (**Table S6**). The lack of power for the remain diseases suggest that a lack of association between birth weight and these diseases should not be over-interpreted and that larger sample sizes are needed to elucidate the causal effects of birth weight on these diseases.

Finally, besides these 21 diseases, we have also attempted to examine additional 57 adult diseases from the UK Biobank (**Table S7**). Unfortunately, due to the extremely low number of cases (e.g., only 119 cases of Alzheimer’s disease) for most diseases there and the resulting low statistical power (Zhou et al., 2018), we did not detect any statistically significant associations between birth weight and those diseases in the UK Biobank (**Table S7**).

### Mendelian Randomization Results Are Robust With Respect to Instrument Outliers and the Choice of Instrumental Variables

**Figure 3:**
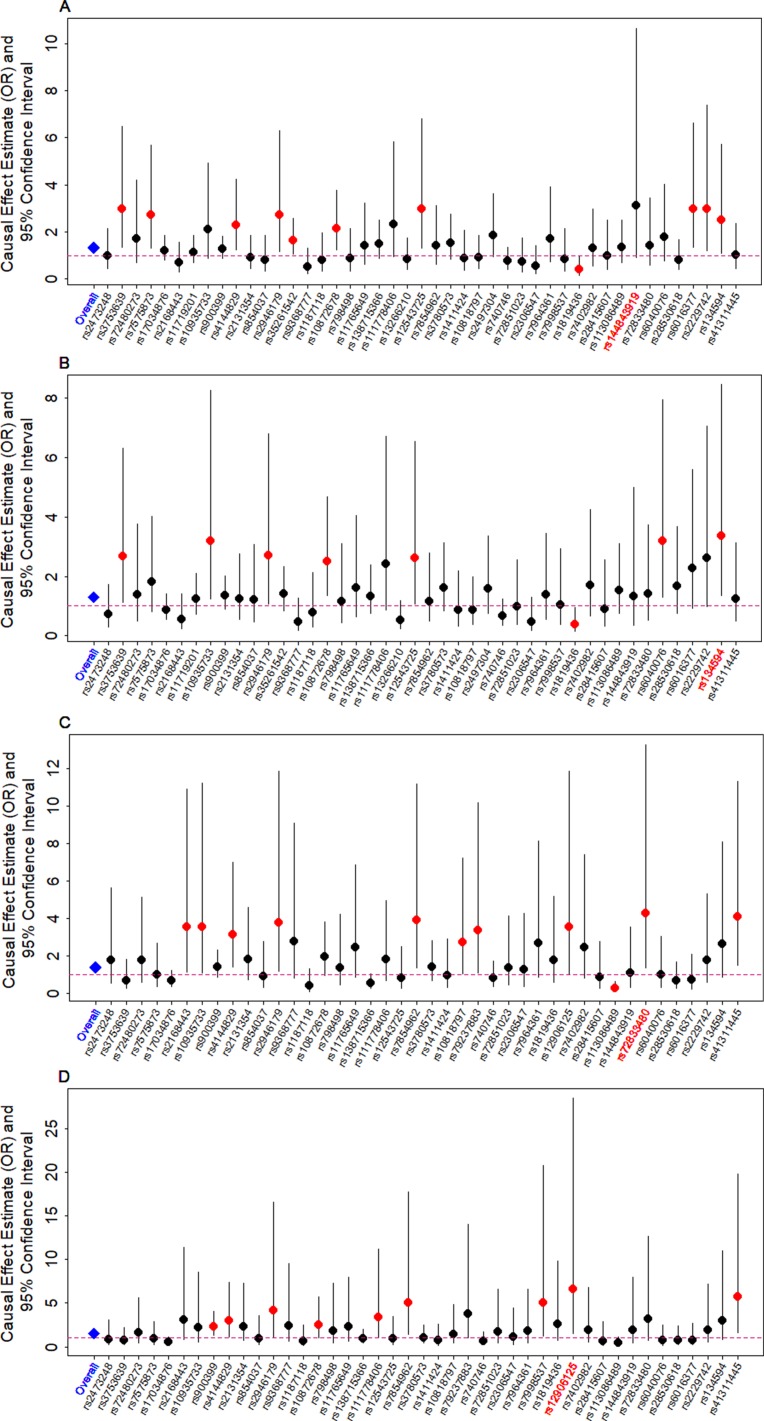
Causal effect estimates and 95% confidence intervals for lower birth weight on **(A)** CAD, **(B)** MI, **(C)** T2D, and **(D)** T2D_BMI. Estimations are carried out either using all SNPs (first column on x-axis) or using individual SNPs (the remaining columns on x-axis) based on Equation (14) in **Text S3**. Dot size is proportional to the effect size estimates, while dot color represents significance (*p* < 0.05 are highlighted in red). SNP that yields the largest causal effect estimate is also highlighted in red (x-axis).

**Figure 4:**
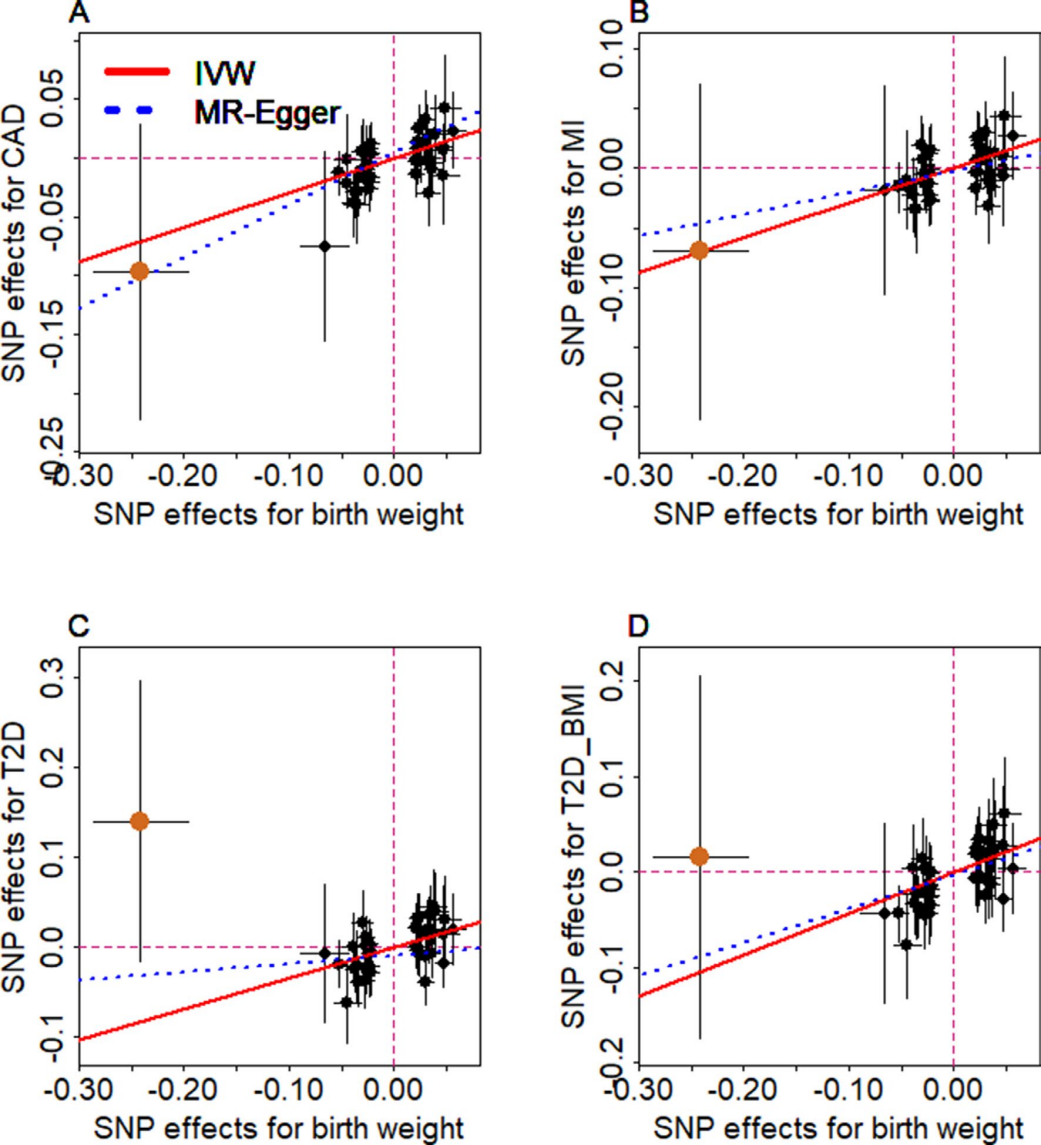
Relationship between the effect size estimates on lower birth weight (x-axis) and the effect size estimates on diseases (y-axis) for the 47 SNPs that serve as instrumental variables. Examined diseases include **(A)** CAD, **(B)** MI, **(C)** T2D, and **(D)** T2D_BMI. 95% confidence intervals for the estimated SNP effect sizes on disease are shown as vertical black lines, while the 95% confidence intervals for the estimated SNP effect sizes on birth weight are shown as horizontal black lines. The vertical and horizontal red dotted lines represent zero effects. The slope of fitted lines represents the estimated the casual effects of birth weight on the corresponding disease obtained using either the random-effects IVW method (red solid lines) or the MR-Egger regression (blue dotted lines). SNP outlier rs13875366 (chocolate dot) was not included in MR-Egger regression to avoid outlier influence. Due to the inclusion of an intercept in the MR-Egger regression, the fitted lines by MR-Egger regression (blue dotted lines) do not necessarily pass the origin.

We examine the causal relationship between birth weight and the three diseases (CAD, MI, T2D, and T2D_BMI) in details here. We first display the causal effects of lower birth weight for each of the three diseases estimated using individual instrumental variables in **Figure 3**. We also plot the SNP effect sizes on birth weight versus the effect sizes on these diseases in **Figure 4**. One SNP, rs138715366, appears to be an outlier for all these traits. rs138715366 has a low minor allele frequency (MAF = 0.89%), is located within the intronic regions of the gene *YKT6-GCK* on Chr 7, and has the largest effect size on birth weight among all instrument variables (= −0.24; with 95% CI −0.20 to −0.29, *p* = 7.20E−26; **Figure S1** and **Table 1**). In addition, another SNP, rs144843919, also appears to be a potential outlier for CAD. The effect size of rs144843919 on birth weight is estimated to be −0.066 (95% CI −0.09 to −0.04, *p* = 1.40E−8). However, as we will show in the next paragraph, neither SNP has substantial influence on the estimation of the causal effects.

Next, we directly tested whether any instrument is an outlier for any of the four diseases using MR-PRESSO. The results show that no significant instrument outliers exist for the MR analysis of each of the three diseases (CAD, MI, T2D, or T2D_BMI) at the nominal significance level of 0.05. In addition, we performed LOO analyses, which are also stable and demonstrate that no single instrumental variable substantially influences the estimation of the casual effects of birth weight on the four traits (**Figure S2**). For example, after removing rs138715366, the ORs for a unit decrease of birth weight are estimated to be 1.34 (95% CI 1.20–1.49, *p* = 9.70E−8) for CAD, 1.30 (95% CI 1.15–1.47, *p* = 1.66E−5) for MI, 1.48 (95% CI 1.27–1.71, *p* = 2.66E−7) for T2D, and 1.57 (95% CI 1.32–1.87, *p* = 3.95E−7) for T2D_BMI, almost identical to the ORs estimated using all these instrumental variables together (**Figure 2**).

Our primary results described in the previous section are based on using 47 instrumental variables. For certain diseases, summary statistics for some of the 47 index SNPs are unavailable. In these cases, we have used proxy SNPs that are in high LD using a certain correlation threshold. We found that our results are robust with respect to various correlation thresholds to obtain these proxy SNPs (**Figure S3**). Besides using proxy SNPs, we imputed summary statistics for the unavailable index SNPs and performed analysis using all index SNPs. Results with imputed summary statistics remain similar (**Figure S4**). We also performed analysis using only part of the 47 index SNPs that are available for the given disease, without using any proxy SNPs or imputation; we again obtained consistent results (**Figure S5**). Besides the analysis using a set of 47 instruments, we performed an alternative analysis by using another set of 48 instrumental variables (**Table S2**) that are presented in the original meta-analysis study (Horikoshi et al., 2016). Again, the results are largely similar to those in our main analyses (**Figures S6** and **S7**).

### Various Sensitivity Analyses Further Validate the Main Mendelian Randomization Results

We performed sensitivity analyses to complement our main MR analysis results obtained with IVW. First, to guard against the possibility that some instruments are invalid, we conducted a MR analysis using the weighted median method (Bowden et al., 2016a) for CAD, MI, T2D, and T2D_BMI. The weighted median estimate approach yields qualitatively similar results as our main analysis (**Text S3**), suggesting that invalid instruments unlikely bias our main results.

To guard against the possibility that the used instruments may display horizontal pleiotropy and thus bias causal effect estimation, we performed the MR-Egger regression (Bowden et al., 2016b; Burgess and Thompson, 2017) for the four traits. The results from the MR-Egger regression analysis are again consistent with our main results (**Text S3** and **Figure 2**). In addition, none of the intercepts from MR-Egger regression are significantly deviated from zero: they are estimated to be 0.005 (95% CI −0.010 to 0.020, *p* = 0.515) for CAD, −0.003 (95% CI −0.019 to 0.013, *p* = 0.756) for MI, −0.010 (95% CI −0.031 to 0.012, *p* = 0.383) for T2D, and −0.003 (95% CI −0.026 to 0.019, *p* = 0.783) for T2D_BMI, respectively. Moreover, funnel plots also display symmetric pattern of effect size variation around the point estimate (**Figure S8**). Together, MR-Egger regression results and funnel plots suggest that horizontal pleiotropy unlikely bias our results. Furthermore, using the set of instruments that were left after excluding all possible pleiotropic instruments based on the Phenoscanner (Staley et al., 2016) and NHGRI-EBI Catalog (MacArthur et al., 2017) datasets, we can still obtain similar results. For example, the ORs of birth weight on CAD, MI, T2D, and T2D_BMI are estimated to be 1.26 (95% CI 1.04–1.54, *p* = 1.98E−2), 1.16 (95% CI 0.93–1.50, *p* = 0.176), 1.78 (95% CI 1.32–2.40, *p* = 1.66E−4), and 1.83 (95% CI 1.30–2.56, *p* = 5.14E−4), respectively.

One of the main difficulties in causal inference is to distinguish causality from reverse causality (Burgess et al., 2017a). Because of the time order and the fact that birth weight precedes adult diseases, the issue of reverse causation is unlikely a concern in our study. Nevertheless, to guard against the small possibility that our results are driven by reverse causality, we performed IVW analysis in the reverse direction to examine the causal effects of CAD, MI, T2D, or T2D_BMI on birth weight. Results show that there are no reverse causal associations between any of the four traits and birth weight as one would expect (**Table S8** and **Figure S10**).

To complement the MR analysis, we also performed analysis using the recently developed iMAP method (Zeng et al., 2018). iMAP analyzes a pair of traits jointly and borrows information across all genome-wide SNPs to provide additional evidence regarding the causal relationship between the two traits. In particular, iMAP estimates the proportion of SNPs associated with one trait that is also associated with the other. By estimating such proportions, iMAP has the potential to provide evidence supporting potentially directional causality between the two analyzed traits (Zeng et al., 2018). Here, we applied iMAP to analyze birth weight and each of the four traits at a time. We estimated the proportion of SNPs associated with birth weight that is also associated with CAD, MI, T2D, and T2D_BMI to be 0.125, 0.134, 0.452, and 0.472, respectively. In contrast, the proportion of SNPs associated with CAD, MI, T2D, and T2D_BMI that is also associated with birth weight are only 0.053, 0.029, 0.211, and 0.130, respectively. The asymmetrical probabilities estimated from iMAP suggest that SNPs associated with the birth weight are also more likely associated with the disease than the other way around. Therefore, iMAP provides additional genome-wide evidence supporting the causal effects of birth weight on the identified diseases.

### Causal Effects of Birth Weight on the Three Identified Diseases Are Not Mediated Through Other Complex Traits

We explored the causal pathways through which birth weight may causally affect the adult diseases. To do so, we obtained 35 quantitative traits and 3 binary traits that may mediate the causal effects of birth weight onto diseases (**Text S2**). For each trait in turn, we estimated the casual effect of birth weight on the trait using all available instruments using IVW (**Figure S11** and **Table S9**). Among all examined traits, we only identified adult weight to be causally affected by birth weight based on the Bonferroni adjusted significance threshold (*p* < 0.05/38). In particular, birth weight is positively associated with adult weight with the causal effect estimated to be 0.36 in the random-effects IVW analysis (95% CI 0.12–0.60, *p* = 3.18E−3).

The lack of significant causal effects of birth weight on most examined complex traits are consistent with the lack of significant causal effects of birth weight on some of the examined diseases described in the earlier section. For example, with currently available GWAS gene data sets for us, owing to lack of power resulting from small sample sizes (∼9,000), in our analysis we cannot find evidence that supports the causal association between birth weight with both systolic blood pressure (SBP) and diastolic blood pressure (DBP) (estimated causal effect on SBP is 0.35, 95% CI −0.10 to 0.79, *p* = 0.127; estimated causal effect on DBP is 0.27, 95% CI −0.17 to 0.71, *p* = 0.233) by random-effects IVW analysis. The lack of causal association between birth weight and blood pressure is consistent with our earlier result on a lack of detectable causal association between birth weight and hypertension. In addition, the lack of causal association between birth weight and many complex traits suggests that the causal effects of birth weight on CAD, MI, or T2D are unlikely to be mediated by blood pressures or many other complex traits, which is further confirmed by the following multivariable regression.

To examine the possibility that some complex traits (e.g., adult weight, BMI, blood pressures, blood lipids, or hypertension) may mediate the causal effect of birth weight on each of the four traits (i.e., CAD, MI, T2D, and T2D_BMI), we performed a comprehensive multivariable MR analysis for all the 38 complex traits (**Text S2**) (Burgess et al., 2017b). The results do not provide evidence supporting the mediator role of those complex traits in the relationship between birth weight and the three diseases including CAD, MI, and T2D (**Table S10**). For example, the estimated direct effect for a unit lower birth weight for CAD, MI, T2D, and T2D_BMI are 1.28 (95% CI 1.09–1.50, *p* = 4.57E−3), 1.28 (95% CI 1.07–1.52, *p* = 8.48E−3), 1.66 (95% CI 1.17–2.37, *p* = 6.84E−3), and 1.73 (95% CI 1.19–2.51, *p* = 6.28E−3). The estimated indirect effect of birth weight on CAD, MI, T2D, and T2D_BMI are 1.05 (95% CI 0.85–1.30, *p* = 0.653), 1.02 (95% CI 0.84–1.23, *p* = 0.852), 0.85 (95% CI 0.56–1.28, *p* = 0.432), and 0.89 (95% CI 0.58–1.37, *p* = 0.606), respectively. Therefore, the lack of detectable indirect effect suggests that either adult weight unlikely mediate the causal effect of birth weight on any of the three diseases or that we do not have sufficient power to detect such mediation effect.

### Special Sensitivity Analyses to Examine the Influence of Maternal Effects on the Main Mendelian Randomization Results

Finally, we performed additional sensitivity analyses to examine the influence of maternal effects on MR results (**Text S4**). First, we excluded among the set of 47 instruments those instruments that may potentially exhibit maternal effects on birth weights relying on a recent GWAS of maternal effects on birth weights (Beaumont et al., 2018). We deleted a total of 10 instruments and with the remaining 37 instruments, we estimated the ORs (again, after removing the potentially pleiotropic instruments as done above) for a unit decrease in birth weight to be 1.37 (95% CI 1.17–1.61, *p* = 8.18E−5) for CAD, 1.31 (95% CI 1.10–1.55, *p* = 1.90E−3) for MI, 1.42 (95% CI 1.11–1.80, *p* = 4.62E−3) for T2D, and 1.41 (95% CI 1.10–1.80, *p* = 6.35E−3) for T2D_BMI, respectively. The results are consistent with the main results, suggesting that maternal effects unlikely bias our estimates.

Second, we directly controlled for maternal effects in the analysis of birth weight on T2D using a genetic-score based approach. Specially, we first obtained two sets of birth weight instruments: one set contains instruments for offspring’s effect on birth weight (Horikoshi et al., 2016) (i.e., 47 index SNPs in **Table 1**) and another set contains for instruments for mother’s effect on birth weight (i.e., 10 index SNPs in **Table S5**). We constructed two genetic scores using these instruments in the Genetic Epidemiology Research on Aging (GERA) cohort (Banda et al., 2015): one genetic score is obtained using the offspring’s instruments and represents offspring effect, while another genetic score is obtained using mother’s instruments and represents maternal effect. Afterwards, we examined the relationship between offspring’s birth weight and adult T2D by fitting a logistic model, treating T2D as outcome and offspring’s genetic score as predictor, while controlling for mother’s genetic score and other covariates. The adjusted OR for lower birth weight on T2D is estimated to be 1.07 (95% CI 1.04–1.11, *p* = 3.91E−6), again supporting our observation that the lower birth weight is a risk factor of adult diseases. We can only apply this analysis to T2D because we only have individual-level data for T2D in the GERA cohort.

Third, we performed simulations to evaluate the extent to which the maternal effects may influence the birth-weight causal effect estimation in MR (**Figure S12**) (Lawlor et al., 2017). The simulation results show that the causal effects of birth weight are indeed approximately unbiased when the maternal effect is in a reasonable range (**Figure S13**) (Horikoshi et al., 2016; Beaumont et al., 2018) (e.g., each instrument has a maternal effect that explains 0.1% or 0.01% of phenotypic variance in birth weight). Only when the maternal effect is unrealistically strong (e.g., each instrument explains 1% or 10% of phenotypic variance in birth weight due to maternal effects), then the causal effect estimates can be slightly biased upward. The approximate unbiasedness results in simulations suggest that our main MR results are unlikely biased by realistic maternal effects.

Finally, we performed direct calculation to estimate bias due to maternal effect confounding with realistic assumption. Intuitively, if we know what the maternal effect (denoted by ϑ) is and if we know the probability that a mother gives birth to an offspring with low birth weight (denoted by π), then, even if we do not observe the maternal alleles, we can still compute the estimation bias induced by maternal effect (**Text S4**). As an extreme example, if we set ϑ to be as high as 0.30 and set π to be either 6.9% (which represents the corresponding estimate in developed countries (Johnson et al., 2017)) or 28% (which represents corresponding estimate in developing countries (WHO, 2014)), then the estimation bias due to maternal effects is expected to be only 7.87% or 2.16%, respectively. Therefore, potential estimation bias due to maternal effects is expected to be small under realistic assumptions.

## Discussion

### A Summary of Our Mendelian Randomization Analyses

We have investigated the fetal origins of adult diseases hypothesis by performing a series of comprehensive MR analyses to examine the causal effects of birth weight on 21 adult diseases and 38 other complex traits. Our study relies on summary statistics obtained from 37 GWASs with sample sizes ranging from 4,798 (for RA, The Wellcome Trust Case Control Consortium, 2007) to 339,224 (for BMI, Locke et al., 2015), thus representing one of the largest and most comprehensive MR analyses performed on birth weight to date. The large sample size used in our study allows us to fully establish an inverse causal relationship between birth weight and three adult diseases that include CAD, MI, and T2D. These inferred causal relationships are robust with respect to the selection of instrumental variables and to the choice of statistical methods, and are carefully validated in the present study through various sensitive analyses. In addition, our analysis also suggests that the lack of causality evidence between birth weight and the other diseases may be partly due to a lack of statistical power resulting from relatively small sample sizes for the remaining diseases. Finally, we investigate the possibility that any of the analyzed 38 complex traits may mediate the causal effects of birth weight on CAD, MI, or T2D. Overall, our study provides important causality evidence supporting the fetal origins hypothesis for three adult diseases and suggests that increasing sample size is likely needed to reveal causal effects of birth weight for the other disorders.

The mechanisms underlying the causal associations between low birth weight and adult diseases (e.g., CAD or T2D) are not fully understood. Many previous studies have suggested that a lot of risk factors may mediate the causal effect of birth weight on adult diseases. For example, it has been shown that insulin resistance is likely a mediator of low birth weight effect on T2D (Dabelea et al., 1999). As another example, rapid weight gain during childhood following the low birth weight has been shown to be associated with increased risk of obesity and CAD (Andersen et al., 2010). Therefore, while we did not detect statistically significant traits that may mediate the effects of birth weight on adult diseases in the present study, we acknowledge that we were only able to analyze 38 complex traits that likely represent only a small proportion of possible mediators and that our analysis on these complex traits may also lack power due to the relatively small sample sizes for these traits.

### Comparison of Our Findings With Those in Previous Studies

The finding of the identified causal association between birth weight and CAD in our study is consistent with an early twin study (Oberg et al., 2011). This early twin study shows that there is statistically significant association between CAD risk difference and birth weight difference in dizygotic twin pairs, but there is no statistically significant association between the two in monozygotic twin pairs. Intuitively, if birth weight effect on CAD is indeed causal, then the genetic component of birth weight would be associated with the risk of CAD. Because monozygotic twins share the identical genetic component, genetics does not contribute to the birth weight *difference* between monozygotic twins. Subsequently, if birth weight is indeed causally associated with CAD, then one would expect no association between birth weight difference and CAD risk difference in monozygotic twins. In contrast, because dizygotic twins do not share identical genetic component, the genetic component underlying birth weight difference between dizygotic twins becomes a good indicator of CAD risk differences between dizygotic twins. From this aspect, the results from the previous twin study are consistent with the causal relationship between birth weight and CAD identified in the present study.

Our findings are complementary to and consistent with the previous observation study that showed an inverse genetic correlation between birth weight and adult diseases (Horikoshi et al., 2016). Our causality results are also consistent with some of the previous association results obtained using standard logistic regressions. For example, we have estimated the OR of T2D per 488 g lower of birth weight to be 1.41, which is very close to a previous meta-analysis estimate obtained using logistic regression where the OR of T2D per 500 g lower of birth weight is estimated to be 1.47 (Harder et al., 2007). We have estimated the OR of CAD per 488 g lower of birth weight to be 1.34, which is also close to that obtained from a birth cohort study where the OR of CAD for a 500 g decrease in birth weight is estimated to be 1.27 (Lawlor et al., 2005). Our conclusions of T2D and CAD here are also consistent with those previously derived by a genetic risk score regression (Wang et al., 2016) and a similar MR analysis (Zanetti et al., 2018). In addition, our results suggest that the inverse causal associations of birth weight with CAD, MI, or T2D are not likely mediated by other risk factors such as blood pressures or adult weight, again in line with previous studies (Ruiz-Narvaez et al., 2014). Nevertheless, we also acknowledge that our results may appear to be inconsistent with those in (Zanetti et al., 2018) in terms of detecting the causal effects of birth weight on LDL, BMI, and 2-h glucose. However, for LDL and BMI, we note that our results are based on a more stringent p-value significance threshold adjusted by Bonferroni correction (to adjust for the multiple traits examined). Our results of birth weight on LDL or BMI are indeed marginally significant based on the normal p-value threshold of 0.05 with expected effect direction (i.e., negative effect on LDL and positive effect on BMI), and are thus consistent with (Zanetti et al., 2018). For 2-h glucose, we suspect that the difference in the SNP instruments used may lead to different power and thus different results. Importantly, compared to those previous MR studies (Au Yeung et al., 2016; Wang et al., 2016; Zanetti et al., 2018), our study has the following unique advantages: i) we employed a larger number of valid instruments that were obtained from larger scale GWASs; ii) we performed a more comprehensive analysis by considering a larger set of adult diseases and mediators; and iii) we carried out much more extensive sensitivity analyses and simulations to guarantee the robustness of our results, including sensitivity analyses with regard to pleiotropy and maternal effects.

### Public Health Implications of Our Results

Our results on the causal effects of birth weight on multiple adult diseases have important implications from a public health perspective. The benefits of reasonably high birth weight in terms of reducing the risks of adult diseases suggest that strategies to increase birth weight (e.g., through iron supplement; Cogswell et al., 2003) may achieve health gains in later life. Importantly, such public health policy towards improving birth weight may have added more benefits in the developing counties than in the developed counties. For example, half of world’s low birth weight infants are born in South Asia (Sachdev, 2001); nutrition-based intervention towards improving birth weight (Ramakrishnan, 2004) there may help curb the unusually high risks of CAD (Nair and Prabhakaran, 2012), MI (Wilkinson et al., 1996), and T2D (Gujral et al., 2013) in these developing counties (e.g., India, Pakistan, and Nepal). Additionally, as birth weight is often tied with social/economic status (Chomitz et al., 1995), some of these strategies intervening the modifiable risk factors to birth weight may have a higher impact in developing countries than in developed countries. Nevertheless, we also caution that, despite the potential benefits, increasing birth weight may have unwanted side effects. For example, it has been shown that extremely high birth weight can lead to childhood obesity, which is itself related to a series of poor health outcomes, likely due to the potentially U-shaped relationship between birth weight and various adult diseases (Harder et al., 2007). In addition, from a theoretical evolutionary biology perspective, growth and reproduction may be expected to trade-off against longevity; thus, growth promotion may reduce longevity. Careful examination of the potential benefits and side effects of high birth weight is needed in future studies.

### Confounding of the Maternal Effects

Maternal effect is an important confounder for causal effect estimation of birth weight on adult diseases. In the present study, we have performed extensive simulations under realistic assumptions to examine the extent to which maternal effects may influence or bias causal effect estimation. In addition, we have carried out sensitivity analysis by carefully removing instruments that may exhibit potential maternal effects on offspring’s birth weights as well as sensitivity analysis by using a genetic-score based approach to directly control for maternal genetic effects. Our simulations and sensitivity analyses support the robustness of our causal effect estimation results and suggest that maternal effects unlikely affect causal effect estimation in the present study. The robustness of our results with respect to maternal effect confounding is consistent with the early study of Horikoshi et al. (2016), which discovered that fetal effects, rather than maternal effects, mainly drive the discovered causal associations between birth weight and adult diseases. In particular, Horikoshi et al. (2016) provided two lines of supporting evidence: i) fetal SNPs explain about six times more heritability of birth weight than maternal effects [0.24 (se = 0.11) vs. 0.04 (se = 0.10)]; and ii) among all identified loci associated with birth weight, 93% SNPs exhibit larger fetal effects than maternal effects. Nevertheless, despite the strong evidence, we acknowledge that we do not have access to individual-level genotype information in the present study and that the data sets we have analyzed here do not contain mother–child pairs. Accurate disentangling of maternal and offspring effects on adult outcomes in the absence of child–mother pairs and/or individual-level genotype information is challenging and remains an active area of research (Eaves et al., 2014; Lawlor et al., 2017; Warrington et al., 2018). Therefore, we remain cautious on our results and leave the study of maternal effect confounding as an important topic for future investigations.

### Limitations of Our Study

Our analysis results are not without limitations. First, we acknowledge that there was a small overlap between individuals used in the EGG GWAS for birth weight (**Table S1**) and individuals used in the DIAGRAM GWAS for T2D (**Table S11**), suggesting that a small set of individuals are simultaneously used to obtain SNP effect size estimates for both birth weight and T2D. In particular, the European Prospective Investigation into Cancer and Nutrition (EPIC) study was included in both these two aforementioned GWASs with an overlapping sample size of ∼9,000 individuals (8,939 in EGG and 9,292 in DIAGRAM). Sample overlapping is commonly encountered in GWAS-based MR analysis (Burgess et al., 2016) and can result in model overfitting and biased causal effect estimates. However, the proportion of individuals in the EPIC study is relatively small and represents only 6.22% of the EGG study and 5.86% of the DIAGRAM study, suggesting that the bias resulting from overlapped samples is neglectable (Burgess et al., 2016). In addition, there is no overlap between samples used in EGG (for birth weight) (**Table S1**) and samples used in CARDIoGRAMplusC4D (for CAD and MI) (**Table S12**). Second, for some complex traits, we had to use GWASs with relatively small samples due to data availability reasons. For example, we had to use summary statistics for blood pressures from the Atherosclerosis Risk in Communities (ARIC) GWAS cohort data (**Text S2**) (Richey Sharrett, 1992) with only 8,749 individuals. The ARIC sample size is small compared with the previous largest GWAS meta-analysis for blood pressure that includes ∼200,000 individuals (The International Consortium for Blood Pressure Genome-Wide Association Studies., 2011). However, this largest GWAS for blood pressure only released summary statistics in terms of the absolute effect size estimate but without the effect size direction/sign, and thus cannot be used in the present study. Besides the largest GWAS of blood pressure, we also examined the UK Biobank data (Sudlow et al., 2015) and obtained summary statistics available from the online MR-Base platform (Hemani et al., 2018) for blood pressures. Unfortunately, these two data sources contain part of the samples in the EGG study of birth weight without releasing detailed individual overlapping information, and thus cannot be used in the present study. Therefore, we had to use the ARIC data with a relatively small sample size and we emphasize that future research with larger samples to investigate blood pressures will likely be beneficial. Third, like many other MR applications, we have assumed a linear relationship between birth weight and adult diseases. It is certainly possible that non-linear relationships exist; for example, a U-shaped association pattern between birth weight and T2D was observed in a case control study for low birth weight (i.e., birth weight <2,500 g vs. > 2,500 g) (Harder et al., 2007). However, because birth weights for most individuals collected in the EGG study (Horikoshi et al., 2016) are in the normal range (95% range is 2,492–4,405 g; **Table S1**), a linearity assumption is likely a sensible choice for our study. Fourth, due to the use of GWAS summary statistics, we unfortunately cannot perform stratified analysis by gender and cannot estimate the causal effects of birth weight on adult diseases in males and females separately. Therefore, we are unable to validate different gender-specific causal effects of birth weight on adult diseases that are observed in early studies (Barker et al., 2002). Fifth, similar to other MR studies, our study cannot also be fully immune to confounding biases that are commonly encountered in observational studies. For example, birth weight is typically related to the socioeconomic position, which may not completely be ruled out in MR studies. Sixth, our study focuses only on European population, and future studies are needed to investigate whether our conclusions can be generalized to other human populations.

## Conclusion

Our results suggest that lower birth weight is causally associated with an increased risk of CAD, MI, and T2D in later life, supporting the fetal origins of adult diseases hypothesis.

## Data Availability

All datasets generated for this study are included in the manuscript and the supplementary files.

## Author Contributions

PZ and XZ conceived the idea for the study. PZ obtained the genetic data. PZ and XZ developed the study methods. PZ performed the data analyses. PZ and XZ interpreted the results of the data analyses. PZ and XZ wrote the manuscript.

## Funding

This study was supported by the National Institutes of Health (R01HG009124 and R01GM126553 to XZ), the National Science Foundation (DMS1712933 to XZ), Youth Foundation of Humanity and Social Science funded by Ministry of Education of China (18YJC910002 to PZ), the Natural Science Foundation of Jiangsu (BK20181472 to PZ), the project funded by China Postdoctoral Science Foundation (2018M630607 and 2019T120465 to PZ), the Project funded by Postdoctoral Science Foundation of Xuzhou Medical University (to PZ), QingLan Research Project of Jiangsu for Outstanding Young Teachers (to PZ), the National Natural Science Foundation of China (81402765 to PZ),the Statistical Science Research Project from National Bureau of Statistics of China (2014LY112 to PZ), and the Priority Academic Program Development of Jiangsu Higher Education Institutions (PAPD) for Xuzhou Medical University (to PZ).

## Conflict of Interest Statement

The authors declare that the research was conducted in the absence of any commercial or financial relationships that could be construed as a potential conflict of interest.

## Supplementary Material

Supplementary Texts for method details, supplementary Results, Tables, and Figures.
