# Supplementary material for: Causal Association Between Birth Weight and Adult Diseases: Evidence From a Mendelian Randomization Analysis
Source: Front Genet. 2019 Jul 10;10:618. doi: 10.3389/fgene.2019.00618 (PMC6635582; doi:10.3389/fgene.2019.00618)
Supplement: Supplementary file 1 [file DataSheet_1.zip › 2019-03-26 Supplementary Tables and Figures.pdf]

Supplementary Tables and Figures

Table S1 Studies used in the GWAS of birth weight

Studies	Sample Size (Male/Female)	Mean (SD)
1958 British Birth Cohort	4,595 (2,320/2,275)	3,359 (483)
ALSPAC: The Avon Longitudinal Study of Parents and Children	7,285 (3,722/3,563)	3,490 (476)
CHOP-Caucasian: The Children's Hospital of Philadelphia-Caucasian	9,405 (5,040/4,365)	3,398 (569)
CoLaus: The Cohorte Lausannoise	2,089 (892/1,197)	3,352 (675)
COPSAC-2000: The Copenhagen Prospective Study on Asthma in Childhood	352 (173/179)	3,555 (485)
COPSAC-2010: The Copenhagen Prospective Study on Asthma in Childhood	589 (306/283)	3,588 (481)
COPSAC-REGISTRY: The Copenhagen Prospective Study on Asthma in Childhood	1,210 (804/406)	3,553 (488)
DNBC: The Danish National Birth Cohort	915 (475/440)	3,699 (468)
ERF: The Erasmus Rucphen Family	459 (187/272)	3,039 (644)
EPIC: The European Prospective Investigation into Cancer and Nutrition	8,939 (3,448/5,491)	3,358 (772)
Fenland (GA+)	5,188 (2,088/3,100)	3,394 (555)
Fenland (GA-)	833 (509/324)	3,354 (624)
The Generation R study	2,701 (1,378/1,323)	3,574 (488)
GINIplus & LISAplus (GA+)	656 (360/296)	3,443 (415)
GINIplus & LISAplus (GA-)	790 (391/399)	3,423 (433)
GOYA: Genetics of Overweight Young Adults	290 (290/0)	3,553 (711)
HBCS: The Helsinki Birth Cohort Study	1,472 (639/833)	3,444 (454)
INMA: INfancia y Medio Ambiente [Environment and Childhood] Project	1,021 (527/494)	3,278 (423)
INTER99: The Danish Inter99 intervention study	4,243 (1,981/2,262)	3,433 (485)
Leipzig	597 (304/293)	3,527 (536)
NEO	3,719 (1,650/2,069)	3,514 (1,271)
NFBC1966: The Northern Finland Birth cohorts	5,009 (2,393/2,616)	3,541 (489)
NFBC1986: The Northern Finland Birth cohorts	4,680 (2,306/2,374)	3,572 (535)

NTR: The Netherlands Twin Register	1,265 (447/818)	3,343 (601)
ORCADES: The Orkney Complex Disease Study	960 (330/630)	3,488 (640)
PANIC	436 (231/205)	3,588 (474)
RAINE: The Western Australian Pregnancy Cohort	1,347 (693/654)	3,449 (470)
SORBS	298 (113/185)	3,393 (673)
STRIP	599 (311/288)	3,619 (465)
TEENAGE (GA+)	279 (126/153)	3,336 (445)
TEENAGE (GA-)	551 (234/317)	3,341 (449)
TDCOB-cases: The Danish Childhood Obesity Biobank	669 (391/278)	3,660 (540)
TDCOB-controls: The Danish Childhood Obesity Biobank	560 (211/349)	3,540 (502)
YFS: The Young Finns Study	1,915 (861/1,054)	3,572 (475)
UK Biobank	67,786 (40,425/27,361)	3,391 (420)
**Total**	143,702 (76,556/67,146)	3,449 (488)

The mean and standard deviation (SD) of birth weight (in grams) were computed by combing both male and female individuals (Horikoshi et al., 2016). The total sample size for the GWAS of birth weight is 143,702 (male 76,556 and female 67,146), and the median of mean and SD for birth weight across the 35 studies are 3,449 and 488 grams, respectively. Note that a total of 143,677 individuals were used for the SNPs analyzed in the present study, which is slightly smaller than total sample size (143,702) available for all SNPs.

Table S2 Summary information for the 48 autosomal SNPs that are used as instrumental variables in the Mendelian randomisation analysis

Chr	SNP	Position	Gene	Allele	MAF	BETA	SE	*p*	*N*	PVE	*F*
7	rs138715366	44,246,271	*YKT6-GCK*	T/C	0.01	-0.2412	0.0229	7.20E-26	132,343	8.38E-04	110.99
17	rs144843919	29,037,339	*SUZ12P1-CRLF3*	A/G	0.04	-0.0660	0.0116	1.40E-08	121,357	2.67E-04	32.41
3	rs13322435	156,795,468	*CCNL1-LEKR1*	G/A	0.40	-0.0533	0.0040	3.60E-41	139,426	1.27E-03	177.29
6	rs35261542	20,675,792	*CDKAL1*	A/C	0.27	-0.0444	0.0041	4.40E-27	143,667	8.16E-04	117.33
12	rs1351394	66,351,826	*HMGA2*	C/T	0.51	-0.0436	0.0037	1.90E-32	143,671	9.66E-04	138.92
2	rs7575873	23,962,647	*ATAD2B*	G/A	0.12	-0.0384	0.0057	1.20E-11	139,425	3.25E-04	45.33
6	rs1101081	152,032,917	*ESR1*	T/C	0.28	-0.0376	0.0042	1.60E-19	139,427	5.74E-04	80.08
21	rs2229742	16,339,172	*NRIP1*	C/G	0.13	-0.0360	0.0060	2.20E-09	143,672	2.51E-04	36.07
4	rs925098	17,919,811	*LCORL*	A/G	0.73	-0.0340	0.0042	5.40E-16	139,426	4.70E-04	65.56
9	rs700059	125,824,055	*STRBP*	A/G	0.86	-0.0334	0.0054	4.70E-10	139,427	2.74E-04	38.21
8	rs13266210	41,533,514	*ANK1-NKX6-3*	G/A	0.21	-0.0308	0.0045	1.30E-11	139,429	3.36E-04	46.86
10	rs61862780	94,468,643	*HHEX-IDE*	C/T	0.49	-0.0281	0.0037	3.00E-14	143,670	4.01E-04	57.63
5	rs854037	57,091,783	*5q11.2*	G/A	0.19	-0.0268	0.0048	2.20E-08	139,429	2.24E-04	31.24
7	rs11765649	23,479,013	*IGF2BP3*	C/T	0.25	-0.0267	0.0043	5.80E-10	139,428	2.76E-04	38.49
20	rs28530618	31,275,581	*C20orl203*	G/A	0.51	-0.0261	0.0038	7.70E-12	138,162	3.41E-04	47.13
6	rs1415701	130,345,835	*L3MBTL3*	A/G	0.26	-0.0253	0.0043	2.60E-09	143,666	2.41E-04	34.63
12	rs139975827	22,068,161	*ABCC9*	A/G	0.38	-0.0248	0.0043	1.10E-08	123,203	2.70E-04	33.27
7	rs798489	2,801,803	*GNA12*	T/C	0.27	-0.0233	0.0042	2.00E-08	143,670	2.14E-04	30.75
15	rs7402982	99,193,269	*IGF1R*	G/A	0.57	-0.0232	0.0039	2.30E-09	139,423	2.54E-04	35.42
8	rs12543725	142,247,979	*SLC45A4*	A/G	0.41	-0.0231	0.0038	1.20E-09	139,431	2.65E-04	36.96
7	rs6959887	35,295,365	*TBX20*	G/A	0.39	-0.0228	0.0038	1.50E-09	143,659	2.51E-04	36.07
15	rs12906125	91,427,612	*FES*	A/G	0.32	-0.0228	0.0040	1.70E-08	141,281	2.30E-04	32.50
22	rs134594	29,468,456	*KREMEN1*	T/C	0.65	-0.0227	0.0040	1.00E-08	137,340	2.34E-04	32.14
3	rs10935733	148,622,968	*CPA3*	C/T	0.59	-0.0221	0.0039	9.20E-09	139,426	2.30E-04	32.07
3	rs2242116	46,941,116	*PTH1R*	G/A	0.62	-0.0216	0.0038	1.40E-08	143,670	2.25E-04	32.33

12	rs12823128	26,872,730	*ITPR2*	C/T	0.46	-0.0211	0.0037	1.90E-08	139,431	2.33E-04	32.49
10	rs2421016	124,167,512	*PLEKHA1*	T/C	0.49	0.0207	0.0037	1.80E-08	143,659	2.18E-04	31.32
9	rs2150052	113,945,067	*LPAR1*	T/A	0.50	0.0211	0.0038	2.20E-08	139,424	2.21E-04	30.82
20	rs6040076	10,658,882	*JAG1*	C/G	0.49	0.0231	0.0039	2.00E-09	139,424	2.52E-04	35.14
11	rs10830963	92,708,710	*MTNR1B*	G/C	0.28	0.0232	0.0042	2.90E-08	143,663	2.12E-04	30.46
9	rs7847628	123,631,225	*PHF19*	G/A	0.68	0.0233	0.0041	1.00E-08	139,424	2.32E-04	32.35
20	rs6016377	39,172,728	*MAFB*	T/C	0.43	0.0239	0.0039	9.50E-10	139,425	2.69E-04	37.51
5	rs7729301	157,886,953	*EBF1*	A/G	0.73	0.0239	0.0042	1.60E-08	143,669	2.25E-04	32.33
4	rs6537307	145,601,863	*HHIP*	G/A	0.50	0.0254	0.0037	9.50E-12	139,431	3.38E-04	47.14
6	rs7742369	34,165,721	*HMGA1*	G/A	0.18	0.0283	0.0049	9.90E-09	137,045	2.43E-04	33.31
1	rs3753639	154,986,091	*ZBTB7B*	C/T	0.24	0.0306	0.0045	7.30E-12	138,162	3.35E-04	46.30
17	rs113086489	7,171,356	*CLDN7*	T/C	0.56	0.0307	0.0038	9.10E-16	139,426	4.68E-04	65.28
1	rs72480273	161,644,871	*FCGR2B*	C/A	0.17	0.0313	0.0051	8.00E-10	138,380	2.72E-04	37.65
1	rs2473248	22,536,643	*WNT4-ZBTB40*	C/T	0.87	0.0325	0.0057	1.00E-08	139,428	2.33E-04	32.49
13	rs1819436	78,580,283	*RNF219-AS1*	C/T	0.87	0.0329	0.0057	6.30E-09	138,979	2.40E-04	33.36
10	rs7076938	115,789,375	*ADRB1*	T/C	0.73	0.0363	0.0042	4.70E-18	143,671	5.20E-04	74.75
12	rs7964361	102,994,878	*IGF1*	A/G	0.09	0.0391	0.0067	4.70E-09	139,428	2.44E-04	34.03
3	rs11719201	123,068,744	*ADCY5*	T/C	0.23	0.0463	0.0044	2.40E-26	143,670	7.70E-04	110.71
2	rs1374204	46,484,205	*EPAS1*	T/C	0.70	0.0470	0.0042	6.20E-29	134,453	9.31E-04	125.29
22	rs62240962	42,259,524	*SREBF2*	C/T	0.08	0.0470	0.0070	9.70E-12	143,677	3.14E-04	45.13
11	rs72851023	2,130,620	*INS-IGF2*	T/C	0.07	0.0476	0.0075	2.90E-10	135,776	2.97E-04	40.34
7	rs62466330	73,056,805	*MLXIPL*	C/T	0.07	0.0486	0.0075	1.20E-10	142,200	2.95E-04	41.96
9	rs28510415	98,245,026	*PTCH1*	G/A	0.09	0.0557	0.0065	1.50E-17	134,746	5.45E-04	73.48

The summary data of instruments for birth weight can be available from http://egg-consortium.org/. These SNPs are associated with birth weight at the genome-wide significance level (p < 5.00E-08) in a meta-analysis with up to 143,677 individuals of European ancestry (Horikoshi et al., 2016). SNPs are ordered based on their effect size estimates. Chr: chromosome; SNP: single-nucleotide polymorphism id; Position: genome

position in base pair; Allele: effect allele and alternative allele; MAF: minor allele frequency; BETA: SNP effect size, SE: standard error; PVE: proportion of variance in birth weight explained by the SNP; *p*, *N*, and *F* represent p value, sample size, and *F* statistic, respectively.

Table S3 Potential pleiotropic instruments excluded for each complex adult outcome

Traits	Excluded instruments that show potential pleiotropic association with the outcome variable
Age-related Macular Degeneration	rs79237883
Celiac Disease	rs2229742
Coronary Artery Disease	rs79237883, rs12906125
Inflammatory Bowel Disease	rs798498
Ischemic Stroke	rs13266210, rs4144829
Multiple Sclerosis	rs2473248, rs72480273, rs11719201, rs9368777, rs11765649, rs2497304, rs113086489
Myocardial Infarction	rs4144829, rs79237883, rs12906125
Parkinson	rs79237883
Primary Sclerosing Cholangitis	rs2497304
Systemic Lupus Erythematosus	rs72480273, rs6016377
Type 1 Diabetes	rs72851023
Type 2 Diabetes	rs11719201, rs35261542, rs13266210, rs2497304, rs7998537
Type 2 Diabetes adjusted with BMI	rs11719201, rs35261542, rs13266210, rs2497304
Ulcerative Colitis	rs798498
2hrGlucose	rs11719201
Adiponectin	rs2168443, rs113086489
Birth Length	rs3753639, rs17034876, rs4144829, rs2131354, rs1187118, rs3780573, rs10818797, rs740746, rs1819436, rs41311445
BMI	rs3753639, rs11719201, rs79237883, rs72851023, rs28415607
BMI_Child	rs28415607, rs41311445
Body Fat	rs11719201
College	rs9368777
EduYears	rs9368777
Fasting Glucose	rs11719201, rs138715366
Growth_PG	rs3780573
Head Circumference	rs11765649

Height	rs3753639, rs7575873, rs17034876, rs11719201, rs4144829, rs740746, rs7964361, rs1819436, rs12906125, rs7402982, rs144843919, rs2131354, rs9368777, rs1187118, rs798498, rs11765649, rs7854962, rs3780573,
High Density Lipoproteins	rs10872678, rs740746
Hip Circumference	rs3753639, rs2131354, rs798498, rs79237883, rs72851023, rs28415607, rs2229742
Leptin	rs900399
Low Density Lipoproteins	rs6016377
Overweight	rs79237883
Serum Urate	rs111778406
Total Cholesterol	rs2306547, rs6016377
Waist Circumference	rs900399, rs1187118, rs79237883, rs72851023, rs28415607, rs2229742
Waist-to-hip ratio	rs3753639, rs900399, rs134594

We excluded instruments that show horizontal pleiotropic associations with the outcome to ensure the validity of MR analysis if the selected SNPs had p values less than 1.06E-3 (=0.05/47) for a specific trait. The number of instruments excluded varied can vary for different outcomes and ranges from 1 (e.g. for age-related macular degeneration) to 18 (e.g. for height). Note that, among the removed instruments, rs11719201 and rs79237883 were excluded for eight traits; and diseases or complex traits that have zero potentially pleiotropic instruments are not included in the table.

Table S4 Summary information for seven instruments for birth weight obtained from Horikoshi et al (2013) (Horikoshi et al., 2013)

Chr	SNP	Position	Gene	Allele	MAF	2013				2016			
						Beta	SE	*p*	*N*	Beta	SE	*p*	*N*
3	rs900400	158,281,469	*CCNL1*	C/T	0.39	-0.072	0.006	3.58E-38	61,142	-0.053	0.004	1.10E-40	139,425
3	rs9883204	124,579,510	*ADCY5*	C/T	0.24	-0.059	0.006	5.48E-20	61,509	-0.041	0.004	8.40E-22	143,666
12	rs1042725	64,644,614	*HMGA2*	T/C	0.50	-0.047	0.005	1.43E-19	68,655	-0.043	0.004	3.10E-32	143,673
6	rs6931514	20,811,931	*CDKAL1*	G/A	0.29	-0.050	0.006	1.50E-18	68,822	-0.043	0.004	6.20E-26	143,582
5	rs4432842	57,207,835	*5q11.2*	C/T	0.30	-0.034	0.006	4.56E-8	53,619	-0.021	0.004	3.30E-07	139,430
4	rs724577	17,602,508	*LCORL*	C/A	0..27	-0.042	0.006	4.60E-11	55,877	-0.033	0.004	1.70E-15	143,674
10	rs1801253	115,795,046	*ADRB1*	G/A	0.26	-0.041	0.007	3.60E-9	49,660	-0.032	0.004	3.00E-14	143,237

The summary data sets of birth weight can be available from http://egg-consortium.org/. For comparison, the summary information for these instruments available from Horikoshi et al (2016) (Horikoshi et al., 2016) is also shown.

Table S5 Ten independent maternal SNPs associated with offspring birth weight (p < 5E-8) in a European ancestry meta-analysis of up to 86,577 individuals

Chr	SNP	POS	Gene	Allele	BETA	SE	*p*	*N*
3	rs7629460	155,829,938	*KCNAB*	A/C	-0.0393	0.0065	1.60E-09	48,632
5	rs12520982	157,894,747	*EBF1*	T/C	0.0414	0.0072	9.90E-09	48,632
6	rs9375694	130,356,608	*L3MBTL3*	A/G	-0.0346	0.0058	2.10E-09	68,223
7	rs2971669	44,231,778	*GTCK*	T/C	0.0382	0.0065	5.50E-09	68,162
7	rs45446698	99,332,948	*CYP3A7*	T/G	-0.0892	0.0160	2.30E-08	48,632
10	rs7903146	114,758,349	*TCF7L2*	T/C	0.0335	0.0059	1.20E-08	68,253
11	rs10830963	92,708,710	*MTNR8B*	C/G	-0.0524	0.0058	1.00E-19	71,341
12	rs1351394	66,351,826	*HMGA4*	T/C	0.0340	0.0053	1.40E-10	68,247
12	rs3184504	111,884,608	*SH2B4*	T/C	-0.0325	0.0053	6.90E-10	68,249
19	rs2918299	8,787,273	*ACTL9*	T/C	-0.0411	0.0074	2.20E-08	67,603

The summary data of instruments for birth weight can be available from http://egg-consortium.org/. These SNPs are obtained from the summary results from the maternal genome-wide meta-analyses of offspring birth weight (Beaumont et al., 2018), where over 8 million variants on 86,577 women from the first release of the UK Biobank genetics data were analyzed.

Table S6 Estimated causal effect, Q statistic and statistical power for detecting non-zero causal effects of lower birth weight on various diseases

Diseases	Sample Size (Case/Control)	Estimated Causal Effect (SE)		I^2(%)	Q	p value of Q	Power		2013
		without pleiotropic instruments	with pleiotropic instruments				Expected	Observed	
Age-related Macular Degeneration	33,976 (16,144/17,832)	0.98 (0.06)	0.96 (0.07)	9.2	49.6	2.61E-01	0.03	0.00	1.18 (0.12)
Alzheimer	54,162 (17,008/37,154)	1.06 (0.10)	1.06 (0.10)	12.4	52.5	2.06E-01	0.05	0.01	1.27 (0.13)
Ankylosing Spondylitis	6,588 (1,788/4,800)	0.82 (0.25)	0.82 (0.25)	21.3	58.4	8.62E-02	0.01	0.02	1.34 (0.46)
Celiac Disease	15,283 (4,533/10,750)	0.83 (0.19)	0.84 (0.23)	38.1	72.7	4.21E-03	0.01	0.04	1.13 (0.69)
Chronic Kidney Disease	84,740 (7,173/77,567)	1.00 (0.05)	1.00 (0.05)	5.2	29.5	3.35E-01	0.02	0.00	1.02 (0.15)
**Coronary Artery Disease**	**184,305 (60,801/123,504)**	**1.34 (0.07)**	**1.42 (0.08)**	0.0	32.0	7.41E-01	**0.31**	**1.00**	**1.24 (0.11)**
Crohn's Disease	20,883 (5,956/14,927)	1.19 (0.14)	1.19 (0.14)	21.4	59.8	8.31E-02	0.01	0.07	1.42 (0.22)
Hypertension	4,890 (1,952/2,938)	0.99 (0.30)	0.99 (0.30)	38.9	72.1	3.59E-03	0.00	0.00	0.73 (0.34)
Inflammatory Bowel Disease	34,652 (12,882/21,770)	1.09 (0.13)	1.05 (0.13)	42.7	80.2	9.62E-04	0.02	0.01	1.24 (0.14)
Ischaemic Stroke	446,696 (40,585/406,111)	0.97 (0.06)	0.99 (0.05)	47.7	83.1	5.00E-04	0.29	0.01	1.00 (0.13)
Multiple Sclerosis	15,474 (10,299/5,175)	1.27 (0.23)	1.73 (0.31)	58.1	93.0	1.64E-06	0.01	0.10	2.44 (0.34)
**Myocardial Infarction**	**166,065 (42,561/123,504)**	**1.30 (0.07)**	**1.42 (0.09)**	33.1	65.8	1.42E-02	**0.22**	**1.00**	**1.25 (0.13)**
Parkinson	8,477 (4,238/4,239)	1.25 (0.29)	1.45 (0.36)	0.0	22.9	4.09E-01	0.01	0.04	1.63 (0.70)
Primary Biliary Cirrhosis	13,239 (2,764/10,475)	1.26 (0.15)	1.26 (0.15)	51.9	79.0	7.04E-05	0.01	0.06	1.70 (0.39)
Primary Sclerosing Cholangitis	24,751 (4,796/19,955)	1.21 (0.19)	1.34 (0.21)	0.6	40.2	4.15E-01	0.01	0.08	1.75 (0.26)
Rheumatoid Arthritis	4,798 (1,860/2,938)	0.89 (0.26)	0.89 (0.26)	21.7	56.2	8.56E-02	0.01	0.00	0.89 (0.21)
Systemic Lupus Erythematosus	23,210 (7,219/15,991)	0.76 (0.18)	0.68 (0.20)	12.5	44.6	2.14E-01	0.02	0.26	0.88 (0.41)
Type 1 Diabetes	4,901 (1,963/2,938)	1.06 (0.26)	1.20 (0.31)	14.9	50.5	1.72E-01	0.01	0.00	1.49 (0.35)
**Type 2 Diabetes**	**159,208 (26,676/132,532)**	**1.41 (0.11)**	**1.89 (0.18)**	48.9	82.3	1.40E-04	**0.13**	**1.00**	**2.68 (0.44)**
**Type 2 Diabetes adjusted with BMI**	**159,208 (26,676/132,532)**	**1.54 (0.11)**	**2.09 (0.19)**	31.8	63.1	1.92E-02	**0.13**	**1.00**	**3.32 (0.47)**
Ulcerative Colitis	27,432 (6,968/20,464)	1.02 (0.15)	0.95 (0.17)	33.2	68.9	1.24E-02	0.02	0.00	1.16 (0.17)

The sources of the sumamry data for these diseases are shown in Text S1 and Text S5. SE in parentheses is the standard error of the estimated causal effect for birth weight using the random-effects IVW method. The causal effects estimated with or without potentially pleiotropic instruments for all diseases are largely similar (note that estimates for certain diseases are identical in the two cases as no pleiotropic instruments were identified for these diseases). In our analyses we mainly focus on the estimates obtained without potentially pleiotropic instruments. I^2 and Q are two statistics for the heterogeneity test. Statistical power (last two columns) is computed using the method given in (Brion et al., 2013) by

setting PVE = 1.7% and the significance level α to 2.38E-3 (=0.05/21) and ignoring uncertainty. The expected power is computed by assuming the causal effect of birth weight on disease is OR = 1.10 or 0.90, while the observed power is computed based on the estimated causal effect for each disease. The estimated power for CAD, MI, T2D and T2D_BMI is highlighted in red. The power was calculated by using the method presented in (Brion et al., 2013) that is implemented online at https://cnsgenomics.shinyapps.io/mRnd/. 2013 in the last column represents the estimates of causal effects for these diseases using the seven instruments obtained from Horikoshi et al (2013) (Horikoshi et al., 2013).

Table S7 Used 57 adult diseases in the UK Biobank dataset

ID	description	*n*	controls	cases	*p* value	
					2013	2016
1	Alzheimer's disease	361,194	361,075	119	8.02E-01	6.30E-01
2	Type 2 diabetes with peripheral circulatory complications	361,194	361,067	127	4.85E-01	8.23E-01
3	Diagnoses - main ICD10: M45 Ankylosing spondylitis	361,194	361,048	146	9.68E-01	1.38E-01
4	Non-cancer illness code, self-reported: inflammatory bowel disease	361,141	360,991	150	7.33E-02	2.97E-01
5	Rheumatism, unspecified	361,194	361,044	150	5.21E-01	6.23E-01
6	Dementia	361,194	361,037	157	8.42E-01	2.10E-01
7	Other ulcerative colitis	361,194	361,035	159	9.11E-01	2.11E-01
8	Type 1 diabetes with ophthalmic complications	361,194	361,028	166	3.67E-01	2.97E-01
9	Type 1 diabetes with ketoacidosis	361,194	361,026	168	7.00E-01	9.28E-01
10	Non-cancer illness code, self-reported: macular degeneration	361,141	360,929	212	3.32E-01	2.66E-01
11	Type 2 diabetes with ophthalmic complications	361,194	360,958	236	9.66E-01	8.16E-01
12	Any dementia	361,194	360,951	243	7.34E-01	2.78E-01
13	Type 1 diabetes without complications	361,194	360,947	247	3.11E-01	4.03E-01
14	Crohn's disease of small interstine	361,194	360,945	249	8.07E-01	4.02E-02
15	Diagnoses - main ICD10: K74 Fibrosis and cirrhosis of liver	361,194	360,942	252	9.12E-01	8.26E-01
16	Non-cancer illness code, self-reported: liver failure/cirrhosis	361,141	360,877	264	2.52E-01	2.35E-01
17	Cholangitis (sclerosing)	361,194	360,907	287	9.88E-02	1.80E-01
18	Diagnoses - main ICD10: M05 Seropositive rheumatoid arthritis	361,194	360,906	288	5.11E-02	2.50E-01
19	Other/unspecified seropositive rheumatoid arthritis	361,194	360,895	299	5.34E-02	3.56E-01
20	Non-cancer illness code, self-reported: type 1 diabetes	361,141	360,823	318	9.64E-01	7.65E-01
21	Seropositive rheumatoid arthritis	361,194	360,867	327	6.13E-02	2.52E-01
22	Crohn's disease of large intestine	361,194	360,858	336	7.02E-01	7.54E-02
23	Non-cancer illness code, self-reported: systemic lupus erythematosis/sle	361,141	360,726	415	5.31E-01	9.19E-01
24	Type 2 diabetes without complications	361,194	360,740	454	3.95E-03	3.97E-01

25	Diagnoses - main ICD10: I22 Subsequent myocardial infarction	361,194	360,730	464	8.39E-01	2.82E-01
26	Type 1 diabetes	361,194	360,611	583	8.48E-02	2.37E-01
27	Non-cancer illness code, self-reported: Parkinson’s disease	361,141	360,489	652	9.35E-01	8.13E-01
28	Chron's disease NAS	361,194	360,430	764	5.84E-01	1.61E-01
29	Diagnoses - main ICD10: I10 Essential (primary) hypertension	361,194	360,407	787	3.15E-01	3.55E-01
30	Coeliac disease	361,194	360,352	842	5.46E-01	5.67E-01
31	Type 2 diabetes	361,194	360,306	888	2.36E-01	2.82E-01
32	Diagnoses - main ICD10: K50 Crohn's disease [regional enteritis]	361,194	360,226	968	5.29E-01	4.20E-01
33	Non-cancer illness code, self-reported: ankylosing spondylitis	361,141	360,103	1,038	8.68E-01	9.66E-01
34	Non-cancer illness code, self-reported: Crohn’s disease	361,141	360,045	1,096	6.33E-01	9.14E-01
35	Hypertension	361,194	359,957	1,237	2.42E-01	1.84E-01
36	Other/unspecified rheumatoid arthritis	361,194	359,913	1,281	2.90E-01	5.30E-01
37	Hypertensive diseases	361,194	359,881	1,313	1.92E-01	9.02E-02
38	Non-cancer illness code, self-reported: multiple sclerosis	361,141	359,815	1,326	7.18E-01	5.73E-01
39	Diagnoses - main ICD10: M06 Other rheumatoid arthritis	361,194	359,793	1,401	3.97E-01	7.35E-01
40	Non-cancer illness code, self-reported: malabsorption/coeliac disease	361,141	359,554	1,587	3.84E-01	4.38E-02
41	Rheumatoid arthritis	361,194	359,589	1,605	2.88E-01	8.63E-01
42	Non-cancer illness code, self-reported: essential hypertension	361,141	359,483	1,658	7.79E-01	5.21E-01
43	Ulcerative colitis, NAS	361,194	359,291	1,903	3.17E-01	9.54E-01
44	Non-cancer illness code, self-reported: ulcerative colitis	361,141	359,225	1,916	1.30E-01	9.77E-01
45	Diagnoses - main ICD10: K51 Ulcerative colitis	361,194	359,051	2,143	1.96E-01	8.95E-01
46	Non-cancer illness code, self-reported: type 2 diabetes	361,141	358,849	2,292	9.94E-01	1.33E-01
47	Eye problems/disorders: Macular degeneration	117,890	115,164	2,726	5.19E-01	8.64E-01
48	Ischaemic Stroke, excluding all hemorrhage’s	361,194	357,880	3,314	8.00E-01	4.17E-01
49	Non-cancer illness code, self-reported: rheumatoid arthritis	361,141	357,124	4,017	3.23E-01	2.63E-01
50	Diagnoses - main ICD10: I21 Acute myocardial infarction	361,194	355,246	5,948	7.64E-01	5.16E-02
51	Myocardial infarction	361,194	354,176	7,018	6.24E-01	7.99E-02
52	Myocardial infarction, strict	361,194	354,176	7,018	6.24E-01	7.99E-02

53	Non-cancer illness code, self-reported: heart attack/myocardial infarction	361,141	352,902	8,239	5.41E-01	2.42E-01
54	Major coronary heart disease event	361,194	351,037	10,157	9.25E-01	5.07E-02
55	Major coronary heart disease event excluding revascularizations	361,194	351,037	10,157	9.25E-01	5.07E-02
56	Non-cancer illness code, self-reported: hypertension	361,141	267,581	93,560	1.45E-01	5.91E-01
57	Vascular/heart problems diagnosed by doctor: High blood pressure	360,420	263,281	97,139	9.22E-01	4.43E-01

Here 2013 represents the MR results estimated using instruments obtained from the GWAS of birth weight in Horikoshi et al (2013) (Horikoshi et al., 2013); and 2016 represents the MR results estimated using instruments obtained from the GWAS of birth weight in Horikoshi et al (2016) (Horikoshi et al., 2016). The summary data sets of birth weight can be available from http://egg-consortium.org/ and the UK Biobank data sets can be available from http://www.nealelab.is/uk-Biobank/.

Table S8 Estimated causal effects for the 21 diseases on birth weight in the reverse causation analysis

Diseases	2013			2016			UK Biobank		
	Beta	SE	*p*	Beta	SE	*p*	Beta	SE	*p*
Age-related Macular Degeneration	7.73E-03	1.74E-02	6.57E-01	-9.73E-05	5.81E-03	9.87E-01	4.32E-05	4.72E-03	9.93E-01
Alzheimer	-2.51E-03	4.40E-02	9.54E-01	2.78E-03	4.94E-03	5.74E-01	1.01E-03	4.26E-03	8.12E-01
Ankylosing Spondylitis	1.58E-03	1.63E-02	9.23E-01	1.86E-04	2.44E-03	9.39E-01	5.55E-03	1.79E-03	1.94E-03
Celiac Disease	-1.56E-02	1.25E-02	2.11E-01	-6.19E-03	9.85E-03	5.30E-01	1.67E-02	1.29E-02	1.97E-01
Chronic Kidney Disease	1.53E-02	3.68E-02	6.77E-01	-2.06E-02	1.72E-02	2.31E-01	-3.98E-03	1.56E-02	7.98E-01
Coronary Artery Disease	-2.91E-03	2.64E-02	9.12E-01	6.42E-03	9.18E-03	4.84E-01	7.94E-03	1.28E-02	5.35E-01
Crohn's Disease	5.13E-03	1.16E-02	6.58E-01	-5.36E-04	3.50E-03	8.78E-01	4.83E-03	3.89E-03	2.15E-01
Hypertension	-1.30E-02	3.20E-02	6.89E-01	1.55E-02	1.00E-02	1.22E-01	-2.17E-03	7.99E-03	7.86E-01
Inflammatory Bowel Disease	2.62E-04	1.16E-02	9.82E-01	-5.19E-03	4.13E-03	2.09E-01	5.89E-03	4.74E-03	2.13E-01
Ischaemic Stroke	8.05E-05	1.94E-02	9.97E-01	-2.40E-03	5.59E-03	6.68E-01	4.71E-03	4.71E-03	3.17E-01
Multiple Sclerosis	-1.52E-04	3.42E-03	9.65E-01	1.46E-04	8.43E-04	8.62E-01	-6.64E-04	7.77E-04	3.93E-01
Myocardial Infarction	-3.03E-03	3.12E-02	9.22E-01	4.01E-03	8.98E-03	6.55E-01	-2.03E-03	1.73E-02	9.06E-01
Parkinson	2.44E-02	2.81E-02	3.86E-01	-1.50E-02	1.25E-02	2.30E-01	1.03E-02	1.00E-02	3.03E-01
Primary Biliary Cirrhosis	4.15E-03	6.78E-03	5.41E-01	-3.58E-03	4.66E-03	4.43E-01	3.00E-03	4.48E-03	5.03E-01
Primary Sclerosing Cholangitis	7.42E-03	1.19E-02	5.34E-01	5.55E-03	5.86E-03	3.43E-01	1.34E-03	6.90E-03	8.46E-01
Rheumatoid Arthritis	-1.79E-03	9.83E-03	8.56E-01	-4.30E-03	3.16E-03	1.73E-01	3.49E-03	2.28E-03	1.25E-01
Systemic Lupus Erythematosus	7.00E-03	1.40E-02	6.28E-01	0.00E+00	6.00E-03	9.44E-01	1.40E-02	5.00E-03	4.00E-03
Type 1 Diabetes	4.84E-03	6.89E-03	4.82E-01	5.99E-04	3.36E-03	8.59E-01	1.21E-03	3.37E-03	7.19E-01
Type 2 Diabetes	-1.18E-02	3.21E-02	7.12E-01	4.01E-03	8.98E-03	6.55E-01	3.30E-02	1.65E-02	4.56E-02
Type 2 Diabetes adjusted with BMI	-5.45E-02	4.64E-02	2.40E-01	4.01E-03	8.98E-03	6.55E-01	3.29E-02	1.90E-02	8.35E-02
Ulcerative Colitis	8.62E-03	1.70E-02	6.13E-01	-8.03E-03	5.58E-03	1.50E-01	6.77E-03	5.95E-03	2.55E-01

Estimations were carried out using the random-effects IVW method. Here, 2013 represents the GWAS summary data of birth weight obtained from Horikoshi et al (2013) (Horikoshi et al., 2013), 2016 represents the GWAS summary data of birth weight obtained from Horikoshi et al (2016) (Horikoshi et al., 2016), and UK Biobank represents the GWAS summary data of birth weight available from UK Biobank.

Table S9 Estimated causal effect, Q statistic and statistical power for detecting non-zero causal effects of lower birth weight on various diseases

Traits	Sample Size	Estimated Causal Effect (SE)		I^2(%)	Q	p value of Q	Power		2013
		without pleiotropic instruments	with pleiotropic instruments				Expected	Observed	
2hrGlucose	15,234	-0.07 (0.15)	0.02 (0.16)	32.6	56.4	2.16E-02	0.05	0.02	0.41 (0.30)
Adiponectin	45,891	0.02 (0.04)	0.05 (0.05)	45.5	60.6	1.67E-03	0.33	0.00	-0.04 (0.08)
AgeSmoke	74,053	0.02 (0.01)	0.02 (0.01)	0.0	31.4	7.65E-01	0.63	0.01	0.02 (0.02)
Birth Length	28,459	0.01 (0.13)	0.34 (0.13)	68.2	94.3	7.70E-09	0.15	0.01	0.18 (0.32)
BMI	339,224	-0.05 (0.03)	-0.07 (0.04)	20.9	45.5	1.10E-01	1.00	0.72	-0.13 (0.03)
BMI_Child	35,668	-0.02 (0.07)	-0.04 (0.07)	39.3	62.6	5.36E-03	0.22	0.00	0.06 (0.14)
Body Fat	100, 716	-0.03 (0.04)	-0.06 (0.04)	37.4	60.7	8.26E-03	0.82	0.02	-0.05 (0.08)
CigsPerDay	74,053	-0.62 (0.52)	-0.63 (0.52)	0.0	29.0	8.54E-01	0.63	1.00	0.09 (0.66)
College	126,559	-0.06 (0.07)	-0.06 (0.07)	28.6	53.3	4.07E-02	0.92	0.33	-0.10 (0.11)
EduYears	126,559	-0.02 (0.03)	-0.02 (0.03)	21.4	48.3	1.01E-01	0.92	0.01	-0.04 (0.06)
EverSmoke	74,053	-0.06 (0.08)	-0.06 (0.08)	0.0	33.0	6.99E-01	0.63	0.14	-0.08 (0.09)
Fasting Insulin	46,186	0.00 (0.03)	0.00 (0.03)	26.9	53.3	5.03E-02	0.34	0.00	0.01 (0.05)
Fasting Glucose	46,186	0.04 (0.03)	0.07 (0.03)	36.2	58.0	1.16E-02	0.34	0.02	0.15 (0.05)
FormerSmoke	74,053	-0.02 (0.11)	-0.02 (0.11)	15.5	46.1	1.71E-01	0.63	0.01	-0.15 (0.13)
Growth_10_12	13,960	-0.11 (0.11)	-0.11 (0.11)	15.1	21.19	2.18E-01	0.05	0.06	-0.22 (0.22)
Growth_PG	10,799	0.09 (0.10)	0.14 (0.11)	34.0	57.5	1.68E-02	0.03	0.02	-0.09 (0.13)
Growth_PT	9,228	0.15 (0.14)	0.15 (0.13)	0.0	29.1	8.50E-01	0.02	0.09	-0.05 (0.18)
Head Circumference	10,678	0.02 (0.10)	-0.06 (0.12)	21.1	46.9	1.05E-01	0.03	0.00	-0.40 (0.19)
Height	253,288	-0.01 (0.04)	-0.02 (0.11)	64.1	69.6	2.54E-06	1.00	0.01	-0.31 (0.22)
High Density Lipoproteins	97,749	-0.02 (0.04)	-0.02 (0.05)	32.7	54.9	2.25E-02	0.80	0.01	-0.02 (0.08)
Hip Circumference	224,459	-0.05 (0.04)	-0.06 (0.05)	48.4	65.8	5.84E-04	1.00	0.45	-0.20 (0.06)
HOMA_B	46,186	-0.02 (0.03)	-0.02 (0.03)	46.2	72.5	6.24E-04	0.34	0.00	-0.05 (0.05)
HOMA_IR	46,186	0.01 (0.03)	0.01 (0.03)	17.2	47.1	1.48E-01	0.34	0.00	0.03 (0.04)

Insulin Sensitivity Index	16,753	-0.02 (0.08)	-0.02 (0.08)	24.4	51.6	6.95E-02	0.06	0.00	-0.14 (0.09)
Leptin	32,161	0.01 (0.05)	-0.04 (0.05)	22.1	48.8	9.26E-02	0.19	0.00	-0.09 (0.09)
Low Density Lipoproteins	93,354	0.07 (0.04)	0.09 (0.04)	23.6	49.7	7.86E-02	0.78	0.34	-0.00 (0.04)
Obesity	263,407	-0.43 (0.25)	-0.43 (0.25)	15.8	46.3	1.66E-01	1.00	1.00	1.03 (0.29)
Obesity_Child	13,848	0.27 (0.20)	0.27 (0.20)	31.7	57.1	2.42E-02	0.05	0.78	-0.21 (0.23)
Overweight	263,407	-0.06 (0.06)	-0.03 (0.06)	0.0	36.5	4.90E-01	1.00	0.79	-0.16 (0.07)
Serum Urate	110,347	0.04 (0.05)	0.08 (0.05)	50.1	74.1	1.89E-04	0.86	0.27	0.19 (0.05)
Tanner	9,916	-0.13 (0.10)	-0.13 (0.10)	21.4	45.8	1.05E-01	0.03	0.06	0.02 (0.15)
Total Cholesterol	100,184	0.05 (0.04)	0.08 (0.05)	35.2	57.1	1.42E-02	0.82	0.13	-0.00 (0.07)
Triglycerides	94,461	0.05 (0.04)	0.05 (0.04)	37.5	62.4	7.49E-03	0.78	0.11	-0.01 (0.05)
Waist Circumference	224,459	-0.05 (0.04)	-0.10 (0.04)	43.1	59.8	2.91E-03	1.00	0.45	-0.22 (0.03)
Waist-to-hip ratio	224,459	-0.04 (0.04)	-0.08 (0.04)	50.3	74.5	1.70E-04	1.00	0.23	-0.15 (0.06)
Weight	6,950	-0.36 (0.12)	-0.36 (0.12)	36.6	72.6	5.72E-03	0.02	0.67	-0.49 (0.21)
Diastolic Blood Pressure	8,749	0.27 (0.23)	0.27 (0.23)	0.0	16.04	0.99	0.02	0.48	0.38 (0.25)
Systolic Blood Pressure	8,749	0.35 (0.23)	0.35 (0.23)	0.0	6.83	0.99	0.02	0.78	0.50 (0.25)

The sources of the sumamry data for these complex traits are shown in Text S2 and Text S5. SE in parentheses is the standard error of the estimated causal effect for birth weight using the random-effects IVW method. The causal effects estimated with or without potentially pleiotropic instruments for all diseases are largely similar (note that estimates for certain diseases are identical in the two cases as no pleiotropic instruments were identified for these diseases). In our analyses we mainly focus on the estimates obtained without potentially pleiotropic instruments. I^2 and Q are two statistics for the heterogeneity test. Statistical power (last two columns) is computed using the method given in (Brion et al., 2013) by setting PVE = 1.7% and the significance level α to 1.28E-3 (=0.05/38) and ignoring uncertainty. The expected power is computed by assuming the causal effect of birth weight on disease is ±0.10, while the observed power is computed based on the estimated causal effect for each disease. The power was calculated by using the method presented in (Brion et al., 2013) that is implemented online at https://cnsgenomics.shinyapps.io/mRnd/. 2013 in the last column represents the estimates of causal effects for these diseases using the seven instruments obtained from Horikoshi et al (2013) (Horikoshi et al., 2013).

Table S10 Estimated direct and indirect causal effects of lower birth weight on four discovered diseases using multivariable Mendelian randomisation regression

Traits	Direct Causal Effect (SE)				Indirect Causal Effect (SE)			
	CAD	MI	T2D	T2D_BMI	CAD	MI	T2D	T2D_BMI
2hrGlucose	1.43 (0.08)	1.43 (0.09)	1.86 (0.17)	2.05 (0.18)	0.93 (0.11)	0.91 (0.10)	0.76 (0.2)	0.75 (0.21)
Adiponectin	1.38 (0.09)	1.36 (0.09)	1.60 (0.16)	1.73 (0.16)	0.97 (0.11)	0.95 (0.10)	0.88 (0.19)	0.89 (0.19)
AgeSmoke	1.38 (0.08)	1.38 (0.09)	1.73 (0.18)	1.88 (0.19)	0.97 (0.11)	0.95 (0.10)	0.82 (0.21)	0.82 (0.22)
Birth Length	1.35 (0.08)	1.34 (0.09)	1.88 (0.18)	2.03 (0.19)	1.00 (0.11)	0.98 (0.10)	0.75 (0.21)	0.76 (0.22)
BMI	1.32 (0.08)	1.31 (0.09)	1.63 (0.17)	1.75 (0.18)	1.01 (0.11)	0.99 (0.10)	0.86 (0.21)	0.88 (0.21)
BMI_Child	1.43 (0.08)	1.42 (0.09)	1.82 (0.18)	1.99 (0.19)	0.93 (0.11)	0.91 (0.10)	0.78 (0.21)	0.78 (0.22)
Body Fat	1.42 (0.08)	1.42 (0.09)	1.80 (0.18)	1.99 (0.19)	0.95 (0.11)	0.91 (0.10)	0.78 (0.21)	0.77 (0.22)
CigsPerDay	1.40 (0.08)	1.39 (0.09)	1.82 (0.18)	2.01 (0.19)	0.95 (0.11)	0.93 (0.10)	0.77 (0.21)	0.77 (0.22)
College	1.42 (0.08)	1.42 (0.09)	1.90 (0.18)	2.10 (0.19)	0.94 (0.11)	0.91 (0.10)	0.75 (0.21)	0.74 (0.22)
EduYears	1.43 (0.08)	1.43 (0.09)	1.88 (0.18)	2.08 (0.19)	0.94 (0.11)	0.91 (0.10)	0.76 (0.21)	0.75 (0.22)
EverSmoke	1.43 (0.08)	1.43 (0.09)	1.86 (0.18)	2.05 (0.19)	0.93 (0.11)	0.90 (0.10)	0.76 (0.21)	0.75 (0.22)
Fasting Insulin	1.43 (0.08)	1.42 (0.09)	1.97 (0.16)	2.23 (0.17)	0.93 (0.11)	0.91 (0.10)	0.71 (0.20)	0.69 (0.20)
Fasting Glucose	1.43 (0.08)	1.42 (0.09)	1.54 (0.16)	1.70 (0.17)	0.94 (0.11)	0.91 (0.10)	0.91 (0.19)	0.90 (0.20)
FormerSmoke	1.43 (0.08)	1.43 (0.09)	1.90 (0.18)	2.10 (0.19)	0.93 (0.11)	0.90 (0.09)	0.75 (0.21)	0.73 (0.22)
Growth_PG	1.43 (0.08)	1.43 (0.09)	1.86 (0.18)	2.08 (0.19)	0.94 (0.11)	0.90 (0.10)	0.76 (0.21)	0.74 (0.22)
Growth_PT	1.43 (0.08)	1.43 (0.09)	1.80 (0.18)	2.01 (0.19)	0.93 (0.11)	0.90 (0.10)	0.79 (0.21)	0.76 (0.22)
Head Circumference	1.42 (0.08)	1.42 (0.09)	1.88 (0.18)	2.10 (0.19)	0.94 (0.11)	0.91 (0.10)	0.75 (0.21)	0.73 (0.22)
Height	1.42 (0.08)	1.42 (0.09)	1.90 (0.18)	2.10 (0.19)	0.94 (0.11)	0.91 (0.10)	0.75 (0.21)	0.73 (0.22)
High Density Lipoproteins	1.38 (0.08)	1.36 (0.09)	1.82 (0.18)	1.99 (0.19)	0.97 (0.11)	0.95 (0.10)	0.77 (0.21)	0.77 (0.22)
Hip Circumference	1.39 (0.08)	1.38 (0.09)	1.80 (0.18)	1.95 (0.19)	0.97 (0.11)	0.94 (0.10)	0.79 (0.21)	0.79 (0.22)
HOMA_B	1.42 (0.08)	1.42 (0.09)	1.86 (0.15)	2.08 (0.16)	0.94 (0.11)	0.91 (0.10)	0.76 (0.19)	0.74 (0.20)
HOMA_IR	1.45 (0.08)	1.42 (0.09)	1.99 (0.17)	2.25 (0.18)	0.93 (0.11)	0.91 (0.10)	0.70 (0.20)	0.68 (0.21)
Insulin Sensitivity Index	1.42 (0.08)	1.42 (0.09)	1.86 (0.17)	2.08 (0.18)	0.94 (0.11)	0.91 (0.10)	0.76 (0.20)	0.74 (0.21)

Leptin	1.42 (0.08)	1.42 (0.09)	1.92 (0.17)	2.12 (0.19)	0.95 (0.11)	0.92 (0.10)	0.73 (0.21)	0.73 (0.22)
Low Density Lipoproteins	1.40 (0.08)	1.42 (0.09)	1.82 (0.18)	2.03 (0.19)	0.96 (0.11)	0.91 (0.10)	0.77 (0.21)	0.76 (0.22)
Obesity	1.39 (0.08)	1.38 (0.09)	1.75 (0.18)	1.90 (0.19)	0.96 (0.11)	0.94 (0.10)	0.80 (0.21)	0.81 (0.22)
Obesity_Child	1.42 (0.08)	1.42 (0.09)	1.84 (0.18)	2.08 (0.19)	0.94 (0.11)	0.91 (0.10)	0.76 (0.21)	0.75 (0.22)
Overweight	1.42 (0.08)	1.42 (0.09)	1.86 (0.17)	2.05 (0.19)	0.94 (0.11)	0.91 (0.10)	0.76 (0.21)	0.75 (0.22)
Serum Urate	1.42 (0.08)	1.43 (0.09)	1.90 (0.18)	2.10 (0.19)	0.94 (0.11)	0.90 (0.10)	0.74 (0.21)	0.74 (0.22)
Tanner	1.40 (0.09)	1.40 (0.09)	1.65 (0.15)	1.84 (0.15)	0.95 (0.11)	0.92 (0.10)	0.85 (0.19)	0.84 (0.19)
Total Cholesterol	1.42 (0.08)	1.45 (0.09)	1.84 (0.18)	2.05 (0.19)	0.94 (0.11)	0.90 (0.10)	0.77 (0.21)	0.75 (0.22)
Triglycerides	1.40 (0.08)	1.40 (0.09)	1.79 (0.17)	1.97 (0.19)	0.95 (0.11)	0.92 (0.10)	0.79 (0.21)	0.79 (0.22)
Waist Circumference	1.38 (0.08)	1.38 (0.09)	1.77 (0.18)	1.92 (0.19)	0.97 (0.11)	0.94 (0.10)	0.79 (0.21)	0.80 (0.22)
Waist-to-hip ratio	1.42 (0.08)	1.42 (0.09)	1.88 (0.18)	2.08 (0.19)	0.94 (0.11)	0.91 (0.10)	0.75 (0.21)	0.74 (0.22)
Weight	1.27 (0.08)	1.27 (0.09)	1.67 (0.18)	1.73 (0.19)	1.05 (0.11)	1.02 (0.10)	0.84 (0.21)	0.90 (0.22)
Hypertension	1.42 (0.08)	1.42 (0.09)	1.90 (0.17)	2.10 (0.19)	0.94 (0.11)	0.91 (0.10)	0.75 (0.21)	0.73 (0.22)
Systolic Blood Pressure	1.55 (0.24)	1.48 (0.25)	2.44 (0.79)	2.39 (0.89)	0.86 (0.25)	0.89 (0.25)	0.58 (0.80)	0.65 (0.90)
Diastolic Blood Pressure	1.23 (0.27)	1.17 (0.28)	2.27 (0.86)	2.46 (0.99)	1.08 (0.28)	1.11 (0.28)	0.63 (0.87)	0.63 (0.99)

The sources of the sumamry data for the four diseases and complex traits are shown in Text S2 and Text S5. SE in parentheses is the standard error of the estimated causal effect for birth weight.

Table S11 Studies used in the GWAS of T2D (Scott et al., 2017)

Studies	Sample Size (Case/Control)
ARIC: The Atherosclerosis Risk in Communities	7,764 (755/7,009)
BioMe: MT. SINAI BioMe Biobank Platform	2,489 (387/2,102)
deCODE: deCODE genetics	90,388 (7,339/83,049)
DGDG: Diabetes Gene Discovery Group	1,376 (679/697)
DGI: Diabetes Genetics Initiative	2,097 (1,023/1,074)
EGCUT-370: Estonian Genome Center, University of Tartu	1,848 (80/1,768)
EGCUT-OMNI: Estonian Genome Center, University of Tartu	6,402 (389/6,013)
EPIC: The European Prospective Investigation into Cancer and Nutrition	9,292 (4,624/4,668)
FHS: Framingham Heart Study	8,333 (673/7,660)
FUSION: Finland-United States Investigation of NIDDM Genetics	2,335 (1,161/1,174)
GoDARTS: Genetics of Diabetes and Audit Research in Tayside Scotland	5,941 (3,298/2,643)
HPFS: HSPH, Health Professionals Follow-up Study	2,422 (1,124/1,298)
KORAgen: KORAgen Study Helmholtz zentrum München	1,897 (347/1,550)
NHS: HSPH, Nurses' Health Study	3,221 (1,467/1,754)
PIVUS: Prospective Investigation of the Vasculature in Uppsala Seniors	949 (111/838)
RS-I: Rotterdam Study	5,873 (654/5,219)
ULSAM: Uppsala Longitudinal Study of Adult Men	1,119 (166/953)
WTCCC: Wellcome Trust Case-Control Consortium	4,862 (1,924/2,938)
**Total**	158,608 (26,201/132,407)

The summary data of T2D can be available from http://diagram-consortium.org/downloads.html. The total sample size (i.e. 158,608) is slightly different from that (i.e. 159,208) in Text S2; this is mainly due to quality control of the data.

Table S12 Studies used in the CARDIoGRAMplusC4D Consortium for CAD and MI (Nikpay et al., 2015)

Studies	Sample Size (Case/Control)
PROCARDIS: The Precocious Coronary Artery Disease	12,264 (5,719/6,545)
HSDS: The Helsinki Sudden Death Study/Tampere Coronary Study	465 (206/259)
ADVANCE: Atherosclerotic Disease, VAscular functioN, and genetiC Epidemiology	590 (278/312)
BEIJING (BAS): The Beijing Atherosclerosis Study	1,526 (505/1,021)
CARDIOGENICS: The CARDIOGENICS Study	802 (392/410)
CHINA (CAS): The China Atherosclerosis Study	50,08 (1,010/3,998)
CCGB_2: The Cleveland Clinic Gene Bank	1,996 (1,628/368)
COROGENE: The Corogene Study	4,131 (2,083/2,048)
DUKE_2: The Duke Cathgen Study	1,869 (1,216/653)
EGCUT: Estonian Genome Center of University of Tartu	6,499 (658/5,841)
FGENTCARD: Functional genomic diagnostic tools for coronary artery disease	2,268 (1,802/466)
GENRIC: Genomics Research in Cardiovascular Disease	4,789 (2,099/2,690)
GERMIFS I: The German Myocardial Infarction Family Study I	2,242 (634/1,608)
GERMIFS II: The German Myocardial Infarction Family Study II	2,495 (1,207/1,288)
GERMIFS III (KORA): The German Myocardial Infarction Family Study III (KORA)	2,528 (1,061/1,467)
GERMIFS_IV: The German Myocardial Infarction Family Study IV	2,236 (1,089/1,147)
GODARTS: Genetics of Diabetes and Audit Research in Tayside Scotland	877 (8,77/0)
HPS: The MRC/BHF Heart Protection Study	5,458 (2,700/2,758)
IPM_AA: The MT. SINAI BioMe Biobank Platform	3139 (361/2,778)
IPM_EA: The MT. SINAI BioMe Biobank Platform	1,868 (487/1,381)
IPM_HA The: MT. SINAI BioMe Biobank Platform	4,095 (758/3,337)
LOLIPOP: The London Life Sciences Prospective Population Study	6,548 (2,791/3,757)
LURIC: The Ludwigshafen Risk and Cardiovascular Health Study	2,598 (2,095/503)
MEDSTAR: The Medstar cardiac catheterization Study	1,401 (933/468)
MIGen: The Myocardial Infarction Genetics Consortium	5,903 (2,905/2,998)
OHGS_A2: The Ottawa Heart Genetic Study A2	1,955 (947/1,008)
OHGS_B2: The Ottawa Heart Genetic Study B2	2,823 (1,294/1,529)

OHGS_C2: The Ottawa Heart Genetic Study C2	1,161 (843/318)
PENNCATH: University of Pennsylvania Medical Cente cardiac catherization study	1,401 (933/468)
PIVUS: The Prospective Investigation of the Vasculature in Uppsala Seniors	119 (119/0)
PREDICTCVD: A subset of FINRISK cohort study	965 (631/334)
SDS/AIDHS: Sikh Diabetes Study/ Asian Indian Diabetic Heart Study	836 (836/0)
THISEAS: The Hellenic Study of Interactions between SNPS and Eating in Atherosclerosis Susceptibility	1,020 (426/594)
TWINGENE: TWINGENE	814 (814/0)
ULSAM: The Uppsala Longitudinal Study of Adult Men	322 (322/0)
WTCCC: The Wellcome Trust Case Control Consortium	4,864 (1,926/2,938)
PROMIS1: The Pakistan Risk of Myocardial Infarction Study	9,103 (4,651/4,452)
PROMIS2: The Pakistan Risk of Myocardial Infarction Study	8,309 (4,380/3,929)
LIFE-HEART: Leipzig Research Center for Civilization Diseases - Heart Study	2,307 (1,535/772)
WGHS: The Women's Genome Health Study	23,293 (1,007/22,286)
ITH_2: The INTERHEART study	850 (402/448)
MAYO-VDB: The case-control study	745 (745/0)
AGES: The Age, Gene/Environment Susceptibility–Reykjavik Study	2,871 (397/2,474)
RS: The Rotterdam Study	5,841 (506/5,335)
FHS: The Framingham Heart Study	4,461 (259/4,202)
FamHS: The Family Heart Study	3,780 (334/3,446)
PROSPER: PROspective Study of Pravastatin in the Elderly at Risk	5,244 (2,034/3,210)
ARIC: Atherosclerosis Risk in Communities	8,897 (454/8,443)
**Total**	175,576 (61,289/114,287)

The summary data of CAD and MI can be available from http://www.cardiogramplusc4d.org/data-downloads/. The total sample size (i.e. 175,576) is slightly different from that (i.e. 184,305) in Text S2; this is mainly due to quality control of the data.

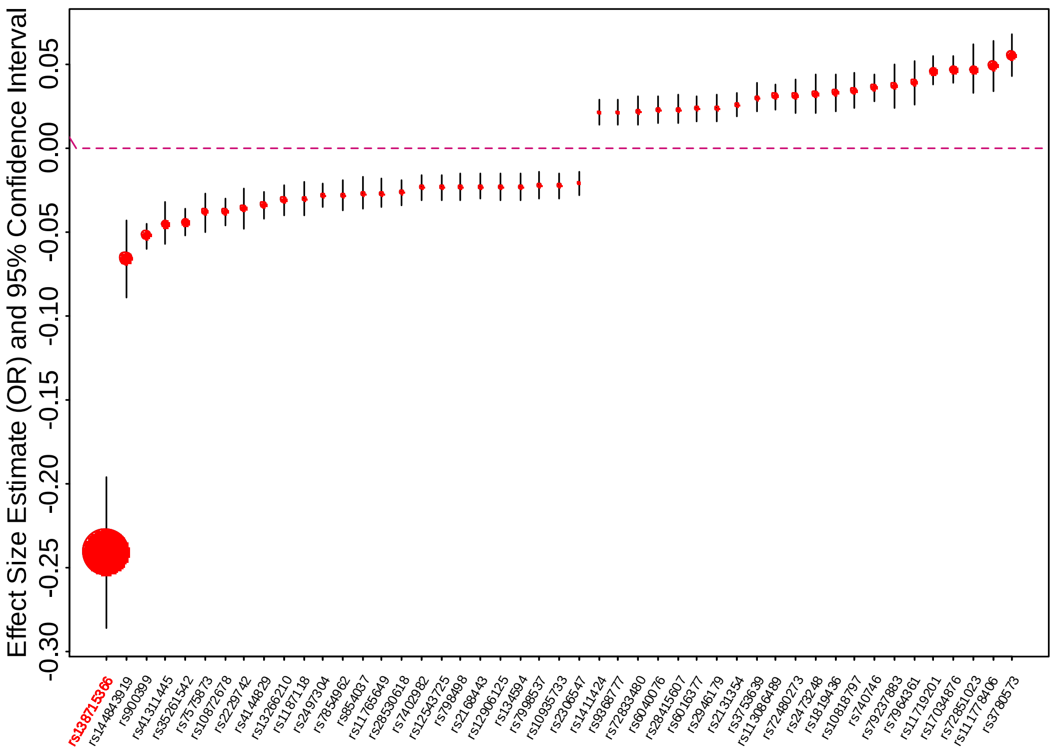

FIGURE S1 SNP effect size estimate on birth weight and its 95% confidence interval for each of the 47 SNPs that serve as instrumental variables for birth weight. SNP ids (x-axis) are ordered based on their effect size estimates, where the first SNP rs138715366 (red) has the largest absolute effect size estimate (β = -0.24, se = 0.02 and p = 7.20E-26). The size of the dots is proportional to the absolute effect size estimate. The summary data of instruments for birth weight can be available from http://egg-consortium.org/

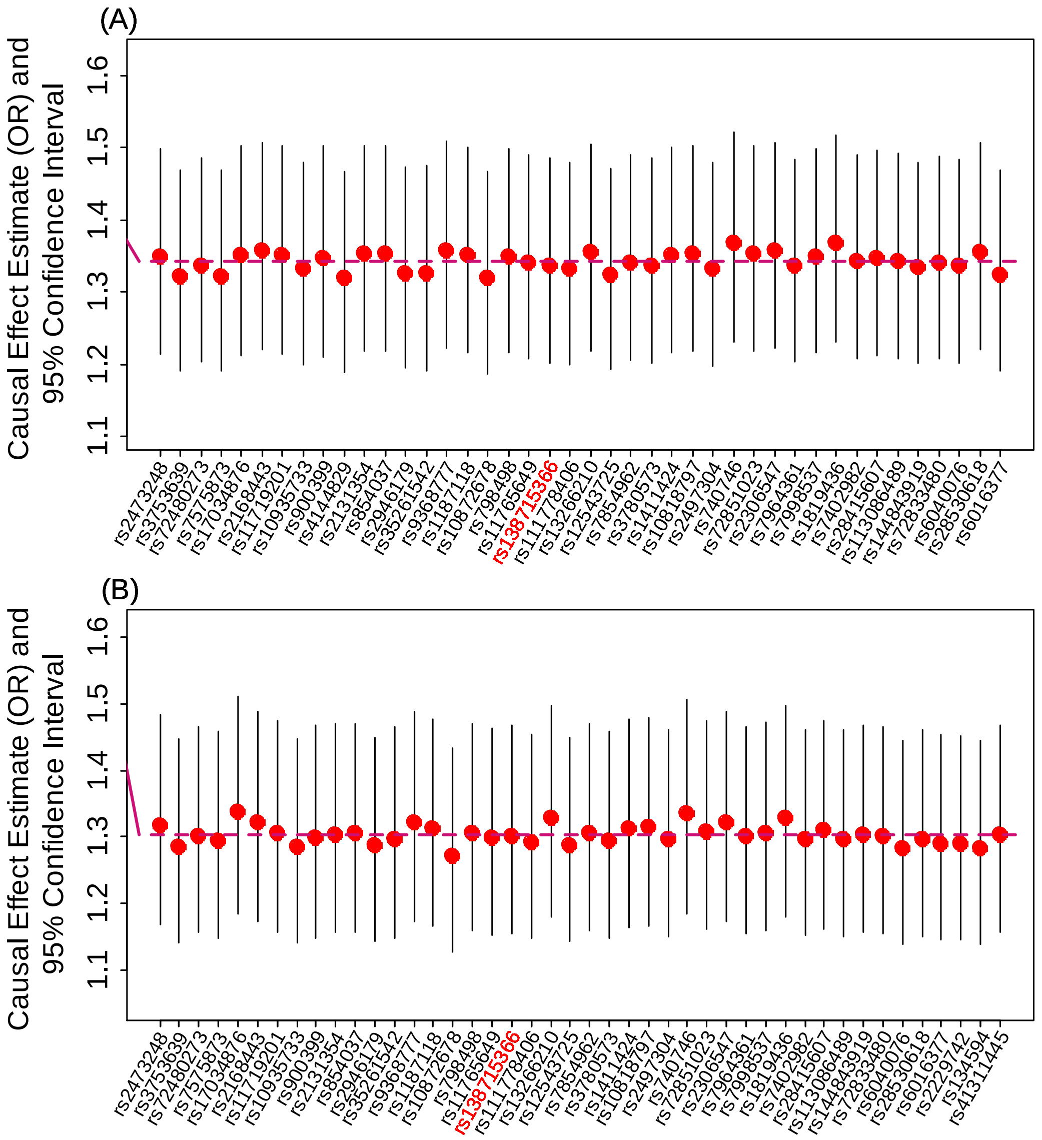
(A)
(B)
Causal Effect Estimate (OR) and
95% Confidence Interval
1.1
1.2
1.3
1.4
1.5
1.6
rs138715366

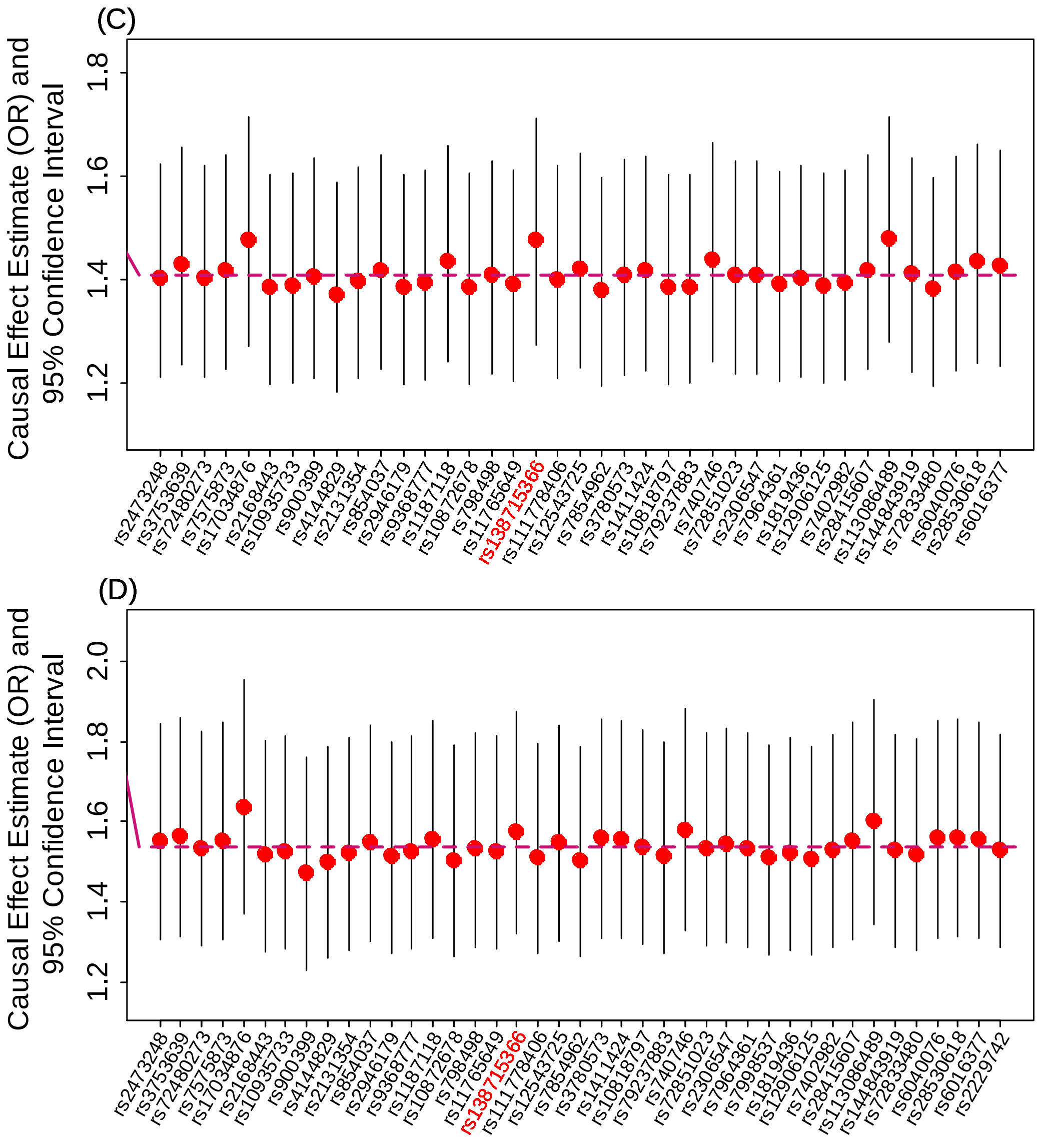

FIGURE S2 Estimated causal effects and 95% confidence intervals for lower birth weight on diseases in the leave-one-out analysis by omitting one SNP (x-axis) at a time. Estimation is carried out using the random-effects inverse-variance weighted method. Examined diseases include (**A**) CAD, (**B**) MI, (**C**) T2D, and (**D**) T2D_BMI. rs138715366, which appears to be an outlier, is highlighted in red in all panels. The summary data of instruments for birth weight can be available from http://egg-consortium.org/; the summary data of CAD and MI can be available from http://www.cardiogramplusc4d.org/data-downloads/; the summary data of T2D can be available from http://diagram-consortium.org/downloads.html

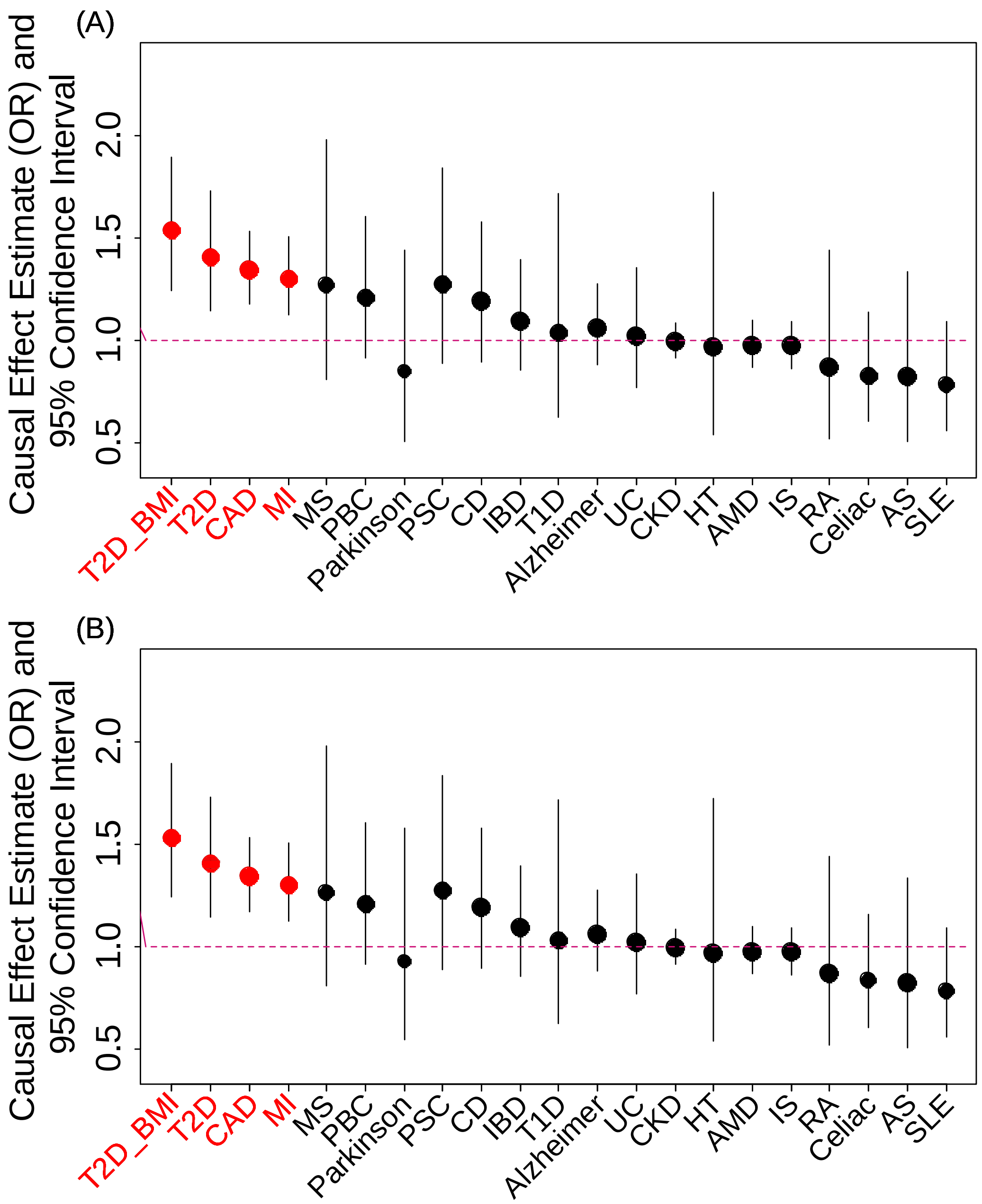
(A)
(B)
Causal Effect Estimate (OR) and 95% Confidence Interval
0.5
1.0
1.5
2.0
T2D_BMI
T2D
CAD
MI
MS
PBC
Parkinson
PSC
CD
IBD
T1D
Alzheimer
UC
CKD
HT
AMD
IS
RA
Celiac
AS
SLE

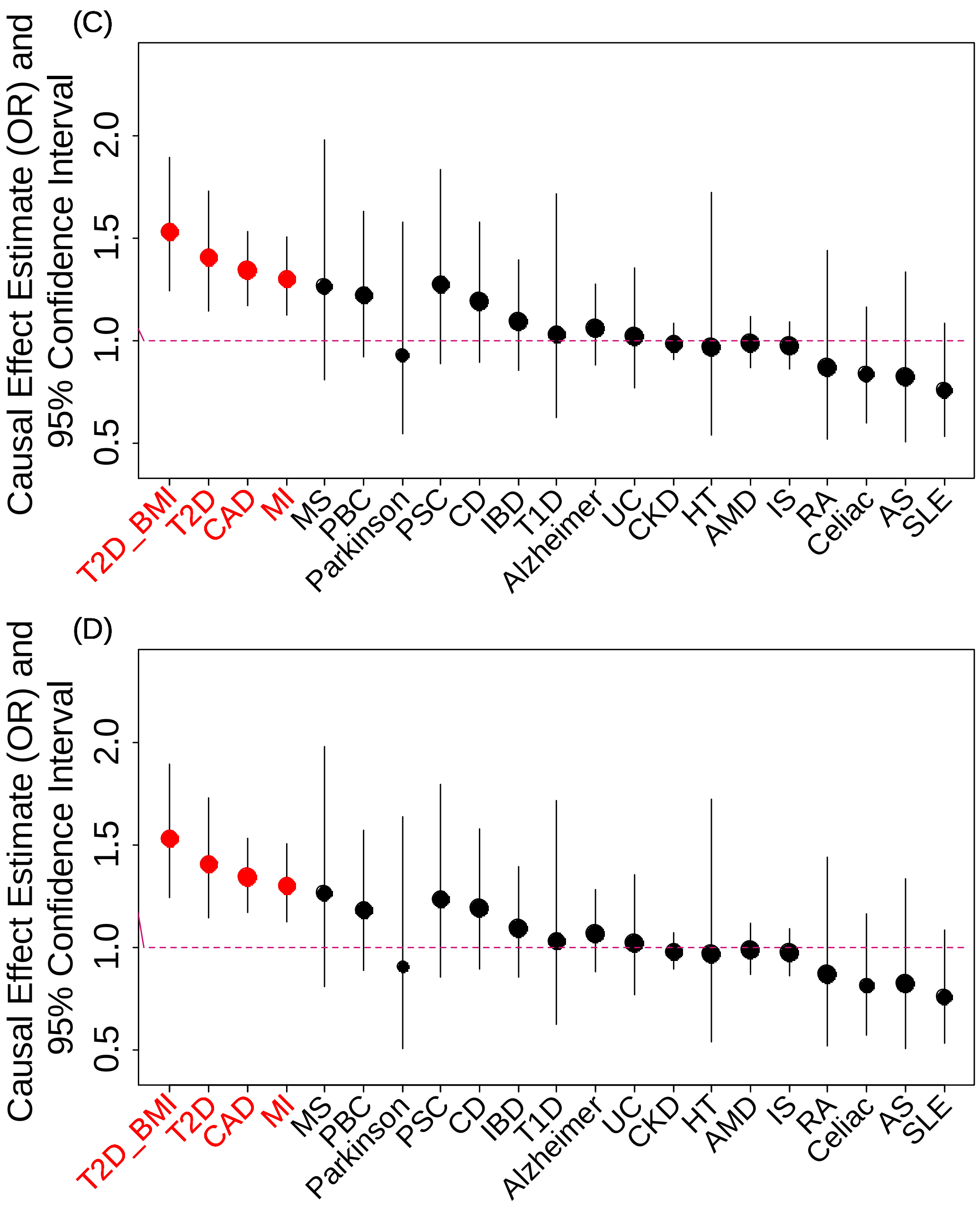

FIGURE S3 Estimated causal effect and 95% confidence intervals for lower birth weight on 21 diseases. Diseases are ordered in the same as those in Fig 2. Estimations are carried out using both index SNPs and proxy SNPs, where the proxy SNPs are obtained using various correlation thresholds: (**A**) $r = 0.5$, (**B**) $r = 0.6$, (**C**) $r = 0.7$, and (**D**) $r = 0.8$. In each panel, the dot size is proportional to the number of instrumental variables used for the given disease while dot color represents significance ($p < 0.05$ are highlighted in red). Disease names (x-axis) are further highlighted in red if the causal effects are significant after Bonferroni correction ($p < 0.05/21$). The summary data of these diseases are shown in Text S1 and Text S5

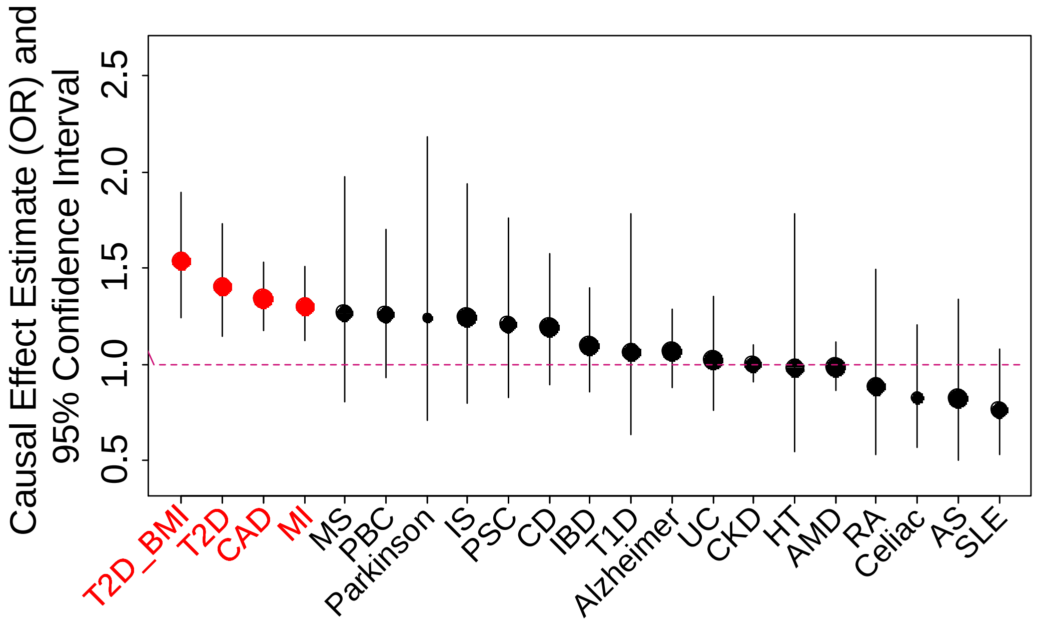

FIGURE S4 Estimated causal effects and 95% confidence intervals for lower birth weight on 21 diseases using the random-effects inverse variance weighted (IVW) method. Diseases are ordered in the same as those in Fig 2. Estimations are carried out using both index and imputed SNPs. The dot size is proportional to the number of instrumental variables used for the given disease while dot color represents significance ($p < 0.05$ are highlighted in red). Disease names (x-axis) are further highlighted in red if the causal effects are significant after Bonferroni correction ($p < 0.05/21$). Additionally, the estimated casual effects of birth weight on CAD, MI, T2D, and T2D_BMI are the same as those presented in the main text as all 47 instrumental variables are available from summary data for the four traits. The summary data of these diseases are shown in Text S1 and Text S5

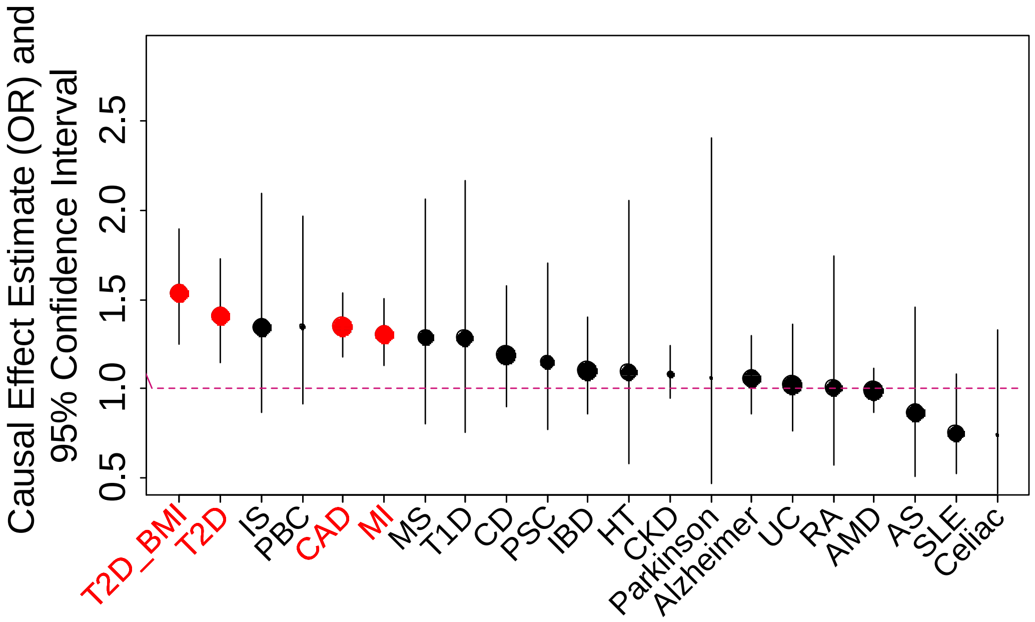

FIGURE S5 Estimated causal effects and 95% confidence intervals for lower birth weight on 21 diseases using the random-effects inverse variance weighted (IVW) method. Diseases are ordered based on their causal effect estimates. Estimations are carried out using only index SNPs. The dot size is proportional to the number of instrumental variables used for the given disease while dot color represents significance ($p < 0.05$ are highlighted in red). Disease names (x-axis) are further highlighted in red if the causal effects are significant after Bonferroni correction ($p <$ 0.05/21). The estimated casual effects of birth weight on CAD, MI, T2D, and T2D_BMI are the same as those presented in the main text as all 47 instrumental variables are available from summary data for the four traits. The summary data of these diseases are shown in Text S1 and Text S5

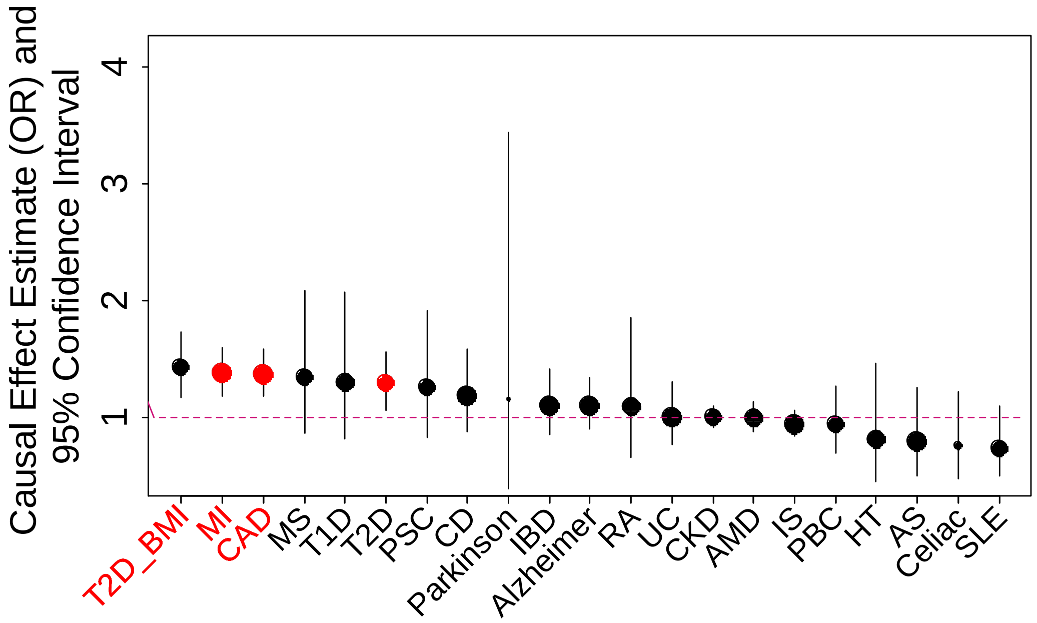

FIGURE S6 Estimated causal effects and 95% confidence intervals for lower birth weight on 21 diseases using the random-effects inverse variance weighted (IVW) method. Diseases are ordered based on their causal effect estimates. Estimations are carried out using both index SNPs and proxy SNPs from the 48 instrumental variables. The dot size is proportional to the number of instrumental variables used for the given disease while dot color represents significance ($p < 0.05$ are highlighted in red). Disease names (x-axis) are further highlighted in red if the causal effects are significant after Bonferroni correction ($p < 0.05/21$). The summary data of these diseases are shown in Text S1 and Text S5

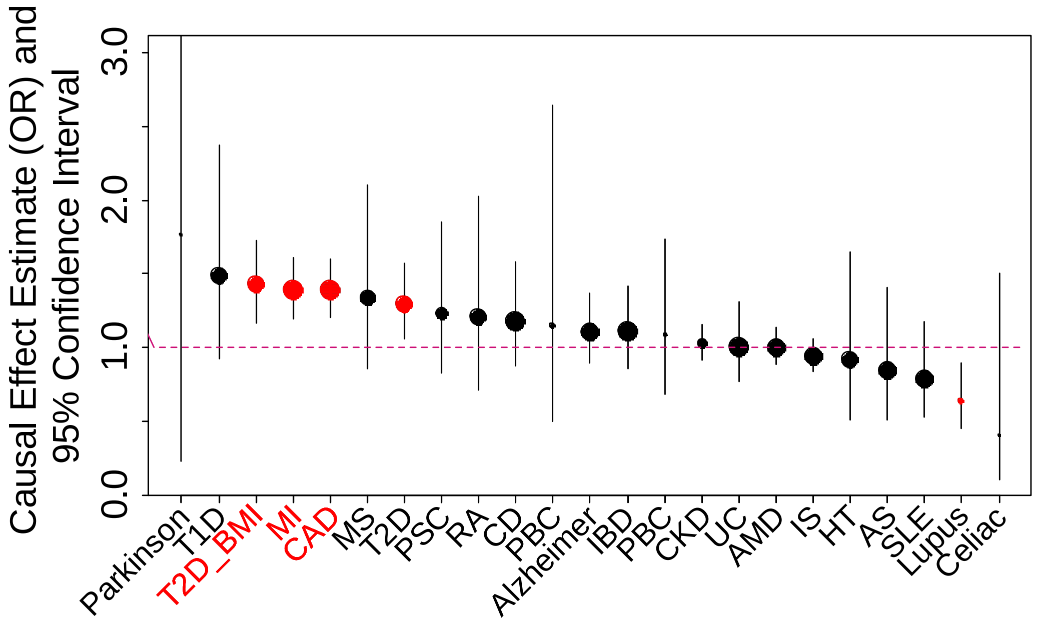

FIGURE S7 Estimated causal effects and 95% confidence intervals for lower birth weight on 21 diseases using the random-effects inverse variance weighted (IVW) method. Diseases are ordered based on their causal effect estimates. Estimations are carried out using only index SNPs from the 48 instrumental variables. The dot size is proportional to the number of instrumental variables used for the given disease while dot color represents significance ($p < 0.05$ are highlighted in red). Disease names (x-axis) are further highlighted in red if the causal effects are significant after Bonferroni correction ($p < 0.05/21$). The estimated casual effects of birth weight on CAD, MI, T2D, and T2D_BMI are unchanged compared to the previous supplementary Figures since all 48 instrumental variables are available from summary data for the four traits. The summary data of these diseases are shown in Text S1 and Text S5

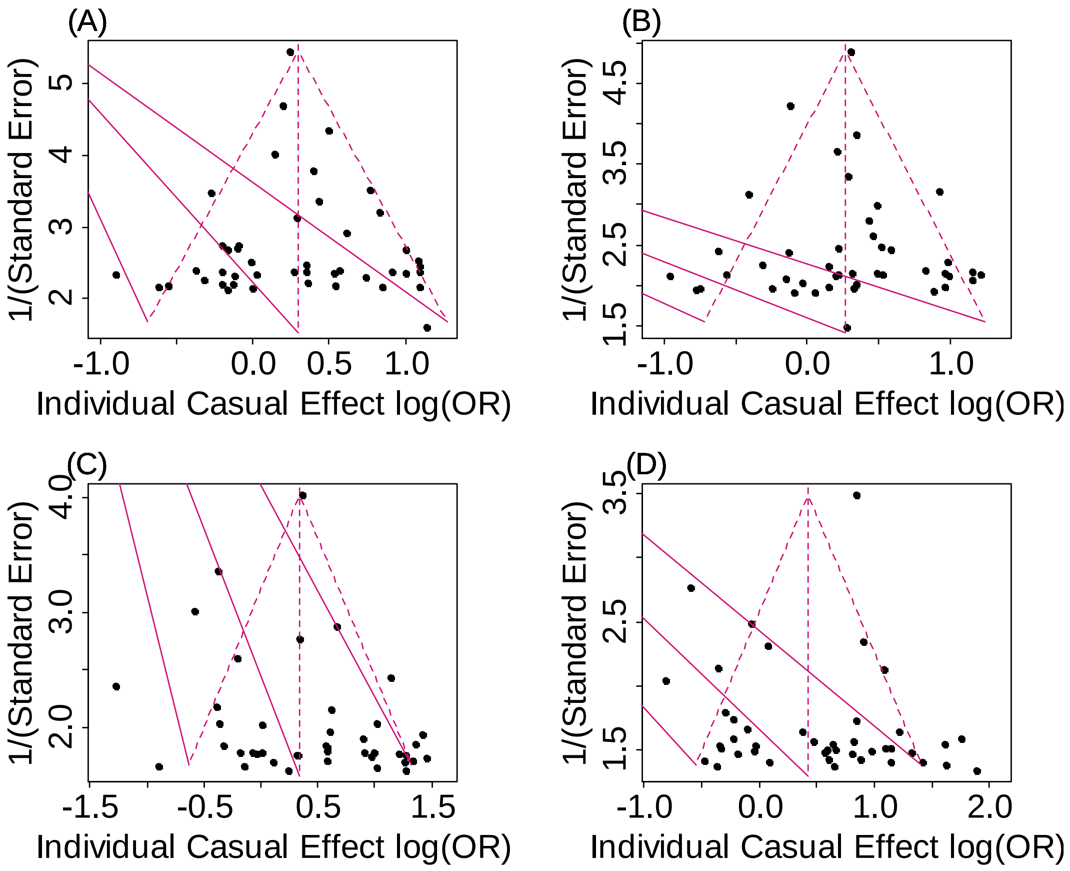

FIGURE S8 Funnel plot displays individual causal effect estimates for lower birth weight on (**A**) CAD, (**B**) MI, (**C**) T2D, and (**D**) T2D_BMI. Center of the dots represents the estimated causal effect for each instrumental variable while the size of the dots is proportional to the absolute value of the causal effect estimate. In each panel, the vertical dotted red lines represent the estimated causal effect obtained using all instrumental variables, while the other two dot lines represent a departure from the overall estimated causal effect within one unit. The summary data of instruments for birth weight can be available from http://egg-consortium.org/; the summary data of CAD and MI can be available from http://www.cardiogramplusc4d.org/data-downloads/; the summary data of T2D can be available from http://diagram-consortium.org/downloads.html

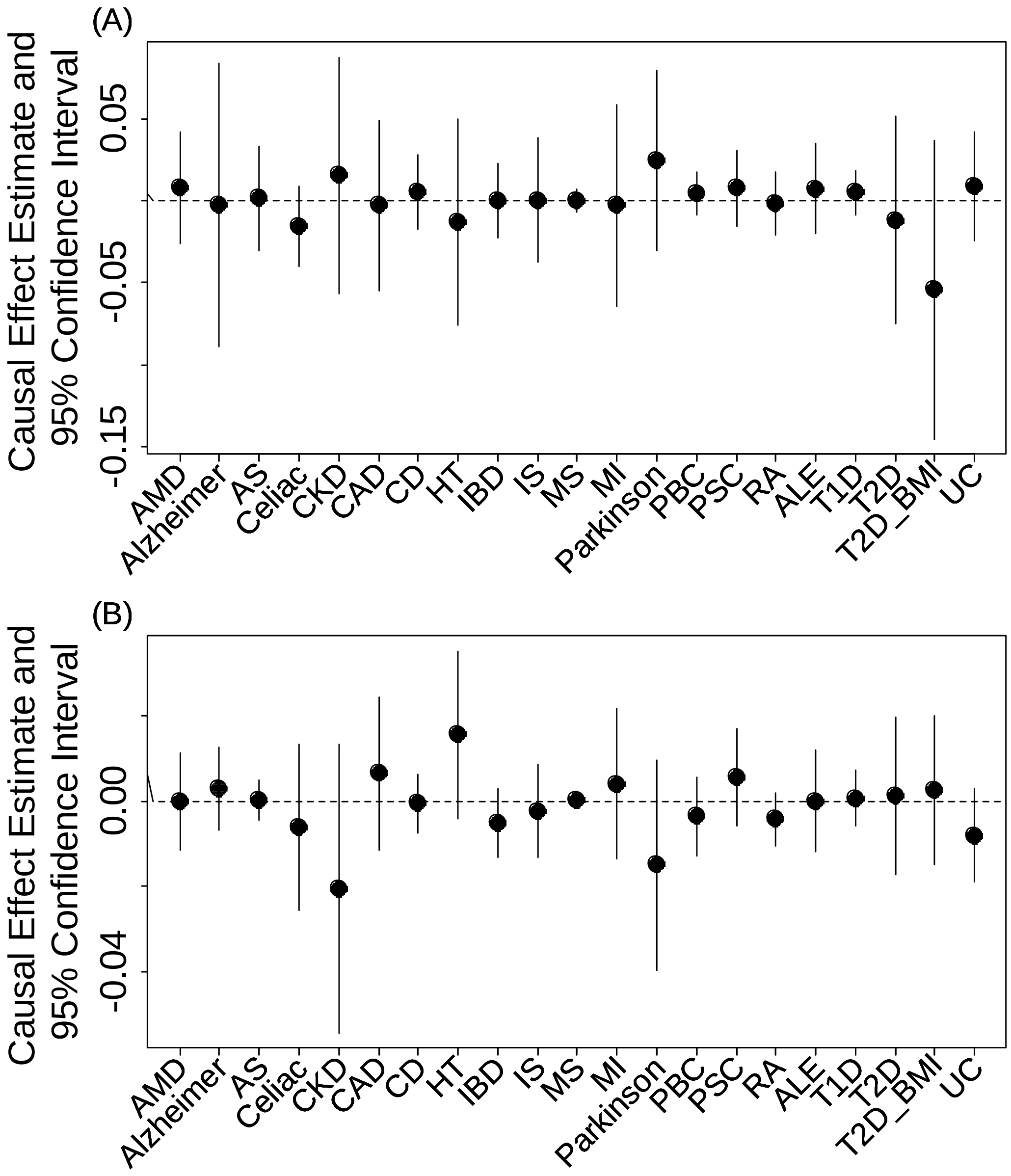

FIGURE S10 Estimated causal effects for the four diseases on birth weight in the reverse causation analysis. Estimations are carried out using the random-effects IVW method. (**A**) The results for birth weight with summary data obtained from Horikoshi et al (2013) (Horikoshi et al., 2013); (**B**) The results for birth weight with summary data obtained from Horikoshi et al (2016) (Horikoshi et al., 2016). Estimated causal effects are on linear regression scale as birth weight is a quantitative trait. The horizontal black line represents zero. The summary data of instruments for birth weight can be available from http://egg-consortium.org/. The GWAS summary data source to these adult diseases is given in Text S5

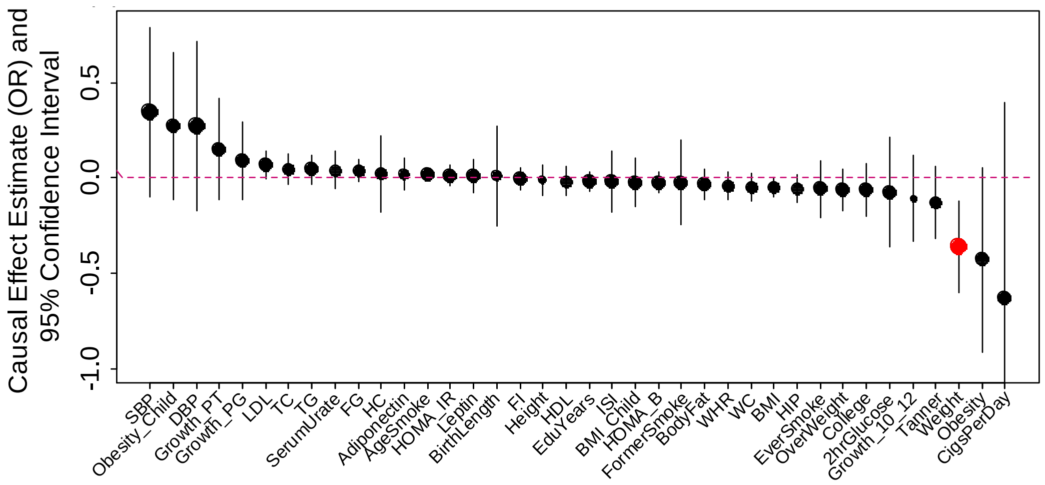

FIGURE S11 Estimated causal effects and 95% confidence intervals of lower birth weight on 38 complex traits. Traits are ordered based on their causal effect estimates. Estimations are carried out using the random-effects inverse variance weighted (IVW) method with both index SNPs and proxy SNPs. Estimated causal effects are on linear regression scale as most traits are quantitative. The dot size is proportional to the number of the instrumental variables used for the trait and dot color represents significance (p < 0.05 are highlighted in red). Trait names (x-axis) are further highlighted in red if they are significant after Bonferroni correction (p < 0.05/38). The horizontal red dotted line represents zero. The summary data of instruments for birth weight can be available from http://egg-consortium.org/; the sources of the summary data for these complex traits are described in Text S2 and Text S5

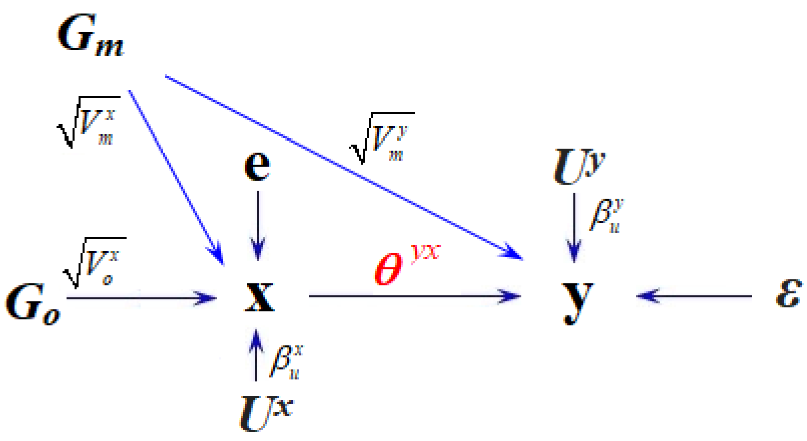

FIGURE S12 Illustration of the simulations performed to evaluate the extent to which maternal effects may influence the estimation of birth weight effect on outcome. Here, **x** is the exposure of interest (i.e. birth weight); G_o and G_m are the instruments for offspring and mother with effect sizes $\sqrt{V_o^x}$ and $\sqrt{V_m^x}$, respectively; **y** is the outcome; ***e*** and $\boldsymbol{\varepsilon}$ are the residual errors; U^x and U^y are the confounders with effect sizes β_u^x and β_u^y; $\sqrt{V_m^y}$ is the effect sizes of G_m on **y**. More details can be found in Text S4

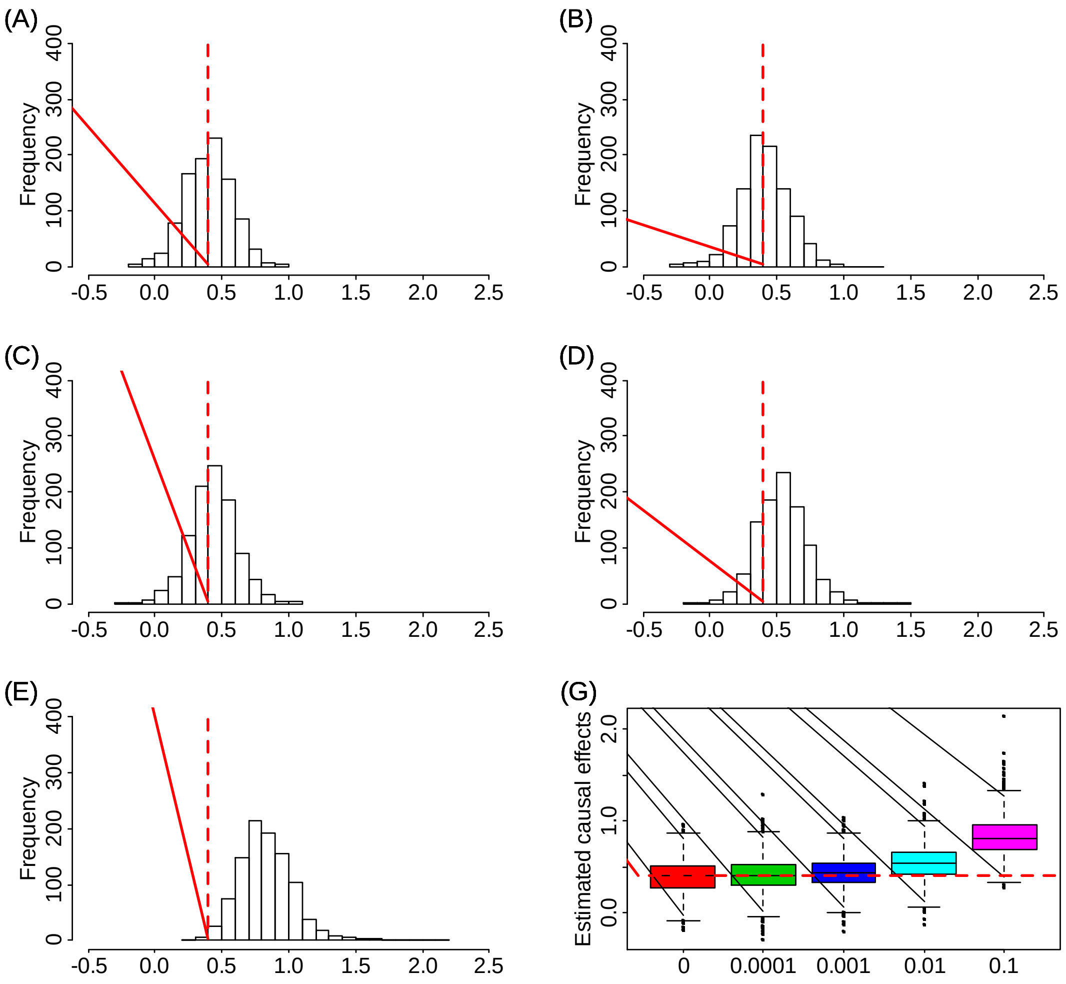

FIGURE S13 Birth-weight causal effect estimates in simulations as illustrated in the previous figure. Histograms display birth weight causal effects estimates across simulation replicates when the maternal effect V_m^y varies from 0 (**A**), 10^{-4} (**B**), 10^{-3} (**C**), 10^{-2} (**D**), to 10^{-1} (**E**). Panel **G** is a boxplot displaying the same results where the true birth weight causal effect is represented as a dotted red horizontal line. As expected, the bias of the estimates becomes larger with increasing maternal effects. 1,000 replicates are simulated in each simulation scenario

References

1. Beaumont, R.N., Warrington, N.M., Cavadino, A., Tyrrell, J., Nodzenski, M., Horikoshi, M., et al. (2018). Genome-wide association study of offspring birth weight in 86 577 women identifies five novel loci and highlights maternal genetic effects that are independent of fetal genetics. *Human Molecular Genetics* 27(4)**,** 742-756. doi: 10.1093/hmg/ddx429.
2. Brion, M.-J.A., Shakhbazov, K., and Visscher, P.M. (2013). Calculating statistical power in Mendelian randomization studies. *International Journal of Epidemiology* 42(5)**,** 1497-1501. doi: 10.1093/ije/dyt179.
3. Horikoshi, M., Beaumont, R.N., Day, F.R., Warrington, N.M., Kooijman, M.N., Fernandez-Tajes, J., et al. (2016). Genome-wide associations for birth weight and correlations with adult disease. *Nature* 538(7624)**,** 248-252. doi: 10.1038/nature19806.
4. Horikoshi, M., Yaghootkar, H., Mook-Kanamori, D.O., Sovio, U., Taal, H.R., Hennig, B.J., et al. (2013). New loci associated with birth weight identify genetic links between intrauterine growth and adult height and metabolism. *Nature Genetics* 45(1)**,** 76-82. doi: 10.1038/ng.2477.
5. Nikpay, M., Goel, A., Won, H.-H., Hall, L.M., Willenborg, C., Kanoni, S., et al. (2015). A comprehensive 1000 Genomes-based genome-wide association meta-analysis of coronary artery disease. *Nature Genetics* 47(10)**,** 1121-1130. doi: 10.1038/ng.3396.
6. Scott, R.A., Scott, L.J., Mägi, R., Marullo, L., Gaulton, K.J., Kaakinen, M., et al. (2017). An Expanded Genome-Wide Association Study of Type 2 Diabetes in Europeans. *Diabetes* 66(11)**,** 2888-2902. doi: 10.2337/db16-1253.
