# Supplementary material for: Causal Association Between Birth Weight and Adult Diseases: Evidence From a Mendelian Randomization Analysis
Source: Front Genet. 2019 Jul 10;10:618. doi: 10.3389/fgene.2019.00618 (PMC6635582; doi:10.3389/fgene.2019.00618)
Supplement: Supplementary file 1 [file DataSheet_1.zip › 2019-03-26 Supplementary Text and Results.pdf]

Supplementary Text and Results

Text S1. Description of GWASs for 21 diseases

We provide a brief description on the genome-wide association studies (GWASs) for 21 diseases used in the present study. For each of these diseases, we obtained either GWAS summary statistics or individual-level data from individuals with European (EUR) ancestry.

Age-related macular degeneration

The advanced age-related macular degeneration (AMD) study (Fritsche et al., 2016) is the largest GWAS of AMD to date. This meta-analysis study combines 16,144 cases and 17,832 controls across 26 studies and contains 12,023,830 genotyped and imputed SNPs. We obtained summary statistics for all SNPs (single nucleotide polymorphisms, SNPs) from this study on http://csg.sph.umich.edu/abecasis/public/amd2015/. The summary statistics include the number of cases and controls analyzed, marginal p value after genomic control correction, and the direction of the effect size for every SNP. Because the estimated effect sizes and standard errors are unavailable from the website, we computed these quantities using sample sizes, marginal p values as well as minor allele frequency from a reference panel. Specifically, for each SNP in turn, we first obtained the minor allele frequency (MAF) of the SNP based on 503 EUR individuals in the 1000 Genomes Project (The 1000 Genomes Project Consortium, 2015) and computed the standard error following [see Equation (5)] (Pickrell, 2014). We then calculated a marginal *z* value by using both the marginal p value and the direction of the estimated effect size for the given SNP. We finally obtained the effect size based the marginal *z* value and the estimated standard error [see Equation (6)].

Alzheimer's disease

The International Genomics of Alzheimer's Project (IGAP) is a large two-stage study based upon GWAS on individuals of EUR ancestry (Lambert et al., 2013). In stage 1, IGAP meta-analyzed four previously-published GWAS datasets consisting of 17,008 Alzheimer's disease cases and 37,154 controls, on 7,055,881 genotyped and imputed

SNPs. The four datasets include the EUR Alzheimer's disease Initiative (EADI), the Alzheimer Disease Genetics Consortium (ADGC), the Cohorts for Heart and Aging Research in Genomic Epidemiology consortium (CHARGE), and the Genetic and Environmental Risk in AD consortium (GERAD). In stage 2, IGAP further genotyped 11,632 SNPs and tested for their association with Alzheimer's disease in an independent data with 8,572 cases and 11,312 controls. A final meta-analysis was also performed to combine results from both stages 1 and 2. We obtained summary statistics for all SNPs from stage 1 of this meta-analysis study on http://web.pasteur-lille.fr/en/recherche/u744/igap/igap_download.php. The summary statistics include both the estimated effect sizes and standard errors.

Parkinson's disease

The GWAS of Parkinson's disease (Pankratz et al., 2012) uses a 2-stage design. In the first stage, the study applies a logistic regression model to analyze individual-level genotypic data in each of the five Parkinson's disease GWASs separately. Afterwards, the results from these five studies are pooled together through a meta-analysis. The first stage analysis includes a total of 4,238 cases and 4,239 controls. In the second stage, the study genotyped 768 SNPs in an independent replication sample with 3,738 cases and 2,111 controls. We obtained the summary statistics in terms of the effect sizes and standard errors for 2,525,705 genotyped and imputed SNPs from this study on https://grasp.nhlbi.nih.gov/FullResults.aspx.

Chronic kidney disease

The GWAS of chronic kidney disease (CKD) (Köttgen et al., 2010) performed a meta-analysis using up to 7,173 cases and 77,567 controls from 20 predominantly population-based studies to identify susceptibility loci for reduced renal function as estimated by serum creatinine (eGFRcrea), serum cystatin c (eGFRcys) and CKD. We obtained summary statistics (effect allele, MAF, marginal p values, and the effect size direction) for 2,744,601 genotyped and imputed SNPs from the study on https://grasp.nhlbi.nih.gov/FullResults.aspx. We applied the same approach as described above in the GWAS of AMD subsection to estimate the effect sizes and standard errors.

Inflammatory bowel disease, Crohn's disease and ulcerative colitis

The International Inflammatory Bowel Disease Genetics Consortium (IIBDGC) (Liu et al., 2015) is a large GWAS with a total of 86,640 EUR individuals. The study collects 12,882 cases and 21,770 controls for inflammatory bowel disease (IBD) with 11,555,662 genotyped and imputed SNPs. It also collects 6,968 cases and 20,464 controls for ulcerative colitis (UC) with 11,113,953 genotyped and imputed SNPs, and collects 5,956 cases and 14,927 controls for Crohn's disease (CD) with 11,002,659 genotyped and imputed SNPs. For each SNP, we obtained summary statistics in terms of effect sizes and standard errors for IBD, CD and UC from the study on https://www.ibdgenetics.org/.

Primary biliary cirrhosis

The GWAS of primary biliary cirrhosis (PBC) (Cordell et al., 2015) included 2,764 cases and 10,475 controls. We obtained summary statistics in terms of effect size and 95% confidence interval for 1,134,226 genotyped and imputed SNPs from https://www.immunobase.org/downloads/protected_data/GWAS_Data/. We rescaled the length of the 95% confidence interval by 3.92 to compute the standard error for every SNP.

Celiac disease

The GWAS of celiac disease (Dubois et al., 2010) included 4,533 cases and 10,750 controls. We obtained summary statistics in terms of the effect size and 95% confidence interval for 523,456 genotyped and imputed SNPs from https://www.immunobase.org/downloads/protected_data/GWAS_Data/. We rescaled the length of the 95% confidence interval by 3.92 to compute the standard error for every SNP.

Systemic lupus erythematosus

The GWAS of systemic lupus erythematosus (SLE) (Bentham et al., 2015) included 7,219 cases and 15,991 controls. We obtained summary statistics in terms of effect size and 95% confidence interval for 7,913,883 genotyped and imputed SNPs for SLE

from https://www.immunobase.org/downloads/protected_data/GWAS_Data/. We rescaled the length of the 95% confidence interval by 3.92 to compute the standard error for every SNP.

Primary sclerosing cholangitis

The GWAS of primary sclerosing cholangitis (PSC) (Ji et al., 2017a) included 4,796 cases and 19,955 controls. We obtained summary statistics in terms of effect sizes and standard errors for 7,891,602 genotyped and imputed SNPs from http://www.ipscsg.org/.

Coronary artery disease and myocardial infarction

The CARDIoGRAMplusC4D Consortium performed a GWAS for coronary artery disease (CAD) (Nikpay et al., 2015) involved 60,801 cases and 123,504 controls; among the cases about 70% had a reported history of myocardial infarction (MI). We obtained summary statistics in terms of effect size and its standard error for 9,455,778 genotyped and imputed SNPs for CAD as well as for 9,289,492 genotyped and imputed SNPs for MI from http://www.cardiogramplusc4d.org/data-downloads/. Note that, all MI individuals were involved in the CAD GWAS as MI is a sub-phenotype of CAD and other sub-phenotypes of CAD include acute coronary syndrome, chronic stable angina, or coronary stenosis of >50% (Nikpay et al., 2015). Those subtypes of CAD are heterogeneous diseases with subtle but potentially important differences in terms to clinical manifestations and underlying genetic architecture (Nikpay et al., 2015). Consequently, exploring the causal effect of birth weight on MI and CAD, instead of CAD alone, could of potential importance in practice, especially if there is a detectable difference in the causal effects on MI vs CAD. Therefore, we have followed other MR analyses (Dixon et al., 2016; Hindy et al., 2018; Larsson Susanna et al., 2018) and analyzed both MI and CAD.

Type 2 diabetes

The DIAGRAM (DIAbetes Genetics Replication And Meta-analysis) consortium of type 2 diabetes (T2D) (Scott et al., 2017) included 26,676 T2D cases and 132,532 controls. We obtained summary statistics in terms of effect size and its standard error

for 12,056,347 genotyped and imputed SNPs for T2D as well as for 10,994,980 genotyped and imputed SNPs for T2D adjusted for body mass index (T2D_BMI) from http://diagram-consortium.org/downloads.html.

Type I diabetes, rheumatoid arthritis and hypertension

We obtained summary statistics for type I diabetes (T1D), rheumatoid arthritis (RA) and hypertension (HT) using the individual-level data from the Wellcome trust case control consortium study 1 (WTCCC1) (The Wellcome Trust Case Control Consortium, 2007). Specially, the WTCCC1 data consists of about 14,000 cases from seven common diseases and 2,938 shared controls. The cases include 1,963 individuals with T1D, 1,860 individuals with RA and 1,952 individuals with HT. We obtained quality controlled genotypes from WTCCC 1 and imputed missing genotypes using BIMBAM (Guan and Stephens, 2008). We obtained a total of 458,868 SNPs shared across all individuals. We then further imputed SNPs using the reference panel of the EUR individuals in the 1000 Genomes Project (The 1000 Genomes Project Consortium, 2015) with SHAPEIT and IMPUTE (Howie et al., 2009; Delaneau et al., 2012; Delaneau et al., 2013a; Delaneau et al., 2013b). We filtered out SNPs that have an HWE p value $< 10^{-4}$, a genotype call rate < 95%, or an MAF < 0.01 to obtain a total of 2,793,818 genotyped and imputed SNPs (Zeng and Zhou, 2017). For each SNP, we generated the effect size and its standard error by using an additive logistic regression model.

Ischaemic stroke

We obtained the summary statistics for ischaemic stroke (IS) from the METASTROKE Collaboration at http://cerebrovascularportal.org. The latest GWAS of ischaemic stroke included a total of 446,696 European individuals (40,585 cases and 406,111 controls) on 8,255,860 genotyped and imputed SNPs (Malik et al., 2018). The summary statistics in terms of effect size and its standard error were extracted.

Ankylosing spondylitis and multiple sclerosis

We obtained summary statistics for ankylosing spondylitis (AS) and multiple sclerosis (MS) using the individual-level data of from the Wellcome trust case control

consortium study 2 (WTCCC2) (Burton et al., 2007). We obtained quality controlled genotypes from WTCCC2, including 485,982 SNPs on 1,788 cases and 4,800 controls for AS, and 460,364 SNPs on 10,299 cases and 5,175 controls for MS. We first phased the genotypes using SHAPEIT (Delaneau et al., 2013b) and then imputed SNPs using the Haplotype Reference Consortium (HRC version r1.1) panel (McCarthy et al., 2016) on the Michigan Imputation Server (https://imputationserver.sph.umich.edu/index.html) using Minimac3 (Das et al., 2016). After filtering (HWE p value < 10^{-4}, genotype call rate < 95%, MAF < 0.01 or imputation score < 0.30), we obtained 7,566,658 genotyped and imputed SNPs for AS, and 3,863,547 genotyped and imputed SNPs for MS. For each SNP in turn, we estimated the effect size and its standard error by using an additive logistic regression model.

57 adult diseases from UK Biobank

Besides these diseases above, we also considered another set of 57 adult diseases from the UK Biobank data set (Table S7), which includes up to ~310,000 unrelated individuals of British ancestry and ~11.5 million genotyped and imputed SNPs (Sudlow et al., 2015). We obtained the summary results for these diseases from http://www.nealelab.is/uk-Biobank/ and kept only high confidence SNPs. The definition of high confidence SNPs and more detailed descriptions about the data process can be further found at http://www.nealelab.is/uk-Biobank/. Note that, despite the large number of controls, the UK Biobank data set contains extremely low number of cases for most diseases (e.g. only 119 cases of Alzheimer's disease), which can result in low statistical power.

Text S2. Description of GWASs for 38 complex traits

Here, we provide a brief description for GWASs of the 38 traits that may mediate the causal effect of birth weight onto the diseases of interest. Like that for the 21 diseases, we obtained either GWAS summary statistics or individual-level data for these 38 traits using only individuals of European ancestry.

Educational attainment

The GWAS of educational attainment (Rietveld et al., 2013), measured by years of educational attainment (EduYears, a continuous variable) and college completion (College, a binary variable), was performed by the Social Science Genetic Association Consortium (SSGAC). A total of 126,559 individuals (101,069 individuals for discovery and 25,490 for replication) were included in the study. We obtained from https://www.thessgac.org/ the summary statistics in terms of effect sizes and standard errors for 2,321,510 SNPs for College and for 2,310,087 SNPs for EduYears.

Smoking behaviors

The Tobacco and Genetics Consortium performed a GWAS to identify genetic variants associated with four smoking behaviors using 74,053 individuals (The Tobacco and Genetics Consortium, 2010). From https://www.med.unc.edu/pgc/results-and-downloads, we obtained summary statistics in terms of effect sizes and standard errors for 2,459,118 SNPs for the number of cigarettes smoked per day (CigsPerDay), 2,455,846 SNPs for smoking initiation (EverSmoke, ever versus never been a regular smoker), 2,457,545 SNPs for age of smoking initiation (AgeSmoke), and 2,456,554 SNPs for smoking cessation (FormerSmoke, former versus current smokers).

Early growth

From the Early Growth Genetics (EGG) Consortium (https://egg-consortium.org/), we obtained summary statistics in terms of effect sizes and standard errors for eight early growth traits. The data are for 2,499,691 SNPs for childhood body mass index (BMI_Child) on 35,668 individuals (Felix et al., 2016), 2,479,699 SNPs for

Growth_10_12 (height measurement at age 10 for girls and at age 12 for boys; the measurement targets the take-off phase of the growth spurt) on 13,960 individuals (Cousminer et al., 2013), 2,384,831 SNPs for Growth_PG (a measurement of the total amount of growth during the pubertal growth period) on 10,799 individuals (Cousminer et al., 2013), 2,401,289 SNPs for Growth_PT (a measurement of the total amount of growth in late adolescence, which targets the timing of peak height growth velocity) on 9,228 individuals (Cousminer et al., 2013), 2,183,228 SNPs for Tanner stage on 9,916 individuals (Cousminer et al., 2014), 2,201,971 SNPs for birth length on 28,459 individuals (van der Valk et al., 2015), 2,201,971 SNPs for childhood obesity (Obesity_Child) on 13,848 individuals (Bradfield et al., 2012), and 2,449,806 SNPs for head circumference (HC) on 10,678 individuals (Taal et al., 2012).

Lipid, glycaemic and harmonic traits

We obtained summary statistics for four lipid traits (Teslovich et al., 2010) from https://csg.sph.umich.edu/abecasis/public/lipids2010/. Specifically, we obtained the effect sizes and their standard errors of 2,692,429 SNPs for high density lipoproteins (HDL) based on an average of 97,749 individuals, 2,692,564 SNPs for low density lipoproteins (LDL) based on an average of 93,354 individuals, 2,692,413 SNPs for total cholesterol (TC) based on an average of 100,184 individuals, and 2,692,560 SNPs for triglycerides (TG) based on an average of 94,461 individuals.

We obtained summary statistics in terms of effect sizes and standard errors for six glycaemic traits from the Meta-Analyses of Glucose and Insulin-related traits Consortium (MAGIC) (https://www.magicinvestigators.org/). The data included 2,456,945 SNPs for indices of β-cell function (HOMA-B), 2,261,929 SNPs for insulin resistance (HOMA-IR), 2,470,476 SNPs for fasting glucose (FG), and 2,461,105 SNPs for fasting insulin (FI) based on up to 46,186 individuals (Dupuis et al., 2010); 2,401,708 SNPs for 2-hours glucose levels after an oral glucose (2hrGlucose) based on up to 15,234 individuals (Saxena et al., 2010); 2,423,397 SNPs for the modified stumvoll insulin sensitivity index (ISI) based on 16,753 individuals (Walford et al., 2016).

From https://grasp.nhlbi.nih.gov/FullResults.aspx, we obtained three sets of summary statistics in terms of effect sizes and standard errors for 2,474,009 SNPs on 32,161

individuals for leptin (Kilpeläinen et al., 2016), 2,699,689 SNPs on 45,891 individuals for adiponectin levels (Dastani et al., 2012), 2,450,547 SNPs on 110,347 individuals for serum urate (Kottgen et al., 2013).

Anthropometric traits

We obtained summary statistics for seven anthropometric traits from the Genetic Investigation of ANthropometric Traits (GIANT) consortium (https://portals.broadinstitute.org/collaboration/giant/index.php/GIANT_consortium_data_files). Specifically, we obtained the effect sizes and standard errors from 2,560,780 SNPs for waist-to-hip ratio (WHR), 2,565,406 SNPs for waist circumference (WC), and 2,559,737 SNPs for hip circumference (HIP), all based on up to 224,459 individuals (Shungin et al., 2015); 2,554,637 SNPs for body mass index (BMI) based on up to 339,224 individuals (Locke et al., 2015); 2,550,858 SNPs for height based on up to 253,288 individuals (Wood et al., 2014); 2,435,044 SNPs for overweight and 2,250,778 SNPs for obesity based on up to 263,407 individuals (Berndt et al., 2013).

From https://grasp.nhlbi.nih.gov/FullResults.aspx, we obtained summary statistics in terms of effect sizes and standard errors for 3,228,664 SNPs for body fat percentage based on up to 100,716 individuals (Lu et al., 2016).

In addition, we obtained summary statistics in terms of effect sizes and standard errors for weight using individual-level data of the Framingham heart study (FHS) (Splansky et al., 2007). The FHS data contains genotype data on 6,950 individuals and 394,174 SNPs. We first filtered out SNPs that have an HWE p-value $< 10^{-4}$, a genotype call rate < 95% or an MAF < 0.01 to obtain a final set of 387,741 SNPs. For these SNPs, we imputed missing genotypes with the estimated mean genotype of that SNP. Afterwards, we imputed SNPs using the Haplotype Reference Consortium (HRC version r1.1) panel (McCarthy et al., 2016) on the Michigan Imputation Server (https://imputationserver.sph.umich.edu/index.html) using Minimac3 (Das et al., 2016). After filtering (HWE p value $< 10^{-4}$, genotype call rate < 95%, MAF < 0.01 or imputation score < 0.30), we obtained 7,431,744 genotyped and imputed SNPs. We used unimputed genotypes to compute the genetic relatedness matrix following (Zeng and Zhou, 2017). Afterwards, for each SNP in turn, we applied an additive linear

mixed model implemented in the GEMMA software (version 0.94) (Zhou and Stephens, 2012) with gender and age as covariates to obtain effect size estimate and its standard error for all SNPs.

Blood pressures

We obtained summary statistics for systolic blood pressure (SBP) and diastolic blood pressure (DBP) using the individual-level data from the Atherosclerosis Risk in Communities (ARIC) Cohort (Richey Sharrett, 1992) which includes 8,749 individuals. We then imputed SNPs using the reference panel of the EUR individuals in the 1000 Genomes Project (The 1000 Genomes Project Consortium, 2015) with SHAPEIT and IMPUTE (Howie et al., 2009; Delaneau et al., 2012; Delaneau et al., 2013a; Delaneau et al., 2013b). We filtered out SNPs that have an HWE p value $< 10^{-4}$, a genotype call rate < 95% or an MAF < 0.01 to obtain a total of 1,955,110 genotyped and imputed SNPs. For each SNP, we generated the effect size and its standard error by using an additive linear regression model after adjusting for sex and age.

Text S3. Detailed methods for Mendelian randomisation analysis

Assumptions to ensure valid Mendelian randomisation analysis

Mendelian randomisation (MR) is an important statistical method to investigate the causal relationship between an exposure variable (i.e. birth weight) and an outcome variable (i.e. adult disease) in observational studies. MR selects genetic variants as instrumental variables and applies them to produce an unbiased estimate of the causal effect from the exposure variable onto the outcome variable. An important step of MR is to carefully select genetic variants to serve as valid instruments. Each of these selected genetic variants has to satisfy three conditions (Lawlor et al., 2008a; Sheehan et al., 2008) (FIGURE 1):

(i) Strongly associated with exposure

The first condition states that, for a valid MR inference, SNPs need to be (strongly) associated with the exposure variable of interest (i.e. birth weight). This condition, however, does not require these SNP instruments to be directly associated with the exposure. Instead, these instruments can be associated with exposure indirectly through some intermediator variables. In the case of our study, there are many intermediate variables (e.g. some in uterus exposures) between the genetic instruments and birth weight. The existence of such intermediate variables does not in themselves affect the validity of MR inference, as long as these intermediate variables do not serve as confounding factors (more details in the next paragraph). Indeed, almost all complex traits examined in all existing condition studies are likely affected by instruments through intermediate variables. For example, in the case of the classical condition study on body mass index (BMI) on cardiovascular disease (CAD) (Lyall et al., 2017), the instruments for BMI are likely associated with BMI through leptin pathway or other biological and metabolic pathways. Therefore, the presence of intermediator variables themselves does not affect the validity of condition **i**.

(ii) Not associated with any other confounders that may be associated with both exposure and outcome

The second condition states that, for a valid MR inference, the selected instruments cannot be associated with any other confounders that may be associated with both exposure and outcome. In the case of birth weight, if the instruments are associated with, for example, some exposures in utero, then these in utero exposures cannot be associated with the outcome disease in order to ensure a valid causal inference of birth weight on the disease. In the case of the classical BMI on CAD example, if the instruments for BMI are associated leptin pathway or other biological pathways, then these pathways cannot be directly or indirectly associated with CAD in order to ensure a valid causal inference of BMI on CAD. Potential confounding factors in our study may include various in utero environmental exposures (e.g. maternal hormone levels), maternal lifestyle (e.g. diet physical activity, smoking, alcohol drinking, folic acids intake), familial socioeconomic position (e.g. familial income and parental education and occupational class), current BMI, or weight (Lawlor et al., 2017; Zanetti et al., 2018).

(iii) Only influences the disease by the path of exposure

The third condition states that the instruments have only an indirect impact on adult diseases via birth weight and cannot influence adult diseases by other known/unknown biological pathways. That is, the instruments do not display horizontal pleiotropy and does not exhibit their effects through other mediators.

The above three conditions are also commonly referred to as the three assumptions of MR. As has been well recognized in the field, the validity of MR analysis depends on the validity of the above three assumptions. Note that, while the first condition can be directly tested based on the observed data, the second two conditions are difficult to validate due to variables that may not be recorded in the study. In fact, it is impossible to consider all confounding factors for any MR study (e.g. for the classic BMI on CAD example, there are so many metabolic and biological pathways that could confound both, and it is impossible to measure them all). The infeasibility to validate the second assumption is a known drawback of MR analysis and is well appreciated in both MR and causal statistics inference fields. Importantly, for most MR studies, violation of the second and third assumption is usually due to the pleiotropic effects of

SNPs on various confounding factors that influence both the exposure and outcome or mediators that mediate other pathways from the exposure to the outcome. For example, it is possible in our study that fetal SNP instruments affect adult BMI, which is correlated with both fetal body weight and disease status. In our case of birth weight (or the study of other birth/infant measurements), however, the violation of the second assumption can also be due to the induced effects of SNPs on these confounding factors, which are induced by the genetic effects of maternal SNPs on the confounding factors and the correlation between fetal SNPs and maternal SNPs. As a concrete example, due to the correlation between fetal and maternal SNPs, fetal SNP instruments might be associated with maternal BMI which further affects various in utero environmental exposures to be associated with adult diseases.

While the second and third MR assumptions are known to be impossible to be fully validated, we have followed previous MR studies and have previously performed two sets of sensitivity analyses to guard against these two forms of possible violations of this assumption (details in the Results section). Specifically, (**i**) in the first set of sensitivity analyses, we excluded all instruments with potential horizontal pleiotropic effects by removing instruments that are strongly associated with the disease with a marginal p value below the 0.05 level after Bonferroni correction. Intuitively, if an instrument is associated with some in utero exposures that are also associated with adult diseases, then this instrument would be associated with adult disease. Therefore, excluding pleiotropic SNPs that are associated with adult diseases would likely exclude such confounding due to pleiotropy. (**ii**) In the second set of sensitivity analyses (including the simulations in Text S4), we also excluded SNPs that have potential maternal effects on birth weight (e.g. FIGURE S12-S13). Intuitively, if an instrument is associated with birth weight through maternal effects, and such maternal effects are also associated with adult diseases, then the instrument would also be associated with birth weight with detectable maternal effects. Therefore, excluding maternal SNPs that are associated with adult diseases would likely exclude confounding due to maternal effects.

Finally, we follow the spirit of other existing MR studies and emphasize that it is important to make progress and report findings as long as the statistical modeling and analysis assumptions are clearly stated, even though the second and third assumptions

for any MR study can never be fully validated (Burgess et al., 2017a).

Birth weight as a modifiable exposure

We describe birth weight as a "modifiable exposure" in the present study based on definition. Specifically, we use the term "exposure" for birth weight because we treat the birth weight variable as an exposure in our MR analysis — a regression based statistical modeling and analysis. As is well-defined in statistics literature, an exposure variable is the one that is used to predict or explain an outcome variable in a regression setting. In the epidemiology literature, the term "exposure" is also broadly applied to any factor that may be associated with an outcome of interest (e.g. (Lee and Pickard, 2013)). Therefore, birth weight is considered as an exposure in our study. We also use the term "modifiable" because birth weight can be modified by many environmental exposures or behaviors — for example, smoking and intake of folic acid, as have been well documented in previous epidemiology studies (Chomitz et al., 1995; Rogowski, 1998; Conde-Agudelo et al., 2006; Collier and Hogue, 2007). Nevertheless, birth weight is different from the classical non-modifiable factors such as age or ethnic background or sex, which cannot be considered as an outcome induced by other environmental factors. In addition, birth weight is not a terminal outcome that does not influence any other disease, any other physiological measurement, or any other complex trait — in fact, one would hardly imagine that any complex trait could be considered as a terminal outcome given the complexity of biology. Importantly, in the main text our results show that birth weight has negative effects on various diseases, but not vice versa (as shown in the reverse causation sensitivity analysis), thus demonstrating that birth weight is not a terminal outcome but a modifiable exposure.

It is important to note that birth weight can be considered as an outcome variable in other settings. Indeed, the wording choice of "modifiable exposure" and "outcome" is relative and depends on the particular question one examines. The question of interest determines whether a variable is placed on the left-hand side of the regression equation (and thus is considered as an outcome) or on the right-hand side of the equation (and thus is considered as a modifiable exposure). This important point on the relative meaning of exposure vs outcome is generally applied to any complex

traits examined in all previous MR studies — or, in fact, more generally, for all regression based statistical analyses. Let's look at a classical MR analysis that examines the causal effect of BMI on CAD (Holmes et al., 2014a; Lyall et al., 2017; Mendelson et al., 2017). In this analysis, BMI is considered as an exposure because it is placed on the right-hand side of the equation; while BMI is considered as modifiable because there are many environmental factors or individual behaviors can modify one's BMI values. Importantly, precisely because BMI is modifiable, BMI can also be considered as an outcome in other settings where one is interested to examine factors that are associated with BMI. Additionally, BMI is also "an end-point marker of multiple (environmental, behavioral and metabolic) exposures, and a complex trait in itself, influenced directly by genetic factors in addition to the environment". Besides BMI, the same reason above applies to any other complex traits (e.g. systolic blood pressure, low density lipoprotein) listed in those proof-of-concept MR studies.

Selecting instrumental variables for birth weight

To select genetic variants (i.e. SNPs) that can serve as valid instrumental variables for birth weight, we first obtained summary statistics of GWAS from the public portal (http://egg-consortium.org/birth-weight-2016.html) for the EGG consortium study (Horikoshi et al., 2016). The EGG consortium study is the largest association study performed to date on birth weight (a continuous trait) and contains association results for 16,245,523 genotyped and imputed SNPs based on up to 153,781 individuals collected from 35 studies. In the EGG consortium study, an additive linear regression model was applied to examine one SNP at a time to detect the SNP associations with birth weight while properly adjusting for gestational week and study-specific ancestry whenever these variables were available (Horikoshi et al., 2016). For the present study, we obtained summary statistics in terms of effect allele, effect allele frequency, marginal effect size, standard error, p value and sample size for a subset of SNPs (see below) in the EGG consortium study that contains up to 143,677 individuals with EUR ancestry from 35 sub-studies (Table S1).

To minimize the bias that may arise in the MR analysis, we first carefully identify a small set of index SNPs that are independent with each other and that are associated with birth weight at the genome-wide significance level (p < 5.00E-8). To do so, we

followed (Noyce et al., 2017) and applied the plink software (version v1.90b3.38) (Purcell et al., 2007). In the plink clumping procedure, we set the significance threshold for the index SNPs and the secondary significance threshold for SNPs to be both 5.00E-8, and we set the linkage disequilibrium (LD) threshold and the physical distance threshold to be 0.001 and 1 Mb, respectively, based on a reference panel constructed by 503 individuals with EUR ancestry from the 1000 Genomes Project (The 1000 Genomes Project Consortium, 2015). We selected SNPs that pass the genome-wide significance level to avoid potential estimation bias due to weak instruments. We also selected SNPs to be independent from each other to avoid potential estimation bias due to SNP pleiotropy — where SNPs exhibit effects on adult diseases through pathways other than birth weight. Therefore, the procedure employed above ensures the validity of MR analysis. While the procedure itself is statistical in nature, we validated that the selected 47 SNPs all reside within the previously identified birth weight associated loci and are thus potentially causal candidate SNPs for birth weight (Table 1 and Table S1) (Horikoshi et al., 2016).

Among the 47 independent index SNPs to serve as instruments, 25 (53.1%) variants have negative effects on birth weight and 22 (46.9%) have positive effects, and their effect size estimates are mostly in the range of -0.06 and 0.06 (with the only exception of rs138715366 whose effect size is estimated to be -0.24; Table 1 and FIGURE S1). We computed the proportion of variance of birth weight explained (PVE) by each of these genetic variants, and the estimated PVE values range from 2.04E-4 (rs28415607) to 1.25E-3 (rs900399) (Table 1). With summary statistics, we further estimated that the 47 SNPs in total explain 1.70% of phenotypic variance of birth weight, which is close to the total variance estimate of 2.00% by all the significant SNPs identified in the original paper by using individual-level genotypes (Horikoshi et al., 2016).

We used these SNPs in our main MR analysis to estimate the causal effect of birth weight on 21 adult diseases. These diseases include advanced age-related macular degeneration (AMD) (Fritsche et al., 2016), Alzheimer's disease (Lambert et al., 2013), Parkinson's disease (Pankratz et al., 2012), chronic kidney disease (CKD) (Köttgen et al., 2010), celiac disease (Dubois et al., 2010), inflammatory bowel disease (IBD) (Liu et al., 2015), Crohn's disease (CD) (Liu et al., 2015), ulcerative colitis (UC) (Liu et al., 2015), primary biliary cirrhosis (PBC) (Cordell et al., 2015), primary sclerosing

cholangitis (PSC) (Ji et al., 2017b), systemic lupus erythematosus (SLE) (Bentham et al., 2015) coronary artery disease (CAD) (Nikpay et al., 2015), myocardial infarction (MI) (Nikpay et al., 2015), type 2 diabetes (T2D) (Scott et al., 2017) rheumatoid arthritis (RA) (The Wellcome Trust Case Control Consortium, 2007), type 1 diabetes (T1D) (The Wellcome Trust Case Control Consortium, 2007), hypertension (The Wellcome Trust Case Control Consortium, 2007), ankylosing spondylitis (AS) (Burton et al., 2007), ischaemic stroke (IS) (Malik et al., 2018) and multiple sclerosis (MS) (Burton et al., 2007).

To examine the causal effect of birth weight on each of the 21 diseases, we first obtained summary statistics from publicly available GWASs for these diseases (details in Text S2; all are case-control studies). For each disease in turn, we extracted summary statistics for the 47 index SNPs that serve as instrumental variables for birth weight. The summary statistics are in the form of effect allele, marginal effect size in terms of log odds ratio (OR), and standard error for the estimated effect size. The effect allele of SNPs is matched between birth weight and each of these diseases. For some diseases, the summary statistics are not available for all 47 index SNPs, and in these cases, we replaced the missing SNPs with proxy variants that are in high LD with the missing SNPs. Specifically, for each missing SNP, we extracted SNPs that are located within either 1 Mb upstream or 1 Mb downstream of the missing SNP. We then identified among them a proxy SNP that is most highly correlated with the missing one based on LD information obtained from 503 individuals with European ancestry in the 1000 Genomes Project (The 1000 Genomes Project Consortium, 2015). For the identified proxy SNPs, we only retained those that are highly correlated with the corresponding index SNPs (with correlation coefficient $r > 0.9$) following (Noyce et al., 2017). However, our results are not sensitive to the correlation coefficient threshold cutoff we use, and threshold cutoffs of 0.5/0.6/0.7/0.8 all lead to similar results (FIGURE S3).

To exclude instruments with potential horizontal pleiotropic effects on the disease, we followed (Østergaard et al., 2015) and removed instruments that are strongly associated with the disease with a marginal p value below the 0.05 level after Bonferroni correction (i.e. $p < 0.05/k$, where k is the number of the instrumental variables available for the given disease). Note that excluding instruments that are

strongly associated with the disease is a conservative strategy to ensure the validity of the MR analysis (i.e. condition iii in FIGURE 1) — by focusing on only instruments that do not have horizontal pleiotropic effects, we can ensure that these instruments only influence the disease by the path of exposure (Nelson et al., 2015; Østergaard et al., 2015; Censin et al., 2017). The final set of SNPs used as instrumental variables differs across different diseases and ranges from 23 (for Parkinson's disease) to 47 (for Crohn's Disease).

Here, we note that the smaller estimated causal effect sizes on late onset cardiometabolic disorders when using "without pleiotropic instruments" may appear to be concerning at the first glance (Table S6). However, the smaller estimates after we remove "pleiotropic instruments" [for late onset cardiometabolic disorders, for many other disorders examined in the present paper, as well as for most traits/diseases examined in all previous MR papers (e.g. (Østergaard et al., 2015))] are known mathematical consequences that are guaranteed to be observed for most MR studies (we say "most" here to be precise because the phenomena does depend on the relative strength of horizontal vs vertical pleiotropic effects in these removed instruments). Such phenomena itself does not imply whether there is horizontal pleiotropy contamination or whether we simply over-adjust for vertical pleiotropic effects and underestimate the true causal effect after removing them. Specifically, when "pleiotropic instruments" are indeed instruments that have horizontal pleiotropic effects, then the smaller estimates after removing these instruments would suggest that the earlier estimates with these instruments were upward biased. However, when the "pleiotropic instruments" are instruments that have vertical pleiotropic effects, then the smaller estimates after removing these instruments are instead downward biased. Because we cannot tell whether the removed instruments are truly "horizontal pleiotropic instruments" or "vertical pleiotropic instruments" (Bowden et al., 2015; Hemani et al., 2018; Verbanck et al., 2018), we cannot draw any conclusion simply based on the smaller estimates observed after removing the pleiotropic instruments. Therefore, in the paper we have followed previous studies (Østergaard et al., 2015) and report both the small estimates (which are potentially downward biased estimates if the instruments we removed have vertical pleiotropic effects) and the large estimates (which are potentially upward biased estimates if the instruments we removed have horizontal pleiotropic effects), and treat the small effects as

conservative estimates.

While our main MR analyses were performed using 47 SNPs as instrumental variables, to examine the robustness of the results, we also performed an alternative MR analysis using a slightly different set of 48 SNPs as instrumental variables. These 48 SNPs are presented in the original GWAS of birth weight (Horikoshi et al., 2016) and are also independent index SNPs that show strong association with birth weight ($p < 5.00E-8$) (Table S2). The two sets of SNPs (the set of 47 SNPs and the other set of 48 SNPs) share 24 SNPs in common. These 24 SNPs explained 8.27‰ phenotypic variation of birth weight, about 48.5% of phenotypic variance among the set of 47 SNPs and 46.1% among the set of 48 SNPs, suggesting that the shared SNPs account for about half of the phenotypic variance as one might expect. With these 48 SNPs, we follow the same procedure described above to perform MR analyses. Additionally, 7 independently associated SNPs yielded from Horikoshi et al (2013) (Horikoshi et al., 2013) (Table S4) are also employed to be instruments for further validation.

Impute missing SNPs with summary statistics

In addition, besides replacing the missing index SNPs with proxy SNPs, we also considered imputing the summary statistics for the missing index SNPs directly using an imputation procedure (Pasaniuc et al., 2014). For a missing SNP i, we first impute its z score, denoted by z_i, via the multivariate Gaussian approximation strategy. It is well known that z score proximately fellows a standard normal distribution under the null hypothesis that there is no association between SNP i and the phenotype. Assume there are m SNPs in a locus in which the missing SNP i is included and let the corresponding z scores $\mathbf{z} = (z_1, \ldots, z_{i-1}, z_i, z_{i+1}, \ldots, z_m)$. Thus, under the null, the vector of z scores for all the SNPs is approximately distributed as a multivariate normal distribution

$$\mathbf{z} \sim N(0, \boldsymbol{\Sigma}), \quad (1)$$

where $\boldsymbol{\Sigma}$ is the correlation matrix for all the m SNPs. Note that, we cannot compute $\boldsymbol{\Sigma}$ directly as the individual level genotypes for these SNPs are not available. Instead, we estimate $\boldsymbol{\Sigma}$ using the genotypes of the 503 individuals with European ancestry in the 1000 Genomes Project (The 1000 Genomes Project Consortium, 2015). In the present

paper, we define a locus being a continuous genetic region that is within 100 kb of the missing SNP. Note that, z_i is of our interest, and $\mathbf{z}_{-i}$ is the vector of the observed z scores for these typed SNPs that are nearby SNP i. Under the null we have

$$\begin{aligned} \mathbf{z}_{-i} &\sim N(0, \mathbf{\Sigma}_{-i,-i}), \\ z_i &\sim N(\mathbf{\Sigma}_{i,\cdot}\mathbf{\Sigma}_{-i,-i}^{-1}\mathbf{z}_{-i}, \mathbf{\Sigma}_{i,\cdot}\mathbf{\Sigma}_{-i,-i}^{-1}\mathbf{\Sigma}_{i,\cdot}^{T}), \end{aligned} \tag{2}$$

where $\mathbf{z}_{-i}$ denotes the vector of $\mathbf{z}$ in which the ith element is removed, $\mathbf{\Sigma}_{-i,-i}$ denotes the matrix of $\mathbf{\Sigma}$ in which the ith row and i^{th} column are removed, and $\mathbf{\Sigma}_{i,\cdot}$ denotes the ith row of $\mathbf{\Sigma}$. To be sure that $\mathbf{\Sigma}$ is computationally invertible, we compute $\mathbf{S} = \mathbf{\Sigma} + \lambda\mathbf{I}_m$, where $\mathbf{I}_m$ is the m-dimensional identify matrix and $\lambda = 0.1$ as suggested (Pasaniuc et al., 2014). Finally, we impute z_i for the missing SNP i as

$$z_i = \frac{\mathbf{S}_{i,\cdot}\mathbf{S}_{-i,-i}^{-1}\mathbf{z}_{-i}}{\sqrt{\mathbf{S}_{i,\cdot}\mathbf{S}_{-i,-i}^{-1}\mathbf{S}_{i,\cdot}^{T}}}. \tag{3}$$

Then, under the null the p value of z_i can be obtained by assuming that z_i fellows a standard normal distribution. To obtain the variance of the estimated effect size $\hat{\beta}_i$ for the missing SNP i, following previous work (Pickrell, 2014), we estimate it as

$$\text{Var}(\hat{\beta}_i) = \frac{1}{N \times \text{MAF}_i \times (1 - \text{MAF}_i)}, \tag{4}$$

for continuous traits, or as

$$\text{Var}(\hat{\beta}_i) = \frac{N_1 + N_2}{2 \times N_1 \times N_2 \times \text{MAF}_i \times (1 - \text{MAF}_i)}, \tag{5}$$

for binary traits (i.e. case-control studies); where N is the total sample size, N_1 and N_2 are the sample sizes for cases and controls, MAF_i is the minor allele frequency for the missing SNP i and is estimated from the 1000 Genomes Project (The 1000 Genomes Project Consortium, 2015). The effect size $\hat{\beta}_i$ is estimated as

$$\hat{\beta}_i = z_i \times \sqrt{\text{Var}(\hat{\beta}_i)}. \tag{6}$$

We also employ the minor allele of SNP i in the 1000 Genomes Project (The 1000 Genomes Project Consortium, 2015) as the risk (or effect) allele. Our results are again not sensitive to whether we replace index SNPs with proxy ones or impute the

summary statistics for the index SNPs (FIGURE S4).

Calculating the proportion of variance explained by SNPs using summary statistics

Here, we describe our approach to compute the proportion of variance explained (PVE) by the examined SNPs. To do so, we denote G_i as an N_i-vector of genotypes for SNP i and $\mathbf{x}$ as an N_i-vector of exposure variables (i.e. birth weight), where N_i is the sample size available to obtain summary statistics for SNP i. We assume that the relationship between G_i and $\mathbf{x}$ is characterized by a linear model (Balding, 2006)

$$\mathbf{x} = \mu + G_i\beta_i + \varepsilon, \varepsilon \sim N(0, \sigma^2), \quad (7)$$

where μ is the intercept, β_i is the effect size for SNP i, and ε is an N_i-vector of residual errors that each follows a normal distribution with mean 0 and variance σ^2. We can decompose the phenotypic variance as

$$\text{Var}(\mathbf{x}) = \text{Var}(G_i)\beta_i^2 + \sigma^2, \quad (8)$$

where Var denotes the sample variance. Therefore, the proportion of variance in phenotypes explained (Yang et al., 2010; Zhou et al., 2013) by SNP i can be defined as

$$\text{PVE}_i = \frac{\text{Var}(G_i)\beta_i^2}{\text{Var}(\mathbf{x})} = \frac{\text{Var}(G_i)\beta_i^2}{\text{Var}(G_i)\beta_i^2 + \sigma^2} \approx \frac{\text{Var}(G_i)\hat{\beta}_i^2}{\text{Var}(G_i)\hat{\beta}_i^2 + \hat{\sigma}^2}, \quad (9)$$

where $\hat{\beta}_i$ and $\hat{\sigma}^2$ are the estimates of β_i and σ^2, respectively. Under Hardy-Weinberg equilibrium (HWE), Var(G_i) can be estimated by 2 × MAF_i × (1-MAF_i), where MAF_i denotes the minor allele frequency of SNP i. In addition, following (Pickrell, 2014), we can estimate σ^2 using MAF_i by

$$\hat{\sigma}^2 \approx 2 \times \text{Var}(\hat{\beta}_i) \times N_i \times \text{MAF}_i \times (1 - \text{MAF}_i), \quad (10)$$

where $\text{Var}(\hat{\beta}_i)$ is the variance of $\hat{\beta}_i$. Therefore, PVE_i for SNP i can be estimated as

$$\begin{aligned}\mathrm{PVE}_i &\approx \frac{\hat{\beta}_i^2 \times \mathrm{MAF}_i \times (1-\mathrm{MAF}_i)}{\hat{\beta}_i^2 \times \mathrm{MAF}_i \times (1-\mathrm{MAF}_i) + \mathrm{Var}(\hat{\beta}_i) \times N_i \times \mathrm{MAF}_i \times (1-\mathrm{MAF}_i)}, \\ &\approx \frac{\hat{\beta}_i^2}{\hat{\beta}_i^2 + \mathrm{Var}(\hat{\beta}_i) \times N_i}. \end{aligned} \tag{11}$$

Both $\mathrm{Var}(\hat{\beta}_i)$ and N_i are available from GWAS summary data (Pasaniuc and Price, 2017). Because we selected instrumental variables to be independent from each other (correlation coefficient r < 0.001 for any pairs of SNPs), the total PVE by all instrumental variables is a simple summation of individual PVE_i; that is PVE = $\sum_{i=1}^{k} \mathrm{PVE}_i$ with k denoting the number of instrumental variables.

Evaluating the strength of instrumental variables

An important condition for a valid MR analysis is that the selected instruments should have relatively strong effects on exposures (i.e. condition **i** as described in the first subsection) (Lawlor et al., 2008a). Strong instruments are important to guard the asymptotic properties of instrumental methods and ensure unbiased causal effect estimation (Bound et al., 1995; Burgess and Thompson, 2011; Burgess and Thompson, 2012; Keele and Morgan, 2016). Note that our study is a two-sample MR where the discovery sample and outcome sample are not the same. In a one-sample MR, the discovery sample is also the outcome sample and one can use this sample to evaluate the effect size strength of an instrument on the exposure variable. In a two-sample MR, the discovery GWAS sample only contains the summary data of the instruments and the exposure variable of interest on one group of individuals, while the outcome GWAS sample only contains the summary data of the instruments and the outcome variable on another group of (different or partially overlapped) individuals. Under this situation, examining the issue of weak instruments is not easy and needs additional model assumptions. Therefore, to evaluate the instrument effect on the exposure variable, we follow other studies (e.g. (Noyce et al., 2017)) to use only the discovery sample (e.g. in the present paper, the discovery sample is the EGG consortium study for birth weight up to 153,781 individuals). In this case, the underlying assumption of a two-sample MR is that the instrument's effect on the exposure is similar in the discovery and outcome samples. This assumption of similar instrument effect in the

two samples can certainly be violated in certain settings, but often works well in settings where the discovery and outcome samples are of the same ethnicity.

Specifically, to examine whether the selected instrumental variables have significant and strong association effects with birth weight, we follow (Noyce et al., 2017) and compute *F* statistic (also known as the Cragg-Donald statistic) (Cragg and Donald, 1993; Burgess et al., 2017b). The general form of the *F* statistic is

$$F_i = \frac{\mathrm{PVE}_i (N_i - 1 - k)}{(1 - \mathrm{PVE}_i) \times k}, \tag{12}$$

where k is the number of instrumental variables under examination (in the case of a single SNP, $k = 1$) and N_i is again the sample size for SNP i. We use the above form to compute an F_i statistic for each SNP in turn. In addition, we also compute an overall F statistic for all selected SNPs jointly

$$F = \frac{(\sum_{i=1}^{k} \mathrm{PVE}_i)(\bar{N} - 1 - k)}{(1 - (\sum_{i=1}^{k} \mathrm{PVE}_i)) \times k}, \tag{13}$$

where $\bar{N}$ is the average sample size across all the selected SNPs/instruments. In the present study, $\sum_{i=1}^{k} \mathrm{PVE}_i$ = 1.70%, k = 47 and $\bar{N}$ = 136,343. As a rule of thumb, an F statistic above the threshold of 10 is usually considered as an indicator for strong instrument(s) (Staiger and Stock, 1997).

Estimating the causal effects

Here, we describe details for the MR analysis, which is a special form of instrumental variable analysis. For exposure **x**, let's assume that the effect size estimate and its variance for instrumental variable i are $\hat{\beta}_i^x$ and $\mathrm{Var}(\hat{\beta}_i^x)$ (i = 1, 2, …, k), respectively (Table 1). We denote the disease of interest as **y** and denote the effect size estimate and its variance for the same instrumental variable i on **y** as $\hat{\beta}_i^y$ and $\mathrm{Var}(\hat{\beta}_i^y)$, respectively. Let the causal effect of **x** (i.e. birth weight) on **y** (i.e. the disease of interest) be θ. θ is on the original scale if **y** is a continuous outcome. If **y** is a binary outcome, then θ is on the log-scale (i.e. log of OR) and will be transformed

into OR by exponentiation. For a single instrumental variable, say SNP i, θ can be estimated as

$$\hat{\theta}_i = \frac{\hat{\beta}_i^y}{\hat{\beta}_i^x}. \tag{14}$$

Supposing that both $\hat{\beta}_i^x$ and $\hat{\beta}_i^y$ follow a normal distribution, with the delta method, we can estimate the asymptotic variance of $\hat{\theta}_i$ by

$$\mathrm{Var}(\hat{\theta}_i) = \frac{\mathrm{Var}(\hat{\beta}_i^y)}{(\hat{\beta}_i^x)^2} + \frac{(\hat{\beta}_i^y)^2\mathrm{Var}(\hat{\beta}_i^x)}{(\hat{\beta}_i^x)^4} - \frac{2\hat{\beta}_i^y\mathrm{Cov}(\hat{\beta}_i^y, \hat{\beta}_i^x)}{(\hat{\beta}_i^x)^3}. \tag{15}$$

Assuming $\mathrm{Cov}(\hat{\beta}_i^y, \hat{\beta}_i^x) = 0$, the asymptotic variance of $\hat{\theta}_i$ can be further expressed as (Thomas et al., 2007; Burgess and Thompson, 2017)

$$\mathrm{Var}(\hat{\theta}_i) \approx \frac{\mathrm{Var}(\hat{\beta}_i^y)}{(\hat{\beta}_i^x)^2} + \frac{(\hat{\beta}_i^y)^2\mathrm{Var}(\hat{\beta}_i^x)}{(\hat{\beta}_i^x)^4}. \tag{16}$$

When there are multiple instrumental variables, we combine the above estimates from all these instrumental variables together through the inverse-variance weighted (IVW) methods (Burgess et al., 2017b; Hartwig et al., 2017). Two versions of IVW methods exist: a fixed-effects version and a random-effects version (Yavorska and Burgess, 2017). The fixed-effects version produces a final estimate of the causal effect θ^{yx} by combing the point estimates from all SNPs through a weighted average (DerSimonian and Laird, 1986; Thompson and Sharp, 1999; Brockwell and Gordon, 2001)

$$\hat{\theta}_{\text{fixed}} = \frac{\sum_{i=1}^k \mathrm{Var}(\hat{\beta}_i^y)^{-1}\hat{\beta}_i^y\hat{\beta}_i^x}{\sum_{i=1}^k \mathrm{Var}(\hat{\beta}_i^y)^{-1}(\hat{\beta}_i^x)^2}. \tag{17}$$

We call the above $\hat{\theta}_{\text{fixed}}$ the inverse-variance weighted fixed-effects estimate of θ and we obtain the variance of $\hat{\theta}_{\text{fixed}}$ as

$$\mathrm{Var}(\hat{\theta}_{\text{fixed}}) = \frac{1}{\sum_{i=1}^k \mathrm{Var}(\hat{\beta}_i^y)^{-1}(\hat{\beta}_i^x)^2}. \tag{18}$$

The random-effects version of IVW views the estimation Equation (17) through an inverse-variance weighted linear regression framework and considers the following

equation (Burgess and Thompson, 2017)

$$\hat{\beta}_i^y = \hat{\beta}_i^x \theta^{yx} + e_i, e_i \sim N(0, \tau \mathrm{Var}(\hat{\beta}_i^y)). \tag{19}$$

where τ is the residual error variance. That is, the random-effects version of IVW treats $\hat{\beta}_i^y$ as the response and $\hat{\beta}_i^x$ as the exposure and relies on a heterogeneous variance regression with variance weights proportional to $\mathrm{Var}(\hat{\beta}_i^y)$. Note that the intercept is omitted in the above model to satisfy the assumption that the outcome is zero whenever the exposure is zero. With Equation (19), we estimate the regression coefficient $\hat{\theta}$ in the same form as that in Equation (17), though we refer to the resulting estimate as the inverse-variance weighted random-effects estimate $\hat{\theta}_{\mathrm{random}}$. Although the point estimate from the random-effects model is the same as that from the fixed-effects model, the estimated variance of $\hat{\theta}_{\mathrm{random}}$ is different

$$\mathrm{Var}(\hat{\theta}_{\mathrm{random}}) = \frac{\hat{\tau}}{\sum_{i=1}^{k} \mathrm{Var}(\hat{\beta}_i^y)^{-1} (\hat{\beta}_i^x)^2}, \tag{20}$$

where $\hat{\tau}$ is the estimated residual variance from Equation (19). Note that Equation (20) is equivalent to Equation (18) when $\hat{\tau}$ = 1. However, to account for the effect size estimate heterogeneity across instruments and to allow for over-dispersion in the MR model (Thompson and Sharp, 1999; Burgess and Thompson, 2017), the random-effects version of IVW restricts $\hat{\tau}$ to be equal to or greater than 1 (Yavorska and Burgess, 2017). Therefore, $\hat{\theta}_{\mathrm{random}}$ generally allows for greater estimation uncertainty than $\hat{\theta}_{\mathrm{fixed}}$. We apply both the fixed-effects version and the random-effects version (Yavorska and Burgess, 2017) in our main analysis. We also employ both the Q and I^2 statistics to measure effect size heterogeneity in the data (Thompson and Sharp, 1999).

Power calculations

To investigate whether the lack of significance for the remaining diseases other than CAD, MI and T2D is due to lack of statistical power, we performed two sets of power calculations to detect a non-zero causal effect for every disease based on (Brion et al., 2013; Freeman et al., 2013; Burgess, 2014). First, we set the causal OR of birth

weight on the disease to be either 1.10 or 0.90 per unit change of birth weight, which is close to the average estimate across all diseases (average OR = 1.12). Second, we set the causal OR of birth weight on the disease to be the same as that estimated for the given disease in the present study; thus, the causal OR changes across diseases. In both calculations, we set the total PVE by all instrumental variables to be 1.70%, set the significance level α to be 2.38E-3 (=0.05/21) for 21 diseases or 1.32E-3 (=0.05/38) for 38 other complex traits, and estimated the statistical power to detect a non-zero causal effect for each outcome in turn. In the present study, we calculated the power by using the analytic method shown in (Brion et al., 2013) that is implemented on an online software tool available at https://cnsgenomics.shinyapps.io/mRnd/.

The power calculations results show that the statistical power to detect non-zero causal effect generally increases with increasing sample size (Tables S3 and S4). For example, the estimated power is less than 1% for rheumatoid arthritis (N = 4,798) or hypertension (N = 4,890) but is 31% for coronary artery disease (N = 184,305). In addition, the statistical power is high for the three diseases identified to be causally affected by birth weight (CAD, MI, T2D), but is relatively low for the remaining 17 diseases. In particular, with the only exception of systemic lupus erythematosus (power = 26%), the power to detect a causal association between birth weight and any other disease is estimated to be below 10%. Therefore, the power calculation results suggest that the nonsignificant results for the remaining diseases may be due to a lack of statistical power, and that larger sample sizes are needed to elucidate the causal effects of birth weight on these diseases.

General sensitivity analyses for Mendelian randomisation analyses

To ensure results robustness and to guard against various modeling misspecifications in our main MR analyses, we also performed several sensitivity analyses for each of the diseases that are identified to be causally affected by birth weight.

Median-based method

First, in addition to the IVW methods, we performed the median-based method to estimate the causal effects of birth weight on diseases. Compared with the IVW methods described above, the median-based method is more robust against outlying

instruments and can offer a consistent estimate for the causal effect even when as many as 50% instrumental variables are invalid (Bowden et al., 2016a). In particular, for example, the ORs for a unit decrease in birth weight are estimated to be 1.31 (95% CI 1.11 - 1.54, p = 1.58E-3) for CAD, 1.35 (95% CI 1.12 - 1.63, p = 1.35E-3) for MI, 1.43 (95% CI 1.11 - 1.84, p = 5.22E-3) for T2D and 1.37 (95% CI 1.37 - 2.38, p = 2.71E-5) for T2D_BMI, respectively. The weighted median analysis results suggest that invalid instruments unlikely bias our main results.

Leave-one-out cross validation analysis

Second, we conducted a leave-one-out (LOO) cross validation analysis (Noyce et al., 2017) to examine potential instrument outliers. In the LOO analysis, we removed an instrumental variable at a time, and re-computed the causal effect estimate using the rest of instruments through the IVW methods. The discrepancy between the LOO estimate with one instrument left out and the original estimate obtained using all instruments was then used to quantify the influence of the instrument that is left out. The LOO analysis results are stable and demonstrate that no single instrumental variable substantially influences the estimation of the casual effects of birth weight on CAD, MI, T2D, or T2D_BMI (FIGURE S2). For example, after removing rs138715366, the ORs for a unit decrease of birth weight are estimated to be 1.34 (95% CI 1.20 - 1.49, p = 9.70E-8) for CAD, 1.30 (95% CI 1.15 - 1.47, p = 1.66E-5) for MI, 1.48 (95% CI 1.27 - 1.71, p = 2.66E-7) for T2D and 1.57 (95% CI 1.32 - 1.87, p = 3.95E-7) for T2D_BMI, almost identical to the ORs estimated using all these instrumental variables together (FIGURE 2).

MR-Egger regression

Third, we carried out MR-Egger regression to examine the assumption of directional pleiotropic effects (Bowden et al., 2016b; Burgess and Thompson, 2017). The MR-Egger regression is a modification of the inverse-variance weighted random-effects method and assumes

$$\hat{\beta}_i^y = \alpha + \hat{\beta}_i^x \theta_{\text{Egger}} + e_i, e_i \sim N(0, \tau\text{Var}(\hat{\beta}_i^y)), \tag{21}$$

where α is the intercept, θ_{Egger} is the causal effect θ^{yx}. When α is exactly zero, then

the MR-Egger estimate $\hat{\theta}_{\text{Egger}}$ of θ is equal to the random-effects IVW estimate $\hat{\theta}_{\text{random}}$. Under the InSIDE (Instrument Strength Independent of Direction Effect) assumption (Bowden et al., 2016b; Burgess and Thompson, 2017), the intercept term α in the MR-Egger regression can be interpreted as the average pleiotropic effect of the instrumental variables. If the assumption of balanced pleiotropy holds, then α will be equal to zero, and the MR-Egger estimate $\hat{\theta}_{\text{Egger}}$ of θ will be a consistent estimate of θ. In contrast, deviation of α from zero suggests either directional pleiotropy or invalid InSIDE assumption. Therefore, a significantly non-zero intercept term in the MR-Egger regression would suggest potential violation of IVW assumption and subsequently biased causal effect estimates. In the present study, we removed the SNP outlier rs138715366 before performing MR-Egger regression. For example, MR-Egger analysis shows that the ORs for a unit decrease in birth weight are estimated to be 1.56 (95% CI 0.96 - 2.54, p = 0.071) for CAD, 1.20 (95% CI 0.71 - 2.01, p = 0.491) for MI, 1.10 (95% CI 0.55 - 2.21, p = 0.796) for T2D and 1.42 (95% CI 0.68 - 3.00, p = 0.353) for T2D_BMI, respectively.

Mendelian randomization pleiotropy residual sum and outlier

The Mendelian Randomization Pleiotropy RESidual Sum and Outlier (MR-PRESSO) analysis (Verbanck et al., 2018) is a statistical method that can be employed to identify horizontal pleiotropic outliers in summary statistics based MR analysis. Like the MR-Egger regression, the MR-PRESSO outlier test also relies on the InSIDE assumption and requires at least 50% of the instruments to be valid (Bowden et al., 2016b; Burgess and Thompson, 2017). The MR-PRESSO was developed under the framework of residuals in the setting of linear regression. Specifically, use the notations defined before, the MR-PRESSO outlier test consists of four steps: (**i**) for each instrument i, estimate the causal effect using IVW by removing the ith instrument, denoted by $\hat{\theta}_{-i}$; (**ii**) compute the observed residual sum of square (RSS) for instrument i, denoted by $RSS_{\text{obs}}(i) = (\hat{\beta}_i^y - \hat{\beta}_i^x \hat{\theta}_{-i})^2$; (**iii**) compute the expected RSS for instrument i by simulations, denoted by $RSS_{\exp}^s(i) = (\hat{\beta}_{is}^{y\,\text{sim}} - \hat{\beta}_{is}^{x\,\text{sim}} \hat{\theta}_{-i})^2$, where $\hat{\beta}_{is}^{y\,\text{sim}}$ is a random variable generated from the normal distribution

$N(\hat{\beta}_i^y, \mathrm{Var}(\hat{\beta}_i^y))$ and $\hat{\beta}_{is}^{x\,\mathrm{sim}}$ is a random variable generated from the normal distribution $N(\hat{\beta}_i^x, \mathrm{Var}(\hat{\beta}_i^x))$; the simulation procedure above is repeated S simulations; (**iv**) the p value of the MR-PRESSO outlier test for instrument i is computed by $\sum_{s=1}^{S} I\left\{RSS_{\exp}^{s}(i) \geq RSS_{\mathrm{obs}}(i)\right\}$; where I is an indicator variable. In the present study we set S to 1,000. The MR-PRESSO test shows that there are no significant outliers for CAD, MI, T2D or T2D_BMI at the significance level of 0.05.

Reverse causal inference

Finally, while unlikely due to time ordering, we performed reverse causal inference to examine the possible reverse causality from diseases to birth weight. To do so, we selected associated SNPs (p < 5.00E-8) for each disease as instrumental variables following the aforementioned plink procedure and obtained 41, 26, 40 and 27 SNPs to serve as instrumental variables for CAD, MI, T2D and T2D_BMI, respectively. Afterwards, for each disease in turn, we extracted the summary statistics from birth weight GWAS for the corresponding instrumental variables (Horikoshi et al., 2016) and performed both fixed-effects and mixed-effects IVW analyses to estimate the causal effects of the given disease on birth weight. We did not use proxy SNPs or imputed summary statistics here because all index SNPs used in the reverse causal analysis have summary statistics available in the GWAS for birth weight.

Identifying complex traits that mediate the causal effect of birth weight on the three identified adult diseases

We first obtained GWAS summary statistics for 38 complex traits in adulthood (Text S2) and performed MR analysis to identify complex traits that are causally affected by birth weight. Traits causally affected by birth weight are candidate mediators that may mediate the causal effect of birth weight on the three identified adult diseases. The examined 38 complex traits include educational attainment (i.e. EduYears and College) (Rietveld et al., 2013), smoking behaviors (The Tobacco and Genetics Consortium, 2010), early growth traits (Cousminer et al., 2013), blood lipid traits (Teslovich et al., 2010), glycaemic and harmonic traits (Dupuis et al., 2010) and blood pressures (Richey Sharrett). Some of these complex traits are identified to be related to CAD

and T2D in various previous studies (Baker et al., 2007; Frayling et al., 2007; Lawlor et al., 2008b; Pare and Anand, 2010; Triglyceride Coronary Disease Genetics Consortium and Emerging Risk Factors Collaboration., 2010; The International Consortium for Blood Pressure Genome-Wide Association Studies., 2011; Voight et al., 2012; Fall et al., 2013; Afzal et al., 2014; Holmes et al., 2014b; Borges et al., 2016; Corbin et al., 2016; Lyall et al., 2016; White et al., 2016; Aikens et al., 2017; Carreras-Torres et al., 2017; Emdin et al., 2017; Lyall et al., 2017; Tillmann et al., 2017). Among them, 3 are binary traits and 35 are quantitative traits. For each of the 38 complex traits in turn, we extracted the summary statistics from the corresponding GWAS for the selected instrumental variables for birth weight. We replaced missing SNPs with proxy ones when necessary and applied the IVW methods following the same procedure as described above.

With the identified complex traits that are casually affected by birth weight, we performed a multivariable MR analysis to investigate whether any of these identified complex traits may mediate the causal effect of birth weight on the three diseases. The multivariable MR analysis is recently developed to partition the causal effects from the exposure variable to the outcome variable into two parts: a part that is mediated by the potential mediator and another part that is not mediated through the mediator (Do et al., 2013; Burgess and Thompson, 2015; Burgess et al., 2017c). For this analysis, we first regressed out the adult weight effects from the disease through a simple linear regression model and treated the resulting disease residuals as the new outcome variable. We then performed mediation analysis using birth weight and the new outcome variable as described above and obtained an estimate of the causal effect. In both these regression models we set the regression weight as the inverse variance of the variant effect size of the disease (Burgess et al., 2017c). The estimated causal effect through this approach is free of adult weight mediating effects and is referred to as the direct effect. In contrast, the originally estimated causal effect without considering the mediator is referred to the total effect. Because the indirect effect is the difference between the total effect and the direct effect (on log-OR scale), we can subsequently obtain an estimate for the indirect effect. In addition, based on asymptotic normality, we can perform a statistical test for the null hypothesis that the indirect effect is zero (Burgess et al., 2017c).

iMAP analysis to infer the directionality of causal effects

We applied a recently developed method, iMAP, to analyze birth weight and each of the three diseases that are identified in the present study to be causally affected by birth weight (i.e. CAD, MI, T2D, and T2D_BMI). iMAP is an integrative method for modeling pleiotropy and investigating causality between pairs of traits using summary statistics from GWAS (Zeng et al., 2018). Different from MR, iMAP jointly analyzes all genome-wide SNPs and has the potential to provide additional evidence supporting or against causal relationship between pairs of traits.

We provide a brief description of the iMAP method here, with further details available in the original paper (Zeng et al., 2018). iMAP models the relationship of all genome-wide SNPs together with two phenotypes. It assumes that the jth SNP effect sizes on the two traits, $\boldsymbol{\beta}_j = (\beta_{1j}, \beta_{2j})^T$ (j = 1, 2, …, m, where m the total number of SNPs), follow a four-component Gaussian mixture distribution *a priori*

$$\boldsymbol{\beta}_j \sim \pi_{11}\mathrm{MVN}_2(0, \mathbf{V}_{11}) + \pi_{10}\mathrm{MVN}_2(0, \mathbf{V}_{10}) + \pi_{01}\mathrm{MVN}_2(0, \mathbf{V}_{01}) + \pi_{00}\delta_0, \qquad (22)$$

with prior probabilities π_l (l = 11, 10, 01 and 00) that sum to one. Here, δ_0 denotes a point mass at zero, while MVN_2 denotes a two-dimensional multivariate normal distribution with $\mathbf{V}_l$ (l = 11, 10, and 01) being the corresponding variance covariance matrix. π_{11} represents the prior probability that a SNP is associated with both traits; π_{10} represents the prior probability that a SNP is associated with the first trait but not the second; π_{01} represents the prior probability that a SNP is associated with the second trait but not the first; and π_{00} represents the prior probability that a SNP is not associated with any traits. The iMAP model effectively assumes that each SNP has a probability of π_{00} to be associated with neither phenotype, a probability of π_{10} or π_{01} to be associated either one phenotype and a probability of π_{11} to be associated with both phenotypes.

iMAP aims to estimate these proportional parameters that characterize the SNP causal effects on the two phenotypes in order to better understand the relationship between the phenotypes (Zeng et al., 2018). In particular, iMAP estimates an important ratio quantity, $\pi_{11}/(\pi_{10}+\pi_{11})$ (or $\pi_{11}/(\pi_{01}+\pi_{11})$), which represents the proportion of SNPs associated with one trait that are also associated with the other. This ratio quantity has

been used to evaluate the causality of one trait on the other (Pickrell et al., 2016). Specifically, a large $\pi_{11}/(\pi_{10}+\pi_{11})$ and a small $\pi_{11}/(\pi_{01}+\pi_{11})$ suggest that a large fraction of SNPs associated with the first trait is also associated with the second trait, but not vice versa, indicating that the first trait may causally affect the second trait. A small $\pi_{11}/(\pi_{10}+\pi_{11})$ and a large $\pi_{11}/(\pi_{01}+\pi_{11})$ indicate that the second trait may causally affect the first trait. On the other hand, a large $\pi_{11}/(\pi_{10}+\pi_{11})$ and a large $\pi_{11}/(\pi_{01}+\pi_{11})$ indicate that both traits may share common biological pathways. Therefore, estimating $\pi_{11}/(\pi_{10}+\pi_{11})$ and $\pi_{11}/(\pi_{01}+\pi_{11})$ can help provide additional evidence with regard to the causal relationship between the two analyzed traits.

The technical benefits of iMAP is that it requires only GWAS summary statistics, models all SNPs jointly by a composite likelihood approach, relies on a computationally efficient expectation-maximization (EM) algorithm for scalable genome-wide analysis, and produces accurate estimates for the ratio quantities (Zeng et al., 2018). We applied iMAP to analyze birth weight and each of the three identified diseases in turn to estimate the six ratio quantities (two for each pair of traits) to better characterize the causal relationship among traits.

Text S4. Special sensitivity analyses, bias analyses and simulations for examining the influence of maternal genetic effects on the causal estimations

When estimating the causal effects of birth weight on adult diseases using MR, it is important to recognize that, as a surrogate measurement of intrauterine exposure, birth weight can be influenced by many other factors besides offspring's genetic variants. For example, maternal life styles during pregnancy (e.g. alcohol drink, smoking and nutrition intake), intrauterine environment (maternal hormone levels), and as well as mother's genetic variants (FIGURE 1) (Lawlor et al., 2017; Zanetti et al., 2018) can all influence birth weight. Therefore, examining the potential influence of parental genetic effects on postnatal offspring outcomes is essential when performing MR analyses using offspring's instrumental variables. Developing approaches that can be applied to clarify the separately maternal and offspring impacts of genetic effects on adult outcomes is an active recent area and has attracted significant research attentions (Eaves et al., 2014; Lawlor et al., 2017; Warrington et al., 2018). Therefore, in addition to the sensitivity analyses described in the previous section, we devote this section to conduct additional sensitivity analyses, bias analyses and simulations that are specific for examining the influence of maternal genetic effects on our main MR results.

First, we note that maternal genetic variants can affect offspring birth weight through maternal effects by influencing, for example, maternal behavior during pregnancy or intrauterine environment. Therefore, the instrumental variables we selected for birth weight may contain SNPs that affect birth weight through maternal effects. These maternal effect SNPs can affect adult diseases through maternal traits other than birth weight, and thus the use of such SNPs in the analysis may violate the second assumption of MR. To control for potential maternal effects induced bias, we performed a sensitivity analysis by excluding SNPs that have potential maternal effects. To do so, we obtained summary statistics from a recently published GWAS of maternal SNP effects on offspring birth weight (Beaumont et al., 2018). This GWAS collected 86,577 women and 8,741,106 genotyped and imputed SNPs in the final available summary association result. The original study identified a total of 85 maternal SNPs inside 10 genomic loci that are associated with offspring's birth weight

at the genome-wide significant level of 5.00E-8 (Table S5) (Beaumont et al., 2018). Considering the relatively small sample size of the maternal GWAS compared to that in the offspring GWAS (86,577 vs. 143,702), we decided to employ a less stringent significant level of 1.00E-5 (i.e. the suggestive significant threshold) to exclude more SNPs associated with birth weight through maternal effects. This criterion yields a total of 700 candidate SNPs that have potential maternal effects. We cross-examined our 47 instruments with these 700 maternal SNPs and excluded all instruments that reside within 1Mb of any of the birth weight associated maternal genomic loci. This excludes 10 instruments, leading to a final set of 37 instruments that unlikely contain maternal effects. Using the remaining 37 instruments, we performed the IVW analyses to examine the robustness of our results. Because excluding SNPs based on the relaxed significant level of 1.00E-5 is a rather conservative approach, we expect the results based on the remaining 37 instruments will unlikely be influenced by maternal effects.

Second, we directly controlled for maternal effects in the analysis of birth weight on T2D using a genetic-score based approach. Specially, we first obtained two sets of birth weight instruments: one set contains instruments for offspring's effect on birth weight (i.e. 47 index SNPs in Table 1) and another set contains for instruments for mother's effect on birth weight (i.e. 10 index SNPs in Table S5). We constructed two genetic scores using these instruments in the Genetic Epidemiology Research on Aging (GERA) cohort (Banda et al., 2015): one genetic score is obtained using the offspring's instruments and represents offspring effect, while another genetic score is obtained using mother's instruments and represents maternal effect. Note that there are 54,315 controls and 7,638 T2D cases in the GERA cohort. Afterwards, we examined the relationship between offspring's birth weight and adult T2D by fitting a logistic model, treating T2D as outcome and offspring's genetic score as predictor, while controlling for mother's genetic score and other covariates. The adjusted OR for lower birth weight on T2D is estimated to be 1.07 (95% CI 1.04 - 1.11, p = 3.91E-6), again supporting our observation that the lower birth weight is a risk factor of adult diseases. We can only apply this analysis to T2D because we only have individual-level data for T2D in the GERA cohort.

Third, we performed simulations to examine the extent to which the causal effects of

birth weight on adult diseases may be biased by maternal effects. We designed the following simulations (FIGURE S12) following (Lawlor et al., 2017). Let the exposure of birth weight be **x**; let G_o and G_m be the well selected instruments for offspring and mother, respectively; note that, G_o and G_m can be also constructed by (weighted) genetic scores from multiple instrumental variables. Let **y** be the adult disease (**y** can be viewed as the liability value if it is a binary outcome) and U^x be a standard normal random variable that represents the combination of all other intrauterine confounding factors for birth weight. We assume that **x**, G_o, G_m and **y** are all standardized with mean zero and variance one. Under the additive and linear assumptions, the relationship between the offspring exposure (i.e. birth weight) and the instruments is linked with the following model

$$\mathbf{x} = G_o\sqrt{V_o^x} + G_m\sqrt{V_m^x} + U^x\beta_u^x + e, \qquad (23)$$

where $\sqrt{V_o^x}$ and $\sqrt{V_m^x}$ are the corresponding effect sizes for the respective instruments; β_u^x is the effect size of U^x; e is a normal random variable for birth weight with mean zero and variance which is chosen so that **x** has a unit variance. Furthermore, the adult disease **y** is modeled with the following model

$$\mathbf{y} = X\theta^{yx} + G_m\sqrt{V_m^y} + U^y\beta_u^y + \varepsilon, \qquad (24)$$

where θ^{yx} is effect size of exposure **x**, $\sqrt{V_m^y}$ is the effect size; β_u^y is the effect size of confounding factor U^y; ε is a normal random variable for adult disease with mean zero and variance which is selected so that **y** has a unit variance. In the simulations, to mimic the real data in our study we set V_o^x to 0.02, V_m^x to 0.02/10 to emphasize the fact that the PVE by maternal SNPs is much smaller than that by fetal SNPs (Eaves et al., 2014; Horikoshi et al., 2016); we set θ^{yx} to 0.40 (approximately equal to the estimated causal effect of birth weight on T2D in the real data analyses), β_u^x = β_u^y to 0.10. We estimated θ^{yx} by using the two-stage least squares method (2SLS) and examined the influence of parental effects on the causal effect estimation by varying V_m^y (i.e. V_m^y = 10^{-1}, 10^{-2}, 10^{-3}, 10^{-4} or 0). We set the sample size to 10,000 and reported the estimated effect size of θ^{yx} across 1,000 replicates. The simulation framework is summarized in (FIGURE S12).

Finally, we performed direct calculation to estimate bias due to maternal effect confounding with realistic assumption following (Vanderweele and Arah, 2011). Specifically, we denote the observed maternal effect on a given adult disease as ϑ (on the log scale), and denote the probability that a mother gives birth to an offspring with a low birth weight to be π. To simplify bias calculation, we further assume that the maternal effect (i.e. ϑ) is fixed and is not a function of the observed birth weight of the offspring or other unobserved confounders. With these assumptions, the estimation bias due to maternal effect can be expressed as

$$d = \vartheta \times \pi , \tag{25}$$

and the bias corrected causal effect of birth weight on the disease is calculated as θ-d.

Because in practice ϑ is much smaller than the offspring's SNP effect on birth weight (Eaves et al., 2014; Horikoshi et al., 2016) and because π is also small, the estimation bias due to maternal effect is small. Take CAD for example; if we assume that ϑ (i.e. the maternal effect on CAD) is as high as 0.30, which is equivalent to an OR of 1.35, a relatively high assumption; then we need π to be higher than 52.3% (≈ log(1.17)/0.30, here 1.17 is the 95% CI lower limit of the observed causal effect of birth weight for CAD) in order for the maternal effect completely confound our estimates. Similarly, when ϑ is set to be 0.30, the corresponding values of π for MI, T2D and T2D_BMI need to be 40.7%, 46.6% and 74.4%, respectively, in order for a complete confounding of maternal effects. In reality, it has been estimated that the global rate of low birth weight infants only ranges between 15-20% (Cutland et al., 2017): lower in developed regions (e.g. ~6.9% in UK (Johnson et al., 2017)) and higher in developing regions (e.g. ~28% in South Asia (WHO, 2014)). As another way of looking at the bias, if we set ϑ to be 0.30 and π to the global estimates shown above, then we can compute the bias corrected causal effects (OR) of birth weight of offspring on CAD, MI, T2D and T2D_BMI in developing countries (setting π to be 28%) to be 1.23 (95% CI 1.08 - 1.41), 1.20 (95% CI 1.03 - 1.38), 1.30 (95% CI 1.06 - 1.59) and 1.42 (95% CI 1.15~1.74). The corresponding bias corrected causal effects in developed countries (setting π to be 6.3%) are 1.31 (95% CI 1.15 - 1.50), 1.27 (95% CI 1.10 - 1.47), 1.38 (95% CI 1.13 - 1.69) and 1.51 (95% CI 1.23 - 1.85), respectively. And the estimation bias due to maternal effects is estimated to be only 7.87% and 2.16% for developing counties and developed countries, respectively. Therefore, the

bias calculation results suggest that we can still observe relatively unbiased causal effects of birth weight on CAD, MI, T2D and T2D_BMI even in the presence of maternal effect confounding.

Text S5. GWAS summary data source

The GWAS data for advanced age-related macular degeneration (AMD) are obtained from http://csg.sph.umich.edu/abecasis/public/amd2015/. The GWAS Data for Alzheimer's disease is from the International Genomics of Alzheimer's Project (http://web.pasteur-lille.fr). The GWAS data for Parkinson's disease, leptin, adiponectin levels, serum urate, body fat, chronic kidney disease (CKD) are obtained from https://grasp.nhlbi.nih.gov/FullResults.aspx. The GWAS data for ulcerative colitis (UC), inflammatory bowel disease (IBD) and Crohn's disease (CD) are from the International Inflammatory Bowel Disease Genetics Consortium (https://www.ibdgenetics.org/). The GWAS data of primary biliary cirrhosis (PBC), systemic lupus erythematosus (SLE) and celiac disease are obtained from https://www.immunobase.org/downloads/protected_data/GWAS_Data/. The GWAS data of Primary sclerosing cholangitis (PSC) are obtained from http://www.ipscsg.org/. Data on coronary artery disease/myocardial infarction have been contributed by CARDIoGRAMplusC4D investigators and have been downloaded from www.CARDIOGRAMPLUSC4D.ORG. The GWAS data of type 2 diabetes are obtained from the DIAbetes Genetics Replication And Meta-analysis (DIAGRAM) consortium (http://diagram-consortium.org/downloads.html). The summary statistics for ischaemic stroke from the METASTROKE Collaboration at http://cerebrovascularportal.org. The GWAS data of educational attainment are obtained from https://www.thessgac.org/. The GWAS data of smoking behaviors (cigarettes smoked per day, smoking initiation, age of smoking initiation and smoking cessation) are obtained from the Tobacco and Genetics Consortium (http://www.med.unc.edu/pgc/results-and-downloads). The GWAS data of Early Growth (childhood body mass index, Growth_10_12, Growth_PG, Growth_PT, Tanner stage, birth length, childhood obesity and head circumference) are obtained from the Early Growth Genetics Consortium (http://egg-consortium.org/). The GWAS data of lipids (high density lipoproteins, low density lipoproteins, total cholesterol and triglycerides) are obtained from the Global Lipids Genetics Consortium (http://csg.sph.umich.edu/abecasis/public/lipids2010/). The GWAS data of glycaemic traits (HOMA-B, HOMA-IR, fasting glucose, fasting insulin, glucose levels 2 hours after an oral glucose and modified stumvoll insulin sensitivity index) are obtained from the Meta-Analyses of Glucose and Insulin-related traits Consortium (MAGIC)

(https://www.magicinvestigators.org/). The GWAS data of anthropometric traits (waist-to-hip ratio, waist circumference, hip circumference, body mass index, height, overweight and obesity) are obtained from the Genetic Investigation of ANthropometric Traits (GIANT) consortium (http://portals.broadinstitute.org/collaboration/giant/index.php/GIANT_consortium_data_files). The Framingham heart study (FHS) genotype and phenotype data is available in dbGaP (https://www.ncbi.nlm.nih.gov/gap) with accession number phs000007. The Atherosclerosis Risk in Communities (ARIC) genotype and phenotype data is available in dbGaP (https://www.ncbi.nlm.nih.gov/gap) with accession number phs000090. This study also makes use of data generated by the Wellcome Trust Case Control Consortium (WTCCC). A full list of the investigators who contributed to the generation of the data is available from http://www.wtccc.org.uk. Funding for the WTCCC project was provided by the Wellcome Trust under award 076113 and 085475. The GERA Data came from a grant, the Resource for Genetic Epidemiology Research in Adult Health and Aging (RC2 AG033067; Schaefer and Risch, PIs) awarded to the Kaiser Permanente Research Program on Genes, Environment, and Health (RPGEH) and the UCSF Institute for Human Genetics. The RPGEH was supported by grants from the Robert Wood Johnson Foundation, the Wayne and Gladys Valley Foundation, the Ellison Medical Foundation, Kaiser Permanente Northern California, and the Kaiser Permanente National and Northern California Community Benefit Programs. The RPGEH and the Resource for Genetic Epidemiology Research in Adult Health and Aging are described in the following publication, Schaefer C, et al., The Kaiser Permanente Research Program on Genes, Environment and Health: Development of a Research Resource in a Multi-Ethnic Health Plan with Electronic Medical Records, in preparation, 2013. The UK Biobank data sets can be available from http://www.nealelab.is/uk-Biobank/.

References

1. Afzal, S., Brøndum-Jacobsen, P., Bojesen, S.E., and Nordestgaard, B.G. (2014). Vitamin D concentration, obesity, and risk of diabetes: a mendelian randomisation study. *Lancet Diabetes Endo* 2(4), 298-306. doi: 10.1016/s2213-8587(13)70200-6.
2. Aikens, R.C., Zhao, W., Saleheen, D., Reilly, M.P., Epstein, S.E., Tikkanen, E., et al. (2017). Systolic Blood Pressure and Risk of Type 2 Diabetes: A Mendelian Randomization Study. *Diabetes* 66(2), 543-550. doi: 10.2337/db16-0868.
3. Baker , J.L., Olsen , L.W., and Sørensen , T.I.A. (2007). Childhood Body-Mass Index and the Risk of Coronary Heart Disease in Adulthood. *New England Journal of Medicine* 357(23), 2329-2337. doi: 10.1056/NEJMoa072515.
4. Balding, D.J. (2006). A tutorial on statistical methods for population association studies. *Nature Reviews. Genetics* 7(10), 781-791. doi: 10.1038/nrg1916.
5. Banda, Y., Kvale, M.N., Hoffmann, T.J., Hesselson, S.E., Ranatunga, D., Tang, H., et al. (2015). Characterizing race/ethnicity and genetic ancestry for 100,000 subjects in the Genetic Epidemiology Research on Adult Health and Aging (GERA) cohort. *Genetics* 200(4), 1285-1295.
6. Beaumont, R.N., Warrington, N.M., Cavadino, A., Tyrrell, J., Nodzenski, M., Horikoshi, M., et al. (2018). Genome-wide association study of offspring birth weight in 86 577 women identifies five novel loci and highlights maternal genetic effects that are independent of fetal genetics. *Human Molecular Genetics* 27(4), 742-756. doi: 10.1093/hmg/ddx429.
7. Bentham, J., Morris, D.L., Cunninghame Graham, D.S., Pinder, C.L., Tombleson, P., Behrens, T.W., et al. (2015). Genetic association analyses implicate aberrant regulation of innate and adaptive immunity genes in the pathogenesis of systemic lupus erythematosus. *Nature Genetics* 47(12), 1457-1464. doi: 10.1038/ng.3434.
8. Berndt, S.I., Gustafsson, S., Magi, R., Ganna, A., Wheeler, E., Feitosa, M.F., et al. (2013). Genome-wide meta-analysis identifies 11 new loci for anthropometric traits and provides insights into genetic architecture. *Nature Genetics* 45(5), 501-512. doi: 10.1038/ng.2606.
9. Borges, M.C., Lawlor, D.A., de Oliveira, C., White, J., Horta, B., and Barros, A.J. (2016). The role of adiponectin in coronary heart disease risk: a Mendelian randomization study. *Circulation Research* 119(3), 491-499. doi: 10.1161/CIRCRESAHA.116.308716.
10. Bound, J., Jaeger, D.A., and Baker, R.M. (1995). Problems with instrumental variables estimation when the correlation between the instruments and the endogenous explanatory variable is weak. *Journal of the American Statistical Association* 90(430), 443-450. doi: 10.2307/2291055.
11. Bowden, J., Davey Smith, G., and Burgess, S. (2015). Mendelian randomization with invalid instruments: effect estimation and bias detection through Egger regression. *International Journal of Epidemiology* 44(2), 512-525.
12. Bowden, J., Davey Smith, G., Haycock, P.C., and Burgess, S. (2016a). Consistent estimation in Mendelian randomization with some invalid

instruments using a weighted median estimator. *Genetic Epidemiology* 40(4)**,** 304-314. doi: 10.1002/gepi.21965.
13. Bowden, J., Del Greco M, F., Minelli, C., Davey Smith, G., Sheehan, N.A., and Thompson, J.R. (2016b). Assessing the suitability of summary data for two-sample Mendelian randomization analyses using MR-Egger regression: the role of the I^2 statistic. *International Journal of Epidemiology* 45(6)**,** 1961-1974. doi: 10.1093/ije/dyw220.
14. Bradfield, J.P., Taal, H.R., Timpson, N.J., Scherag, A., Lecoeur, C., Warrington, N.M., et al. (2012). A genome-wide association meta-analysis identifies new childhood obesity loci. *Nature Genetics* 44(5)**,** 526-531. doi: 10.1038/ng.2247.
15. Brion, M.-J.A., Shakhbazov, K., and Visscher, P.M. (2013). Calculating statistical power in Mendelian randomization studies. *International Journal of Epidemiology* 42(5)**,** 1497-1501. doi: 10.1093/ije/dyt179.
16. Brockwell, S.E., and Gordon, I.R. (2001). A comparison of statistical methods for meta-analysis. *Statistics in Medicine* 20(6)**,** 825-840. doi: 10.1002/sim.650.
17. Burgess, S. (2014). Sample size and power calculations in Mendelian randomization with a single instrumental variable and a binary outcome. *International Journal of Epidemiology* 43**,** 922-929.
18. Burgess, S., Bowden, J., Fall, T., Ingelsson, E., and Thompson, S.G. (2017a). Sensitivity analyses for robust causal inference from mendelian randomization analyses with multiple genetic variants. *Epidemiology* 28(1)**,** 30-42.
19. Burgess, S., Small, D.S., and Thompson, S.G. (2017b). A review of instrumental variable estimators for Mendelian randomization. *Statistical Methods in Medical Research* 26(5)**,** 2333-2355. doi: 10.1177/0962280215597579
20. Burgess, S., Thompson, D.J., Rees, J.M.B., Day, F.R., Perry, J.R., and Ong, K.K. (2017c). Dissecting Causal Pathways Using Mendelian Randomization with Summarized Genetic Data: Application to Age at Menarche and Risk of Breast Cancer. *Genetics* 207(2)**,** 481-487. doi: 10.1534/genetics.117.300191.
21. Burgess, S., and Thompson, S.G. (2011). Avoiding bias from weak instruments in Mendelian randomization studies. *International Journal of Epidemiology* 40(3)**,** 755-764. doi: 10.1093/ije/dyr036.
22. Burgess, S., and Thompson, S.G. (2012). Improving bias and coverage in instrumental variable analysis with weak instruments for continuous and binary outcomes. *Statistics in Medicine* 31(15)**,** 1582-1600. doi: 10.1002/sim.4498.
23. Burgess, S., and Thompson, S.G. (2015). Multivariable Mendelian Randomization: The Use of Pleiotropic Genetic Variants to Estimate Causal Effects. *American Journal of Epidemiology* 181(4)**,** 251-260. doi: 10.1093/aje/kwu283.
24. Burgess, S., and Thompson, S.G. (2017). Interpreting findings from Mendelian randomization using the MR-Egger method. *European Journal of Epidemiology* 32(5)**,** 377-389. doi: 10.1007/s10654-017-0255-x.
25. Burton, P.R., Clayton, D.G., Cardon, L.R., Craddock, N., Deloukas, P., Duncanson, A., et al. (2007). Association scan of 14,500 nonsynonymous SNPs in four diseases identifies autoimmunity variants. *Nature Genetics* 39(11)**,**

1329-1337. doi: 10.1038/ng.2007.17.

26. Carreras-Torres, R., Johansson, M., Gaborieau, V., Haycock, P.C., Wade, K.H., Relton, C.L., et al. (2017). The role of obesity, type 2 diabetes, and metabolic factors in pancreatic cancer: A Mendelian randomization study. *Journal of the National Cancer Institute* 109(9). doi: 10.1093/jnci/djx012
27. Censin, J.C., Nowak, C., Cooper, N., Bergsten, P., Todd, J.A., and Fall, T. (2017). Childhood adiposity and risk of type 1 diabetes: A Mendelian randomization study. *PLoS Medicine* 14(8)**,** e1002362. doi: 10.1371/journal.pmed.1002362.
28. Chomitz, V.R., Cheung, L.W., and Lieberman, E. (1995). The role of lifestyle in preventing low birth weight. *Future Child* 5(1)**,** 121-138. doi: 10.2307/1602511.
29. Collier, S.A., and Hogue, C.J.R. (2007). Modifiable Risk Factors for Low Birth Weight and Their Effect on Cerebral Palsy and Mental Retardation. *Maternal and Child Health Journal* 11(1)**,** 65-71. doi: 10.1007/s10995-006-0085-z.
30. Conde-Agudelo, A., Rosas-Bermúdez, A., and Kafury-Goeta, A. (2006). Birth spacing and risk of adverse perinatal outcomes: A meta-analysis. *JAMA* 295(15)**,** 1809-1823. doi: 10.1001/jama.295.15.1809.
31. Corbin, L.J., Richmond, R.C., Wade, K.H., Burgess, S., Bowden, J., Smith, G.D., et al. (2016). Body mass index as a modifiable risk factor for type 2 diabetes: Refining and understanding causal estimates using Mendelian randomisation. *Diabetes* 65(10)**,** 3002-3007. doi: 10.2337/db16-0418.
32. Cordell, H.J., Han, Y., Mells, G.F., Li, Y., Hirschfield, G.M., Greene, C.S., et al. (2015). International genome-wide meta-analysis identifies new primary biliary cirrhosis risk loci and targetable pathogenic pathways. *Nature Communications* 6**,** 8019. doi: 10.1038/ncomms9019.
33. Cousminer, D.L., Berry, D.J., Timpson, N.J., Ang, W., Thiering, E., Byrne, E.M., et al. (2013). Genome-wide association and longitudinal analyses reveal genetic loci linking pubertal height growth, pubertal timing and childhood adiposity. *Human Molecular Genetics* 22(13)**,** 2735-2747. doi: 10.1093/hmg/ddt104.
34. Cousminer, D.L., Stergiakouli, E., Berry, D.J., Ang, W., Groen-Blokhuis, M.M., Körner, A., et al. (2014). Genome-wide association study of sexual maturation in males and females highlights a role for body mass and menarche loci in male puberty. *Human Molecular Genetics* 23(16)**,** 4452-4464. doi: 10.1093/hmg/ddu150.
35. Cragg, J.G., and Donald, S.G. (1993). Testing Identifiability and Specification in Instrumental Variable Models. *Econometric Theory* 9(2)**,** 222-240. doi: 10.1017/s0266466600007519.
36. Cutland, C.L., Lackritz, E.M., Mallett-Moore, T., Bardají, A., Chandrasekaran, R., Lahariya, C., et al. (2017). Low birth weight: Case definition & guidelines for data collection, analysis, and presentation of maternal immunization safety data. *Vaccine* 35(48, Part A)**,** 6492-6500. doi: https://doi.org/10.1016/j.vaccine.2017.01.049.
37. Das, S., Forer, L., Schonherr, S., Sidore, C., Locke, A.E., Kwong, A., et al. (2016). Next-generation genotype imputation service and methods. *Nature Genetics* 48(10)**,** 1284-1287. doi: 10.1038/ng.3656.

38. Dastani, Z., Hivert, M.-F., Timpson, N., Perry, J.R.B., Yuan, X., Scott, R.A., et al. (2012). Novel Loci for Adiponectin Levels and Their Influence on Type 2 Diabetes and Metabolic Traits: A Multi-Ethnic Meta-Analysis of 45,891 Individuals. *PLoS Genetics* 8(3), e1002607. doi: 10.1371/journal.pgen.1002607.
39. Delaneau, O., Howie, B., Cox, A.J., Zagury, J.F., and Marchini, J. (2013a). Haplotype estimation using sequencing reads. *American Journal of Human Genetics* 93(4), 687-696. doi: 10.1016/j.ajhg.2013.09.002.
40. Delaneau, O., Marchini, J., and Zagury, J.F. (2012). A linear complexity phasing method for thousands of genomes. *Nature Methods* 9(2), 179-181. doi: 10.1038/nmeth.1785.
41. Delaneau, O., Zagury, J.F., and Marchini, J. (2013b). Improved whole-chromosome phasing for disease and population genetic studies. *Nature Methods* 10(1), 5-6. doi: 10.1038/nmeth.2307.
42. DerSimonian, R., and Laird, N. (1986). Meta-analysis in clinical trials. *Controlled Clinical Trials* 7(3), 177-188. doi: 10.1016/0197-2456(86)90046-2.
43. Dixon, S.C., Nagle, C.M., Thrift, A.P., Pharoah, P.D., Pearce, C.L., Zheng, W., et al. (2016). Adult body mass index and risk of ovarian cancer by subtype: a Mendelian randomization study. *International Journal of Epidemiology* 45(3), 884-895.
44. Do, R., Willer, C.J., Schmidt, E.M., Sengupta, S., Gao, C., Peloso, G.M., et al. (2013). Common variants associated with plasma triglycerides and risk for coronary artery disease. *Nature Genetics* 45(11), 1345-1352. doi: 10.1038/ng.2795.
45. Dubois, P.C.A., Trynka, G., Franke, L., Hunt, K.A., Romanos, J., Curtotti, A., et al. (2010). Multiple common variants for celiac disease influencing immune gene expression. *Nature Genetics* 42(4), 295-302. doi: 10.1038/ng.543.
46. Dupuis, J., Langenberg, C., Prokopenko, I., Saxena, R., Soranzo, N., Jackson, A.U., et al. (2010). New genetic loci implicated in fasting glucose homeostasis and their impact on type 2 diabetes risk. *Nature Genetics* 42(2), 105-116. doi: 10.1038/ng.520.
47. Eaves, L.J., Pourcain, B.S., Smith, G.D., York, T.P., and Evans, D.M. (2014). Resolving the effects of maternal and offspring genotype on dyadic outcomes in genome wide complex trait analysis ("M-GCTA"). *Behavior Genetics* 44(5), 445-455.
48. Emdin, C.A., Khera, A.V., Natarajan, P., and et al. (2017). Genetic association of waist-to-hip ratio with cardiometabolic traits, type 2 diabetes, and coronary heart disease. *JAMA* 317(6), 626-634. doi: 10.1001/jama.2016.21042.
49. Fall, T., Hägg, S., Mägi, R., Ploner, A., Fischer, K., Horikoshi, M., et al. (2013). The Role of Adiposity in Cardiometabolic Traits: A Mendelian Randomization Analysis. *PLoS Medicine* 10(6), e1001474. doi: 10.1371/journal.pmed.1001474.
50. Felix, J.F., Bradfield, J.P., Monnereau, C., van der Valk, R.J.P., Stergiakouli, E., Chesi, A., et al. (2016). Genome-wide association analysis identifies three new susceptibility loci for childhood body mass index. *Human Molecular Genetics* 25(2), 389-403. doi: 10.1093/hmg/ddv472.

51. Frayling, T.M., Timpson, N.J., Weedon, M.N., Zeggini, E., Freathy, R.M., Lindgren, C.M., et al. (2007). A Common Variant in the FTO Gene Is Associated with Body Mass Index and Predisposes to Childhood and Adult Obesity. *Science* 316(5826), 889-894. doi: 10.1126/science.1141634.
52. Freeman, G., Cowling, B.J., and Schooling, C.M. (2013). Power and sample size calculations for Mendelian randomization studies using one genetic instrument. *International Journal of Epidemiology* 42(4), 1157-1163.
53. Fritsche, L.G., Igl, W., Bailey, J.N.C., Grassmann, F., Sengupta, S., Bragg-Gresham, J.L., et al. (2016). A large genome-wide association study of age-related macular degeneration highlights contributions of rare and common variants. *Nature Genetics* 48(2), 134-143. doi: 10.1038/ng.3448.
54. Guan, Y., and Stephens, M. (2008). Practical Issues in Imputation-Based Association Mapping. *PLoS Genetics* 4(12), e1000279. doi: 10.1371/journal.pgen.1000279.
55. Hartwig, F.P., Davey Smith, G., and Bowden, J. (2017). Robust inference in summary data Mendelian randomization via the zero modal pleiotropy assumption. *International Journal of Epidemiology* 46(6), 1985-1998. doi: 10.1093/ije/dyx102
56. Hemani, G., Bowden, J., and Davey Smith, G. (2018). Evaluating the potential role of pleiotropy in Mendelian randomization studies. *Human Molecular Genetics*. doi: 10.1093/hmg/ddy163.
57. Hindy, G., Engström, G., Larsson, S.C., Traylor, M., Markus, H.S., Melander, O., et al. (2018). Role of Blood Lipids in the Development of Ischemic Stroke and its Subtypes. *A Mendelian Randomization Study* 49(4), 820-827. doi: 10.1161/strokeaha.117.019653.
58. Holmes, Michael V., Lange, Leslie A., Palmer, T., Lanktree, Matthew B., North, Kari E., Almoguera, B., et al. (2014a). Causal Effects of Body Mass Index on Cardiometabolic Traits and Events: A Mendelian Randomization Analysis. *The American Journal of Human Genetics* 94(2), 198-208. doi: http://dx.doi.org/10.1016/j.ajhg.2013.12.014.
59. Holmes, M.V., Lange, L.A., Palmer, T., Lanktree, M.B., North, K.E., Almoguera, B., et al. (2014b). Causal Effects of Body Mass Index on Cardiometabolic Traits and Events: A Mendelian Randomization Analysis. *American Journal of Human Genetics* 94(2), 198-208. doi: 10.1016/j.ajhg.2013.12.014.
60. Horikoshi, M., Beaumont, R.N., Day, F.R., Warrington, N.M., Kooijman, M.N., Fernandez-Tajes, J., et al. (2016). Genome-wide associations for birth weight and correlations with adult disease. *Nature* 538(7624), 248-252. doi: 10.1038/nature19806.
61. Horikoshi, M., Yaghootkar, H., Mook-Kanamori, D.O., Sovio, U., Taal, H.R., Hennig, B.J., et al. (2013). New loci associated with birth weight identify genetic links between intrauterine growth and adult height and metabolism. *Nature Genetics* 45(1), 76-82. doi: 10.1038/ng.2477.
62. Howie, B.N., Donnelly, P., and Marchini, J. (2009). A Flexible and Accurate Genotype Imputation Method for the Next Generation of Genome-Wide

Association Studies. *PLoS Genetics* 5(6)**,** e1000529. doi: 10.1371/journal.pgen.1000529.
63. Ji, S.-G., Juran, B.D., Mucha, S., Folseraas, T., Jostins, L., Melum, E., et al. (2017a). Genome-wide association study of primary sclerosing cholangitis identifies new risk loci and quantifies the genetic relationship with inflammatory bowel disease. *Nature Genetics* 49(2)**,** 269-273. doi: 10.1038/ng.3745.
64. Ji, S.-G., Juran, B.D., Mucha, S., Folseraas, T., Jostins, L., Melum, E., et al. (2017b). Genome-wide association study of primary sclerosing cholangitis identifies new risk loci and quantifies the genetic relationship with inflammatory bowel disease. *Nature Genetics* 49(2)**,** 269-273. doi: 10.1038/ng.3745.
65. Johnson, C.D., Jones, S., and Paranjothy, S. (2017). Reducing low birth weight: prioritizing action to address modifiable risk factors. *Journal of Public Health* 39(1)**,** 122-131. doi: 10.1093/pubmed/fdv212.
66. Köttgen, A., Pattaro, C., Böger, C.A., Fuchsberger, C., Olden, M., Glazer, N.L., et al. (2010). New loci associated with kidney function and chronic kidney disease. *Nature Genetics* 42(5)**,** 376-384. doi: 10.1038/ng.568.
67. Keele, L., and Morgan, J.W. (2016). How strong is strong enough? Strengthening instruments through matching and weak instrument tests. *Annals of Applied Statistics* (2)**,** 1086-1106. doi: 10.1214/16-aoas932.
68. Kilpeläinen, T.O., Carli, J.F.M., Skowronski, A.A., Sun, Q., Kriebel, J., Feitosa, M.F., et al. (2016). Genome-wide meta-analysis uncovers novel loci influencing circulating leptin levels. *Nature Communications* 7**,** 10494. doi: 10.1038/ncomms10494.
69. Kottgen, A., Albrecht, E., Teumer, A., Vitart, V., Krumsiek, J., Hundertmark, C., et al. (2013). Genome-wide association analyses identify 18 new loci associated with serum urate concentrations. *Nature Genetics* 45(2)**,** 145-154. doi: 10.1038/ng.2500.
70. Lambert, J.-C., Ibrahim-Verbaas, C.A., Harold, D., Naj, A.C., Sims, R., Bellenguez, C., et al. (2013). Meta-analysis of 74,046 individuals identifies 11 new susceptibility loci for Alzheimer's disease. *Nature Genetics* 45(12)**,** 1452-1458. doi: 10.1038/ng.2802.
71. Larsson Susanna, C., Traylor, M., Mishra, A., Howson Joanna, M.M., Michaëlsson, K., Markus Hugh, S., et al. (2018). Serum 25-Hydroxyvitamin D Concentrations and Ischemic Stroke and Its Subtypes. *Stroke* 49(10)**,** 2508-2511. doi: 10.1161/strokeaha.118.022242.
72. Lawlor, D., Richmond, R., Warrington, N., McMahon, G., Davey Smith, G., Bowden, J., et al. (2017). Using Mendelian randomization to determine causal effects of maternal pregnancy (intrauterine) exposures on offspring outcomes: Sources of bias and methods for assessing them [version 1; referees: 4 approved]. *Wellcome Open Research* 2**,** 11. doi: 10.12688/wellcomeopenres.10567.1.
73. Lawlor, D.A., Harbord, R.M., Sterne, J.A., Timpson, N., and Davey Smith, G. (2008a). Mendelian randomization: using genes as instruments for making causal inferences in epidemiology. *Statistics in Medicine* 27(8)**,** 1133-1163. doi:

10.1002/sim.3034.
74. Lawlor, D.A., Timpson, N.J., Harbord, R.M., Leary, S., Ness, A., McCarthy, M.I., et al. (2008b). Exploring the Developmental Overnutrition Hypothesis Using Parental–Offspring Associations and FTO as an Instrumental Variable. *PLoS Medicine* 5(3)**,** e33. doi: 10.1371/journal.pmed.0050033.
75. Lee, T., and Pickard, A. (2013). "Exposure Definition and Measurement," in *Developing a Protocol for Observational Comparative Effectiveness Research: A User's Guide*, eds. P. Velentgas, N. Dreyer & P. Nourjah. (Rockville (MD): Agency for Healthcare Research and Quality (US)), Available from: https://www.ncbi.nlm.nih.gov/books/NBK126191/.
76. Liu, J.Z., van Sommeren, S., Huang, H., Ng, S.C., Alberts, R., Takahashi, A., et al. (2015). Association analyses identify 38 susceptibility loci for inflammatory bowel disease and highlight shared genetic risk across populations. *Nature Genetics* 47(9)**,** 979-986. doi: 10.1038/ng.3359.
77. Locke, A.E., Kahali, B., Berndt, S.I., Justice, A.E., Pers, T.H., Day, F.R., et al. (2015). Genetic studies of body mass index yield new insights for obesity biology. *Nature* 518(7538)**,** 197-206. doi: 10.1038/nature14177.
78. Lu, Y.C., Day, F.R., Gustafsson, S., Buchkovich, M.L., Na, J.B., Bataille, V., et al. (2016). New loci for body fat percentage reveal link between adiposity and cardiometabolic disease risk. *Nature Communications* 7(1)**,** 10495. doi: 10.1038/ncomms10495.
79. Lyall, D.M., Celis-Morales, C., Ward, J., and et al. (2017). Association of body mass index with cardiometabolic disease in the uk biobank: A mendelian randomization study. *JAMA Cardiology* 2(8)**,** 882-889. doi: 10.1001/jamacardio.2016.5804.
80. Lyall, D.M., Celis-Morales, C., Ward, J., Iliodromiti, S., Anderson, J.J., Gill, J.M.R., et al. (2016). Body-mass index and cardiometabolic disease: a Mendelian randomisation study of UK Biobank participants. *Lancet* 388**,** S9. doi: 10.1016/s0140-6736(16)32245-0.
81. Malik, R., Chauhan, G., Traylor, M., Sargurupremraj, M., Okada, Y., Mishra, A., et al. (2018). Multiancestry genome-wide association study of 520,000 subjects identifies 32 loci associated with stroke and stroke subtypes. *Nature Genetics* 50(4)**,** 524-537. doi: 10.1038/s41588-018-0058-3.
82. McCarthy, S., Das, S., Kretzschmar, W., Delaneau, O., Wood, A.R., Teumer, A., et al. (2016). A reference panel of 64,976 haplotypes for genotype imputation. *Nature Genetics* 48(10)**,** 1279-1283. doi: 10.1038/ng.3643.
83. Mendelson, M.M., Marioni, R.E., Joehanes, R., Liu, C., Hedman, Å.K., Aslibekyan, S., et al. (2017). Association of Body Mass Index with DNA Methylation and Gene Expression in Blood Cells and Relations to Cardiometabolic Disease: A Mendelian Randomization Approach. *PLoS Medicine* 14(1)**,** e1002215. doi: 10.1371/journal.pmed.1002215.
84. Nelson, C.P., Hamby, S.E., Saleheen, D., Hopewell, J.C., Zeng, L., Assimes, T.L., et al. (2015). Genetically Determined Height and Coronary Artery Disease. *New England Journal of Medicine* 372(17)**,** 1608-1618. doi:

doi:10.1056/NEJMoa1404881.
85. Nikpay, M., Goel, A., Won, H.-H., Hall, L.M., Willenborg, C., Kanoni, S., et al. (2015). A comprehensive 1000 Genomes-based genome-wide association meta-analysis of coronary artery disease. *Nature Genetics* 47(10)**,** 1121-1130. doi: 10.1038/ng.3396.
86. Noyce, A.J., Kia, D.A., Hemani, G., Nicolas, A., Price, T.R., De Pablo-Fernandez, E., et al. (2017). Estimating the causal influence of body mass index on risk of Parkinson disease: A Mendelian randomisation study. *PLoS Medicine* 14(6)**,** e1002314. doi: 10.1371/journal.pmed.1002314.
87. Østergaard, S.D., Mukherjee, S., Sharp, S.J., Proitsi, P., Lotta, L.A., Day, F., et al. (2015). Associations between Potentially Modifiable Risk Factors and Alzheimer Disease: A Mendelian Randomization Study. *PLoS Medicine* 12(6)**,** e1001841. doi: 10.1371/journal.pmed.1001841.
88. Pankratz, N., Beecham, G.W., DeStefano, A.L., Dawson, T.M., Doheny, K.F., Factor, S.A., et al. (2012). Meta-analysis of Parkinson's Disease: Identification of a novel locus, RIT2. *Annals of Neurology* 71(3)**,** 370-384. doi: 10.1002/ana.22687.
89. Pare, G., and Anand, S.S. (2010). Mendelian randomisation, triglycerides, and CHD. *Lancet* 375(9726)**,** 1584-1586. doi: 10.1016/S0140-6736(10)60659-9.
90. Pasaniuc, B., and Price, A.L. (2017). Dissecting the genetics of complex traits using summary association statistics. *Nature Reviews. Genetics* 18(2)**,** 117-127. doi: 10.1038/nrg.2016.142.
91. Pasaniuc, B., Zaitlen, N., Shi, H., Bhatia, G., Gusev, A., Pickrell, J., et al. (2014). Fast and accurate imputation of summary statistics enhances evidence of functional enrichment. *Bioinformatics* 30(20)**,** 2906-2914. doi: 10.1093/bioinformatics/btu416.
92. Pickrell, J.K. (2014). Joint analysis of functional genomic data and genome-wide association studies of 18 human traits. *American Journal of Human Genetics* 94(4)**,** 559-573.
93. Pickrell, J.K., Berisa, T., Liu, J.Z., Segurel, L., Tung, J.Y., and Hinds, D.A. (2016). Detection and interpretation of shared genetic influences on 42 human traits. *Nature Genetics* 48(7)**,** 709-717. doi: 10.1038/ng.3570.
94. Purcell, S., Neale, B., Todd-Brown, K., Thomas, L., Ferreira, M.A.R., Bender, D., et al. (2007). PLINK: A Tool Set for Whole-Genome Association and Population-Based Linkage Analyses. *American Journal of Human Genetics* 81(3)**,** 559-575. doi: 10.1086/519795.
95. Richey Sharrett, A. (1992). The atherosclerosis risk in communities (ARIL) study introduction and objectives of the hemostasis component. *Annals of Epidemiology* 2(4)**,** 467-469. doi: 10.1016/1047-2797(92)90096-9.
96. Rietveld, C.A., Medland, S.E., Derringer, J., Yang, J., Esko, T., Martin, N.W., et al. (2013). GWAS of 126,559 individuals identifies genetic variants associated with educational attainment. *Science* 340(6139)**,** 1467-1471. doi: 10.1126/science.1235488.
97. Rogowski, J. (1998). Cost-effectiveness of care for very low birth weight infants.

Pediatrics 102(1), 35-43. doi: 10.1542/peds.102.1.35.
98. Saxena, R., Hivert, M.-F., Langenberg, C., Tanaka, T., Pankow, J.S., Vollenweider, P., et al. (2010). Genetic variation in GIPR influences the glucose and insulin responses to an oral glucose challenge. *Nature Genetics* 42(2), 142-148. doi: 10.1038/ng.521.
99. Scott, R.A., Scott, L.J., Mägi, R., Marullo, L., Gaulton, K.J., Kaakinen, M., et al. (2017). An Expanded Genome-Wide Association Study of Type 2 Diabetes in Europeans. *Diabetes* 66(11), 2888-2902. doi: 10.2337/db16-1253.
100. Sheehan, N.A., Didelez, V., Burton, P.R., and Tobin, M.D. (2008). Mendelian randomisation and causal inference in observational epidemiology. *PLoS Medicine* 5(8), e177. doi: 10.1371/journal.pmed.0050177.
101. Shungin, D., Winkler, T.W., Croteau-Chonka, D.C., Ferreira, T., Locke, A.E., Mägi, R., et al. (2015). New genetic loci link adipose and insulin biology to body fat distribution. *Nature* 518(7538), 187-196. doi: 10.1038/nature14132.
102. Splansky, G.L., Corey, D., Yang, Q., Atwood, L.D., Cupples, L.A., and Benjamin, E.J. (2007). The Third Generation Cohort of the National Heart, Lung, and Blood Institute's Framingham Heart Study: design, recruitment, and initial examination. *American Journal of Epidemiology* 165(11), 1328-1335. doi: 10.1093/aje/kwm021.
103. Staiger, D., and Stock, J.H. (1997). Instrumental variables regression with weak instruments. *Econometrica* 65(3), 557-586. doi: 10.2307/2171753.
104. Sudlow, C., Gallacher, J., Allen, N., Beral, V., Burton, P., Danesh, J., et al. (2015). UK Biobank: An Open Access Resource for Identifying the Causes of a Wide Range of Complex Diseases of Middle and Old Age. *PLoS Medicine* 12(3), e1001779. doi: 10.1371/journal.pmed.1001779.
105. Taal, H.R., St Pourcain, B., Thiering, E., Das, S., Mook-Kanamori, D.O., Warrington, N.M., et al. (2012). Common variants at 12q15 and 12q24 are associated with infant head circumference. *Nature Genetics* 44(5), 532-538. doi: 10.1038/ng.2238.
106. Teslovich, T.M., Musunuru, K., Smith, A.V., Edmondson, A.C., Stylianou, I.M., Koseki, M., et al. (2010). Biological, clinical and population relevance of 95 loci for blood lipids. *Nature* 466(7307), 707-713. doi: 10.1038/nature09270.
107. The 1000 Genomes Project Consortium (2015). A global reference for human genetic variation. *Nature* 526(7571), 68-74. doi: 10.1038/nature15393
108. http://www.nature.com/nature/journal/v526/n7571/abs/nature15393.html#supplementary-information.
109. The International Consortium for Blood Pressure Genome-Wide Association Studies. (2011). Genetic variants in novel pathways influence blood pressure and cardiovascular disease risk. *Nature* 478(7367), 103-109. doi: 10.1038/nature10405.
110. The Tobacco and Genetics Consortium (2010). Genome-wide meta-analyses identify multiple loci associated with smoking behavior. *Nature Genetics* 42(5), 441-447. doi: 10.1038/ng.571.
111. The Wellcome Trust Case Control Consortium (2007). Genome-wide

association study of 14,000 cases of seven common diseases and 3,000 shared controls. *Nature* 447(7145)**,** 661-678. doi: 10.1038/nature05911.

112. Thomas, D.C., Lawlor, D.A., and Thompson, J.R. (2007). re: Estimation of bias in nongenetic observational studies using “Mendelian triangulation” by Bautista et al. *Annals of Epidemiology* 17(7)**,** 511-513. doi: 10.1016/j.annepidem.2006.12.005.

113. Thompson, S.G., and Sharp, S.J. (1999). Explaining heterogeneity in meta-analysis: A comparison of methods. *Statistics in Medicine* 18(20)**,** 2693-2708. doi: 10.1002/(sici)1097-0258(19991030)18:20<2693::aid-sim235>3.0.co;2-v.

114. Tillmann, T., Vaucher, J., Okbay, A., Pikhart, H., Peasey, A., Kubinova, R., et al. (2017). Education and coronary heart disease: mendelian randomisation study. *British Medical Journal* 358**,** j3542. doi: 10.1136/bmj.j3542.

115. Triglyceride Coronary Disease Genetics Consortium and Emerging Risk Factors Collaboration. (2010). Triglyceride-mediated pathways and coronary disease: collaborative analysis of 101 studies. *Lancet* 375(9726)**,** 1634-1639. doi: 10.1016/S0140-6736(10)60545-4.

116. van der Valk, R.J.P., Kreiner-Møller, E., Kooijman, M.N., Guxens, M., Stergiakouli, E., Sääf, A., et al. (2015). A novel common variant in DCST2 is associated with length in early life and height in adulthood. *Human Molecular Genetics* 24(4)**,** 1155-1168. doi: 10.1093/hmg/ddu510.

117. Vanderweele, T.J., and Arah, O.A. (2011). Bias formulas for sensitivity analysis of unmeasured confounding for general outcomes, treatments, and confounders. *Epidemiology* 22(1)**,** 42-52. doi: 10.1097/EDE.0b013e3181f74493.

118. Verbanck, M., Chen, C.-Y., Neale, B., and Do, R. (2018). Detection of widespread horizontal pleiotropy in causal relationships inferred from Mendelian randomization between complex traits and diseases. *Nature Genetics* 50(5)**,** 693-698. doi: 10.1038/s41588-018-0099-7.

119. Voight, B.F., Peloso, G.M., Orho-Melander, M., Frikke-Schmidt, R., Barbalic, M., Jensen, M.K., et al. (2012). Plasma HDL cholesterol and risk of myocardial infarction: a mendelian randomisation study. *Lancet* 380(9841)**,** 572-580. doi: 10.1016/S0140-6736(12)60312-2.

120. Walford, G.A., Gustafsson, S., Rybin, D., Stančáková, A., Chen, H., Liu, C.-T., et al. (2016). Genome-Wide Association Study of the Modified Stumvoll Insulin Sensitivity Index Identifies BCL2 and FAM19A2 as Novel Insulin Sensitivity Loci. *Diabetes* 65(10)**,** 3200-3211. doi: 10.2337/db16-0199.

121. Warrington, N.M., Freathy, R.M., Neale, M.C., and Evans, D.M. (2018). Using structural equation modelling to jointly estimate maternal and fetal effects on birthweight in the UK Biobank. *International Journal of Epidemiology***,** dyy015-dyy015. doi: 10.1093/ije/dyy015.

122. White, J., Swerdlow, D.I., Preiss, D., Fairhurst-Hunter, Z., Keating, B.J., Asselbergs, F.W., et al. (2016). Association of lipid fractions with risks for coronary artery disease and diabetes. *JAMA Cardio* 1(6)**,** 692-699. doi: 10.1001/jamacardio.2016.1884

123. WHO. 2014. Global nutrition targets 2025: low birth weight policy brief Geneva. *World Health Organization* [Online].
124. Wood, A.R., Esko, T., Yang, J., Vedantam, S., Pers, T.H., Gustafsson, S., et al. (2014). Defining the role of common variation in the genomic and biological architecture of adult human height. *Nature Genetics* 46(11)**,** 1173-1186. doi: 10.1038/ng.3097.
125. Yang, J., Benyamin, B., McEvoy, B.P., Gordon, S., Henders, A.K., Nyholt, D.R., et al. (2010). Common SNPs explain a large proportion of the heritability for human height. *Nature Genetics* 42(7)**,** 565-569. doi: 10.1038/ng.608.
126. Yavorska, O.O., and Burgess, S. (2017). MendelianRandomization: an R package for performing Mendelian randomization analyses using summarized data. *International Journal of Epidemiology***,** dyx034.
127. Zanetti, D., Tikkanen, E., Gustafsson, S., Priest, J.R., Burgess, S., and Ingelsson, E. (2018). Birthweight, Type 2 Diabetes Mellitus, and Cardiovascular Disease: Addressing the Barker Hypothesis With Mendelian Randomization. *Circulation: Genomic and Precision Medicine* 11**,** e002054.
128. Zeng, P., Hao, X., and Zhou, X. (2018). Pleiotropic mapping and annotation selection in genome-wide association studies with penalized Gaussian mixture models. *Bioinformatics* 34(16)**,** 2797-2807. doi: 10.1093/bioinformatics/bty204.
129. Zeng, P., and Zhou, X. (2017). Non-parametric genetic prediction of complex traits with latent Dirichlet process regression models. *Nature Communications* 8(1)**,** 456. doi: 10.1038/s41467-017-00470-2.
130. Zhou, X., Carbonetto, P., and Stephens, M. (2013). Polygenic modeling with Bayesian sparse linear mixed models. *PLoS Genetics* 9(2)**,** e1003264. doi: 10.1371/ journal.pgen.1003264.
131. Zhou, X., and Stephens, M. (2012). Genome-wide efficient mixed-model analysis for association studies. *Nature Genetics* 44(7)**,** 821-824. doi: 10.1038/ng.2310.
